# Machine Learning–Assisted Bio‐Interfacial Engineering Resolves Structural–Functional Conflicts in Nanocomposites

**DOI:** 10.1002/adma.202518806

**Published:** 2026-03-25

**Authors:** Hao Wang, Xianfeng Chen, Peiyao Yan, Siqi Liu, Biaobiao Yan, Junhua Kong, Siew Lang Teo, Kai Jin, Jie Zhang, Ping Koy Lam, Chaobin He

**Affiliations:** ^1^ Department of Materials Science and Engineering National University of Singapore Singapore Singapore; ^2^ Institute For Materials Research and Engineering(IMRE) Agency for Science, Technology and Research(A*STAR) 2 Fusionopolis Way, Innovis Singapore Singapore; ^3^ School of Materials Science and Engineering Ocean University of China Qingdao China; ^4^ Department of Civil and Environmental Engineering National University of Singapore 1 Engineering Drive 2 Singapore Singapore; ^5^ Institute of Applied Mechanics College of Aeronautics and Astronautics Taiyuan University of Technology Taiyuan PR China

**Keywords:** bio‐interfacial engineering, machine learning–guided optimization, multifunctional nanocomposites, strength–toughness optimization

## Abstract

Delivering nanocomposites that combine high strength, toughness, and multifunctionality remains a major challenge, as conventional trial‐and‐error and design‐of‐experiments approaches cannot efficiently resolve trade‐offs in high‐dimensional design spaces. We introduce a machine‐learning–assisted bio‐interfacial design framework integrating Gaussian‐process surrogates, Pareto set learning, and active learning to explore composition–processing spaces under calibrated uncertainty. The workflow converges after nearly 60 experiments, reducing experimental count, project duration, and cost by 74%–85% relative to conventional methods, thereby accelerating design cycles and expanding Pareto coverage. Guided by this approach, we realize mycelium–graphene composites with strength >58 MPa, toughness >6 MJ/m^3^, and levitation >0.14 mm, showing that strength can be maintained while toughness is significantly enhanced and multifunctionality unlocked. Mechanistic analyses reveal nanosheet‐pinned, hierarchically entangled interfaces where hydrogen‐bonded junctions enable reversible nanosheet sliding, crack deflection, and adaptive stress transfer. These architectures impart levitation control, laser‐driven actuation, and self‐healing. Extension to MXene systems yields composites with enhanced resilience and electromagnetic interference shielding above 40 dB, confirming the generality of the strategy. Together, these advances define a scalable and sustainable paradigm for the accelerated discovery of robust, multifunctional nanocomposites.

## Graphical abstract

A machine learning‐guided bio‐interfacial design strategy resolves the long‐standing strength–toughness–functionality trade‐off in nanocomposites. By efficiently mapping high‐performance regions in the composition–processing space, the approach delivers hierarchically entangled, nanosheet‐pinned architectures that combine mechanical robustness, diamagnetic levitation, and versatile functionalities, establishing a general pathway toward multifunctional, sustainable composites.

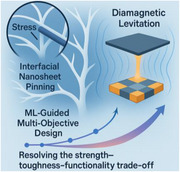

## 1 Introduction

Breaking the long‐standing strength–toughness–functionality conflict represents a central challenge for the next generation of polymer nanocomposites [1, 2, 3]. While the incorporation of rigid 2D fillers such as graphene or MXene markedly improves stiffness and strength, it frequently compromises ductility, interfacial stability, and long‐term reliability [4, 5, 6, 7, 8, 9]. This trade‐off is particularly detrimental for bio‐based or soft‐matrix systems, where sustainability, structural adaptability, and multifunctionality are simultaneously required. Existing composites, therefore, remain trapped within a narrow design space, unable to deliver both robustness and multifunctionality.

Interface engineering strategies including covalent grafting, surface functionalization, and chemical crosslinking have been widely employed to improve stress transfer [10, 11, 12, 13, 14]. While effective in certain cases, these approaches are matrix‐specific, irreversible, and fail to construct adaptive, multiscale architectures capable of dissipating energy under load. As a result, they reinforce stiffness but undermine compliance, limiting their utility in applications where resilience and functional responsiveness must coexist.

Recent progress in mycelium‐based composites shows that fungal species, substrate conditions, and growth environments can effectively regulate density, stiffness, and toughness in bio‐derived structural materials [15, 16, 17]. Architected and living mycelium systems further demonstrate enhanced performance through templated geometries [18] and fiber dispersions with humidity‐responsive actuation [19]. Mycelium has also been explored as a sustainable scaffold for flexible electronics and functional interfaces [20], and conductive CNT–mycelium hybrids illustrate the adaptability of fungal networks [21]. While these studies demonstrate the versatility of fungal scaffolds, they primarily target conductivity, insulation, or basic reinforcement and do not address simultaneous enhancement of strength, toughness, and actuation‐related functionality. Hence, we propose a bio‐assisted nanosheet‐pinning strategy, in which mycelial hyphae physically anchor nanosheets within hierarchically entangled, hydrogen‐bonded polymer networks. This bio‐assembled interface produces a pinned‐and‐bridged topology that enables dynamic stress transfer, reversible nanosheet sliding, and crack deflection. These mechanisms are seldom attainable in conventional nanofiller systems.

Machine learning (ML) guided multi‐objective optimization has emerged as a powerful tool for accelerating materials design. Recent studies have applied multi‐objective Bayesian optimization (MOBO) and active learning (AL) to functional materials, printing inks, and architected structures, typically using Gaussian‐process (GP) surrogates with scalarized or hypervolume‐based acquisition functions to identify Pareto‐optimal candidates [22, 23, 24, 25]. Although effective, these methods treat the Pareto front as a discrete set of points and must be rerun when preferences change, as the absence of a continuous preference‐to‐design mapping makes the global trade‐off landscape difficult to interpret and limits rapid retargeting of evolving mechanical–functional requirements. To overcome this limitation, we introduce a preference‐conditioned Pareto Set Learning (PSL) framework that learns a continuous mapping from preference vectors to optimal MGCs formulations and processing conditions, reconstructing an approximate Pareto manifold rather than isolated solutions. Trained via an augmented Tchebycheff scalarization of a GP surrogate and coupled to an uncertainty‐aware AL loop, PSL enables sample‐efficient, interpretable exploration of the high‐dimensional composition–processing space and supports flexible, preference‐guided navigation of strength–toughness–levitation trade‐offs.

## 2 Results and Discussions

### 2.1 The Design Strategy of Bio‐Assisted Nanosheet‐Pinned Interface

**FIGURE 1:**
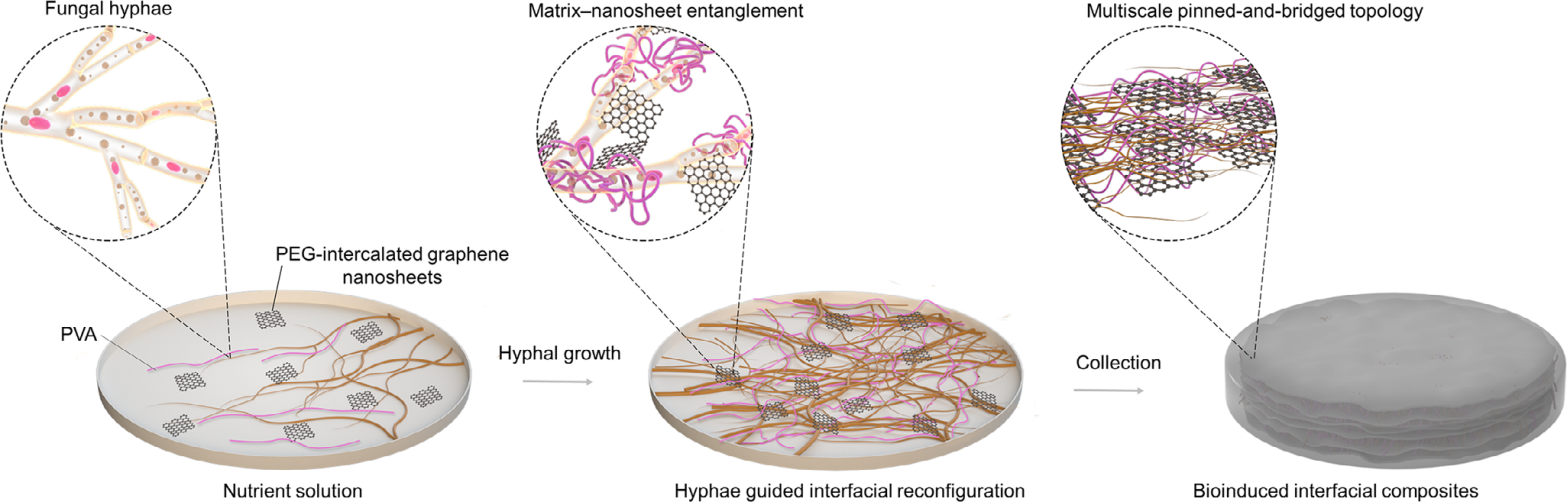
Bio‐assisted construction of nanosheet–polymer interfaces. Schematic of fungal hyphae–guided interfacial structuring. In a nutrient‐rich polymer solution containing PEG‐intercalated graphene nanosheets and PVA, inoculated hyphae proliferate and intertwine with polymer chains and nanosheets. This growth‐driven reconfiguration generates spatial confinement and multiscale entanglement, forming pinned‐and‐bridged interfacial topologies. The resulting biohybrid networks are harvested and dried into robust nanocomposites.

Bio‐inspired structuring opens new avenues for interfacial engineering in nanocomposites. Mycelium, the filamentous network of fungi, naturally assembles into branched, porous, and hierarchically entangled networks [16, 26] that are difficult to reproduce by conventional chemical crosslinking or surface modification. Leveraging this property, we establish a bio‐assisted nanosheet‐pinning strategy in which *Schizophyllum commune* hyphae physically pin PEG‐intercalated graphene nanosheets (PEG–graphene) into a dynamically evolving fibrous scaffold (Figure 1). This growth‐driven reorganization yields a pinned‐and‐bridged topology with distributed anchoring and multiscale entanglement, thereby creating load‐sharing pathways beyond conventional polymer–nanofiller adhesion.

To validate the effectiveness of the biologically programmed interfaces, we systematically characterize the microstructure and interfacial chemistry of the mycelium–graphene composites (MGCs) (Note S2). Optical microscopy and 3D profilometry reveal a continuous hyphal scaffold with pronounced microscale roughness, providing interlocking topographies that promote mechanical anchoring between the polymer matrix and graphene sheets (Figure S2). FTIR spectra indicate PEG intercalation between graphene layers, with characteristic O─H (∼3400 cm^−1^), C─H (∼2900 cm^−1^), and C─O─C (∼1100 cm^−1^) peaks, which are absent in pristine graphene, evidencing hydrogen bonding and van der Waals interactions (Figure S4). High‐resolution microscopy further shows preferential localization of graphene sheets along hyphal surfaces, forming spatially confined, topologically interlocked domains (Figure S5). Importantly, this bio‐directed assembly yields cohesive, freestanding composites without chemical crosslinkers or thermal curing, highlighting both the scalability and sustainability of the process.

### 2.2 Machine Learning Guided Growth

**FIGURE 2:**
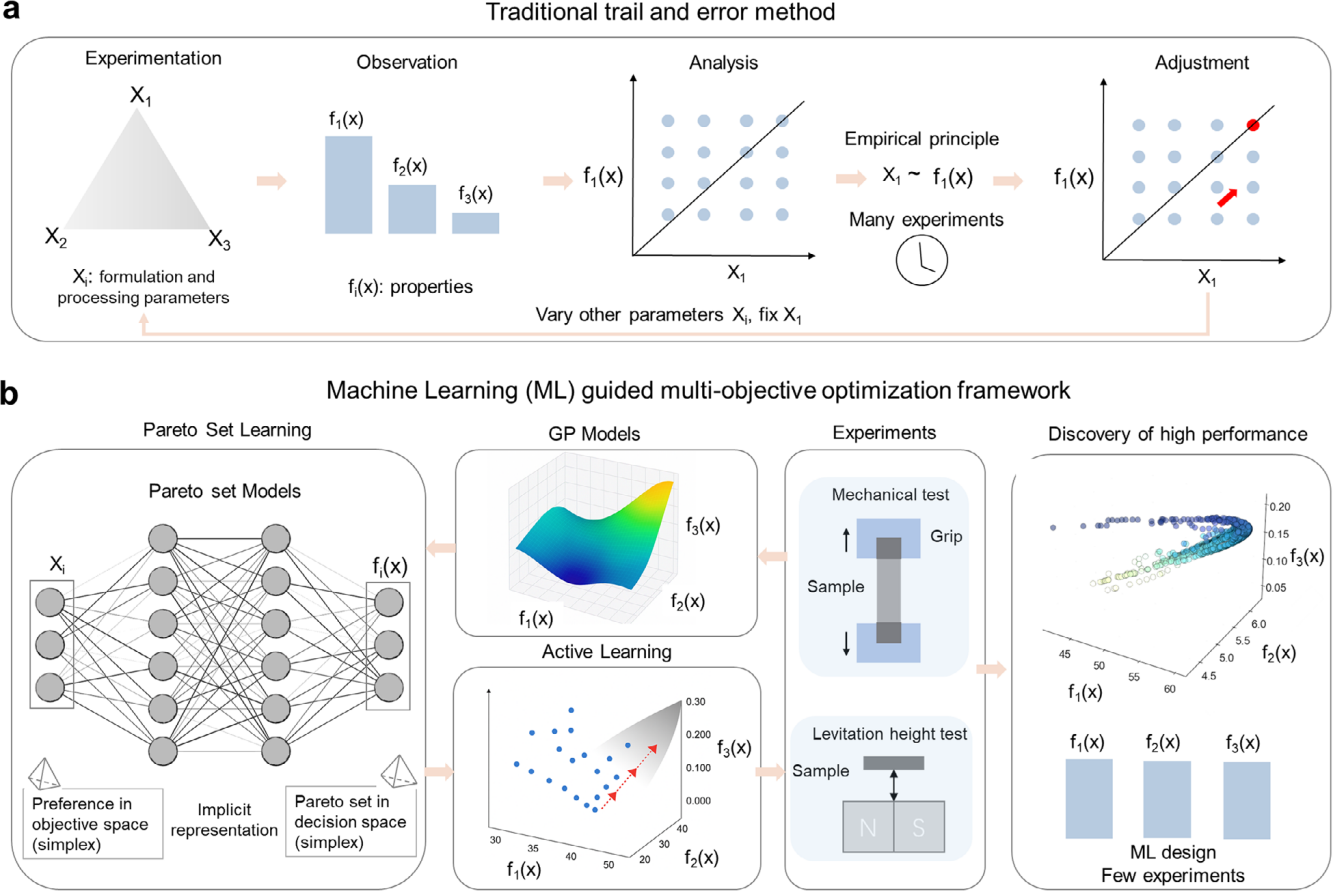
Comparison between traditional trial‐and‐error design and machine learning (ML)‐guided multi‐objective optimization. (a) Traditional trial‐and‐error and design‐of‐experiments (DoE) approaches rely on exhaustive and uniform sampling of formulation and processing conditions. These strategies often require hundreds of experiments even for three‐variable (x_i_) systems, typically altering one factor f_i_(x) at a time. Such conventional approaches test many compositions that lie far from optimal regions, yielding little actionable insight. In mycelium‐based composites, where each experiment requires multi‐day growth and intensive processing, this inefficiency becomes prohibitive and fails to capture nonlinear, multi‐objective trade‐offs among strength, toughness, and levitation performance. (b) The ML‐based framework integrates Gaussian Process (GP) regression, preference‐guided Pareto Set Learning (PSL), and Active Learning (AL) to efficiently explore the design space. Instead of uniformly scanning all combinations, the model generates candidate formulations (x_i_) based on performance preference vectors (λ) and iteratively selects informative, high‐value solutions f_i_(x). This guided search significantly reduces experimental load and accelerates convergence toward high‐performance regions, enabling rapid identification of optimal formulations beyond the reach of conventional methods.

The bio‐assisted interfacial strategy provides a robust structural basis for multifunctional composites. However, the nonlinear design space defined by composition, interfacial geometry, and processing parameters remains difficult to optimize empirically [27, 28]. Traditional formulation approaches, including trial‐and‐error heuristics and classical design of experiments (DoE), are limited in efficiency and scalability (Figure 2a). One‐factor‐at‐a‐time adjustments fail to capture variable interactions, and even classical DoE requires a rapidly increasing number of runs as the number of variables and factor levels grows. For instance, three variables at five levels already demand 5^3^ = 125 conditions, which is prohibitive when each cycle involves multi‐day biological growth and labor‐intensive processing. Moreover, conventional linear regression models and black‐box optimization techniques [29] struggle to capture nonlinear couplings under data‐scarce conditions, particularly when reconciling conflicting objectives f_i_(x) such as strength, toughness, and diamagnetic performance.

To address these limitations, we introduce a machine‐learning–guided optimization framework that combines Gaussian process (GP) regression, Pareto set learning (PSL), and active learning (AL) (Figure 2b). In contrast to conventional exploration strategies such as one‐factor‐at‐a‐time tuning, grid‐based screening, or static DoE designs, our framework leverages calibrated GP uncertainty to adaptively focus sampling on the most informative and promising regions of the design space. Preference vectors (λ) define target trade‐offs among strength, toughness, and diamagnetic performance, allowing PSL to generate candidate solutions that lie near the Pareto front. These candidates are prioritized by an acquisition function that balances exploration and exploitation, and the model is retrained with experimental feedback after each iteration. As a result, the number of experiments required to approach the non‐dominated solution set is reduced by an order of magnitude, convergence to high‐performance regions is accelerated, and the framework enables systematic navigation of compositional regimes that would be impractical to identify through exhaustive or heuristic searches.

### 2.3 Pareto Set Learning (PSL) for Multi‐Objective Optimization

We employ a design‐of‐experiments (DoE) strategy to select 30 representative formulations that systematically span a 3D input space. The inputs include the polymer matrix content, the graphene concentration, and the mycelium growth duration, covering both compositional and processing variables. Each selected formulation is experimentally characterized for strength, toughness, and diamagnetic levitation height, together defining the multi‐objective performance space of the composites.

To establish the predictive capability of the Gaussian Process (GP) models, we adopt a hold‐out validation scheme, training on 25 samples and testing on 5 independent data points. The predictive performance of the GP models is summarized in Figure S6 (Note S3.1). For strength, the model achieves R^2^ = 0.89 with errors within ±6 MPa and calibrated uncertainties of 2.2–5.1 MPa. The toughness model yields R^2^ = 0.70 and reliably separates high‐ and low‐toughness regimes with uncertainty <0.5 MJ/m^3^. Levitation is predicted most accurately (R^2^ = 0.96), showing consistent agreement across the test set. These results confirm that the GP models are well‐calibrated, capture key structure–property relationships, and serve as a credible predictive model for guiding subsequent multi‐objective optimization.

Building on this foundation, we implement Pareto Set Learning (PSL) to navigate the multi‐objective landscape (Note S3.2). Using the trained PSL model, 1000 candidate solutions are generated by sampling preference vectors λ. In the compositional–process domain (Figure S7a), PSL generates a diverse and well‐distributed set of candidates, indicating efficient exploration of the design space. In the predicted performance domain (Figure S7b,c), the candidates map onto a well‐defined Pareto front, which clearly exposes the trade‐offs among strength, toughness, and levitation. The presence of smooth gradients along the front suggests consistent generalization and reveals tunable pathways for reconciling competing objectives. Importantly, PSL does not merely interpolate known data but expands the accessible performance envelope, offering actionable guidance for experimental prioritization.

### 2.4 Active Learning

While PSL provides an efficient means to map preference vectors to approximate Pareto‐optimal solutions, its predictive accuracy is inherently constrained by the limited initial dataset. As a result, some regions of the feasible design space remain underexplored, and the Pareto front reconstructed by PSL may not fully capture the attainable performance envelope. To further enhance the efficiency and performance of the optimization process, we integrate Active Learning (AL) with Bayesian Optimization (BO), allowing the model to iteratively query the most informative experiments, reduce predictive uncertainty, and progressively refine the attainable performance envelope (Note S3.3).

**FIGURE 3:**
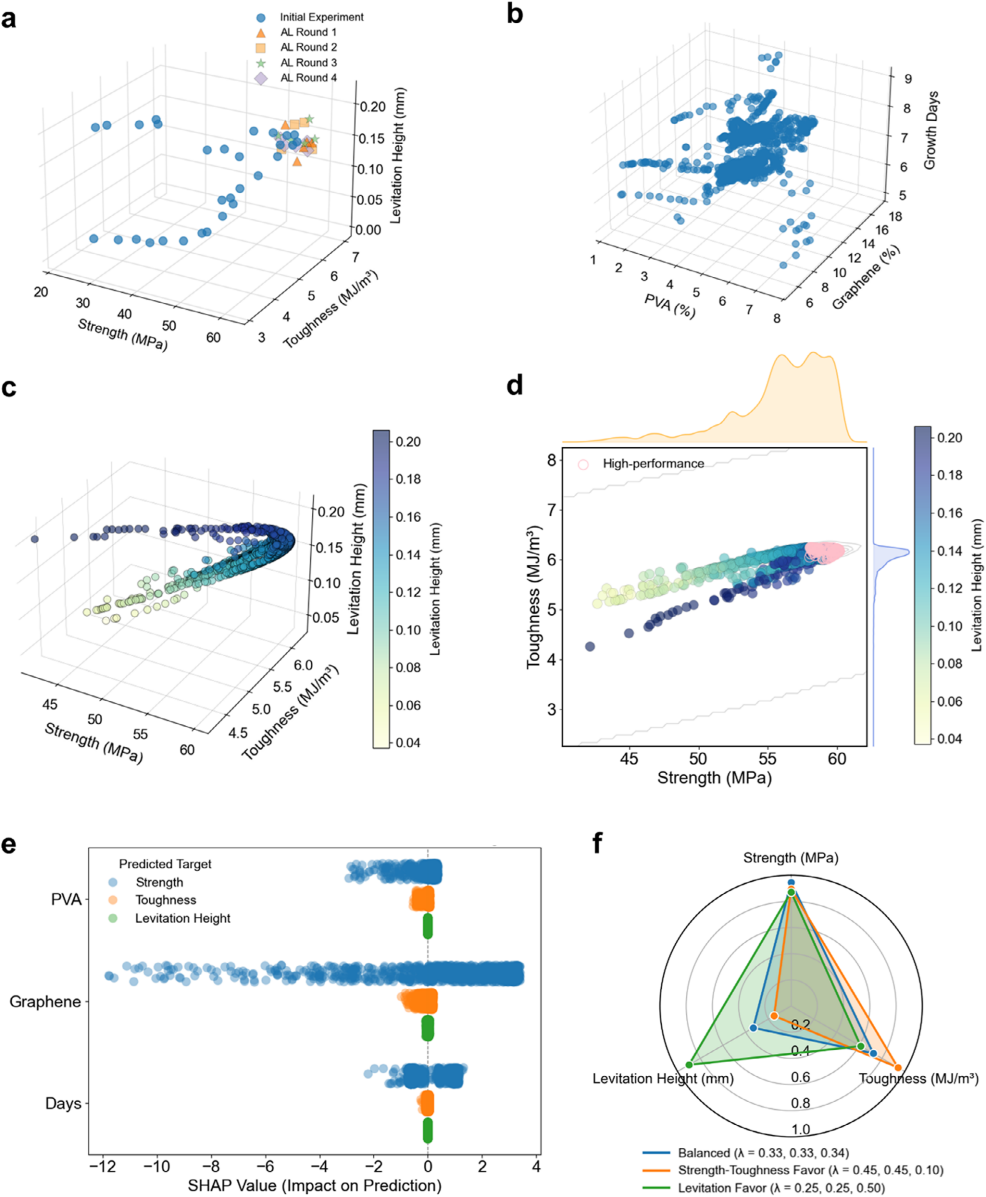
Active Learning (AL). (a) Evolution of solutions during four AL rounds. Initial experimental data (blue circles) are progressively expanded by AL‐selected samples: Round 1 (orange triangles), Round 2 (yellow squares), Round 3 (green stars), and Round 4 (purple diamonds). Optimized PSL solution set after AL: (b) Composition‐space distribution, (c) Performance space. (d) High‐performance solutions (highlighted in pink) simultaneously achieve strength >58 MPa, toughness >6.0 MJ/m^3^, and levitation height >0.14 mm, which a regime entirely absent from the initial solution set. Marginal density plots demonstrate significant rightward and upward shifts in strength and toughness distributions, indicating model reorientation toward optimal regions. Kernel density estimation is used to compute marginal distributions. (e) Unified SHAP analysis across three targets. Beeswarm plot showing SHAP values of input features (PVA, graphene, and growth duration) for strength (light blue), toughness (orange), and levitation height (green). Graphene exhibits the strongest positive influence, PVA contributes weakly, and growth duration has variable effects across objectives. (f) Preference‐guided selection of candidate solutions within the high‐performance region. Radar chart comparing normalized predicted values for three representative solutions based on distinct preference vectors (λ): balanced (blue), strength–toughness oriented (orange), and levitation oriented (green). Each preference vector yields a distinct trade‐off configuration tailored to specific design priorities.

The evolution of candidate solutions over four AL rounds is summarized in Figure 3a and Figure S8a–e. From the initial dataset (Figure S8a), each iteration systematically expands the Pareto front toward higher strength, toughness, and levitation height. After four rounds (Figure 3a), the selected formulations (triangles, squares, stars, and diamonds) converge within the Pareto‐optimal region, achieving simultaneous performance gains above 58 MPa in strength, 6 MJ/m^3^ in toughness, and 0.14 mm in levitation height. This progression is quantified by hypervolume indicator (HVI) analysis (Figure S9). The first round yields a sharp increase of 22.09%, reflecting the model's ability to rapidly identify non‐obvious high‐value regions. Later rounds generate smaller but consistent improvements (3.16% and 3.54%), and the plateau in Round 4 indicates convergence. Thus, four AL iterations are sufficient to capture the optimal design space while minimizing experimental effort.

We visualize the Pareto solutions after four AL rounds in Figure 3b,c. In the compositional space (Figure 3b), the solutions populate a broad conical region defined by PVA content, graphene ratio, and growth duration, with clustering near 7–8 days of culture. This pattern indicates that the model extrapolates across the feasible design space while respecting physical growth constraints. In the performance space (Figure 3c), the predicted solutions delineate a continuous, nonlinear Pareto surface that captures the intrinsic trade‐off between strength and toughness, with levitation height increasing smoothly along the surface.

To interpret these outcomes, PSL‐derived solutions are projected into the performance space (Figure 3d). Relative to the initial predictions (Figure S7c), the solution set shows markedly improved coverage in the strength–toughness domain, with levitation height encoded by color. The Pareto surface expands into the upper‐right region, where several formulations simultaneously achieve strength above 58 MPa, toughness above 6 MJ/m^3^, and levitation height above 0.14 mm (highlighted in pink). Marginal density plots confirm rightward and upward shifts in strength and toughness, evidencing new performance regimes captured through AL‐guided sampling. Notably, the solution manifold evolves from a narrow diagonal band into a multi‐layered Pareto front, indicating improved diversity and resolution of trade‐offs. This unfolding of the performance space highlights the synergy between preference‐driven PSL and data‐efficient AL, which transforms the framework from a local approximation into a global optimization strategy capable of uncovering high‐performance regions inaccessible to conventional exploration.

To further assess the robustness of the PSL–AL framework, we track the evolution of surrogate uncertainty during optimization (Note S3.4). After four AL rounds, the predictive standard deviation (σ) of the GP models decreases by 42.0% for strength and 39.6% for toughness, while levitation uncertainty remains consistently low. This trend is evident in both σ distributions and composition‐resolved uncertainty maps (Figures S10 and S11), indicating enhanced model confidence and more uniform exploration of the design space. These results demonstrate that the closed‐loop PSL–AL strategy not only broadens the Pareto front but also strengthens surrogate reliability and generalizability in high‐performance regions.

Moreover, a unified SHAP analysis is performed across all three objectives to identify the relative contributions of compositional and processing variables (Figure 3e; Note S3.5). Graphene emerges as the dominant factor, with SHAP values from −6 to +4, exerting strong positive effects on strength and moderate influences on toughness and levitation height. Growth duration shows a dual role, enhancing strength (−4 to +4) but exerting neutral or negative effects on toughness and levitation. The findings establish modified graphene as the key reinforcement factor, while revealing growth duration as a critical determinant of performance fluctuations that merit systematic exploration.

To further demonstrate the practicality of the PSL–AL framework, we apply user‐defined preference vectors (λ) to steer candidate formulations within the high‐performance Pareto region (Figure S12, Table S1, and Note S3.6). Radar plots (Figure 3e) show that each selected formulation expresses a distinct trade‐off profile aligned with its design priority. For example, a balanced design (λ = 0.35, 0.35, 0.30) yields 60.1 MPa strength, 6.20 MJ/m^3^ toughness, and 0.16 mm levitation height; a mechanically biased design (λ = 0.45, 0.45, 0.10) maximizes strength and toughness (60.0 MPa, 6.22 MJ/m^3^) with slightly reduced levitation (0.15 mm); and a levitation‐oriented design (λ = 0.25, 0.25, 0.50) enhances levitation (0.17 mm) while maintaining robust mechanics (59.9 MPa, 6.19 MJ/m^3^). Notably, the PSL model consistently identifies an optimal growth duration of 8 days across all cases, underscoring its ability to jointly optimize compositional and processing variables. This preference‐resolved analysis demonstrates the interpretability and flexibility of the PSL–AL framework. Rather than converging on a single optimum, it generates a programmable map of the composition–performance space, enabling rational, customizable decision‐making and reducing experimental burden in the development of multifunctional composites.

### 2.5 Mechanical Experimental Validation and Mechanism Analysis

To validate the predictive capability of the PSL–AL framework, we synthesize and test three representative MGCs formulations selected from distinct regions of the Pareto front using user‐defined preference vectors (λ): a balanced design, a strength–toughness favored design, and a levitation favored design (Table S1).

**FIGURE 4:**
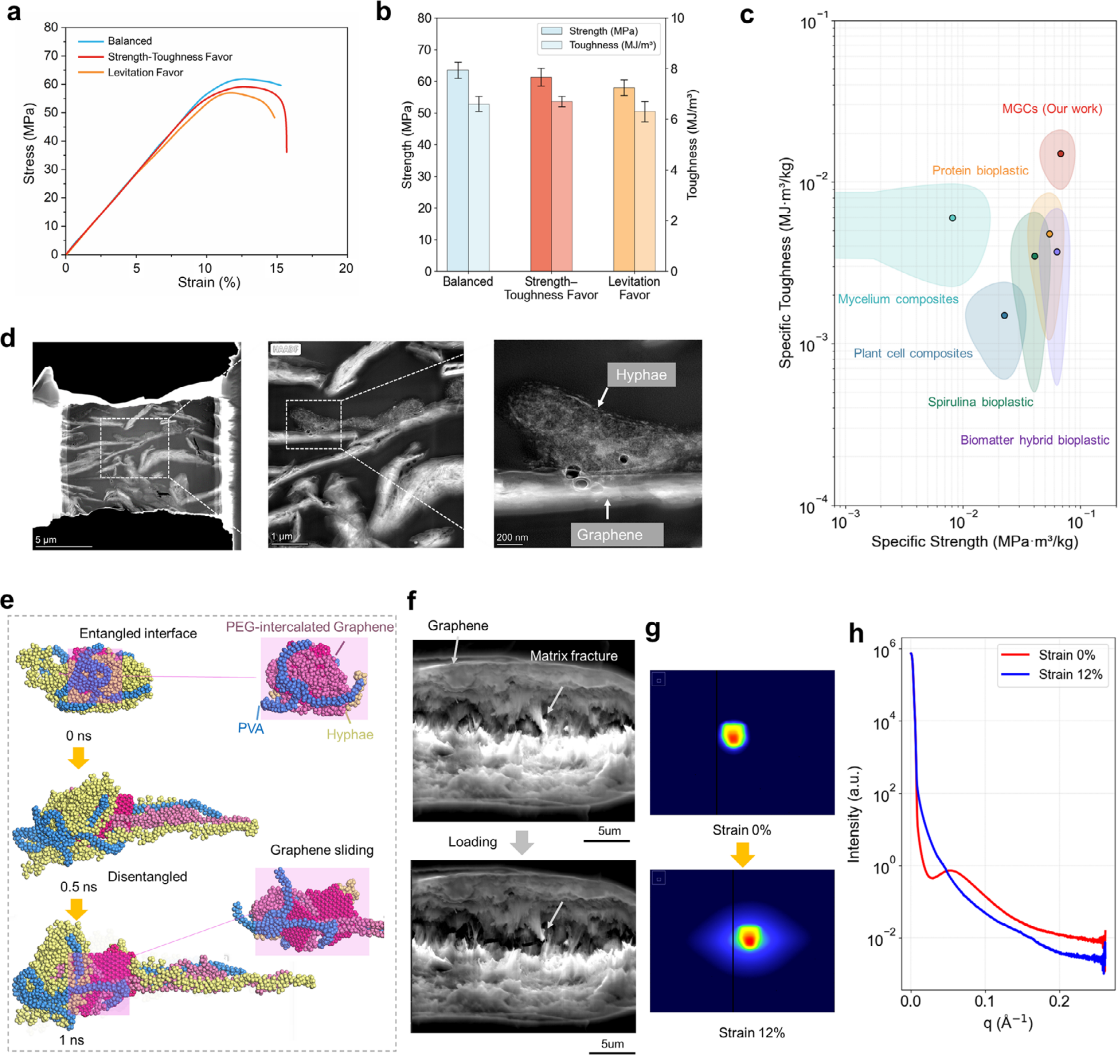
Mechanical validation and interfacial mechanism of MGCs. (a) Stress–strain curves of three MGC formulations selected from distinct regions of the predicted Pareto front: balanced (blue), strength–toughness favoring (red), and levitation favoring (orange). Each formulation corresponds to a specific preference vector λ derived from the PSL model. (b) Comparison of tensile strength and toughness for the three representative samples. (c) Ashby plot benchmarking the specific strength and toughness of MGCs against representative bio‐based composites, including mycelium, protein, plant cell, spirulina, and hybrid bioplastics [17, 30, 31, 32, 33, 34, 35]. MGCs occupy a distinct region with simultaneous enhancement in both metrics, highlighting their mechanical efficiency. (d) FIB‐TEM images showing a hierarchically entangled, pinned‐and‐bridged interface between graphene nanosheets and fungal hyphae. (e) Molecular dynamics (MD) simulations illustrating strain‐induced disentanglement and graphene sliding within the PVA–hyphae interface. (f) In situ tensile SEM characterization of the MGCs. Real‐time deformation reveals graphene pull‐out, polymer–hypha fibrillation, and progressive interfacial debonding under applied load. These features directly validate the dynamic nanosheet‐pinned sliding mechanism responsible for the observed strengthening and toughening behaviour. (g) 2D SAXS patterns at 0% and 12% strain, showing increased anisotropy upon deformation. (h) Corresponding 1D SAXS intensity profiles, with enhanced low‐q scattering after stretching, indicating nanoscale structural rearrangement.

As shown in Figure 4a,b, the balanced formulation delivers a tensile strength of 63.5 MPa with an elongation at break of 14.5%, confirming effective integration of strength and ductility. The strength–toughness favored sample displays a pronounced post‐yield plateau and a fracture toughness of 6.7 MJ/m^3^ while retaining a peak strength of 61.3 MPa. The levitation favored design shows earlier strain softening, yielding a lower strength of 58.0 MPa and a moderate toughness of 6.3 MJ/m^3^. These distinct responses are consistent with the prescribed design priorities, confirming that the PSL–AL framework reliably tailors performance profiles along multiple objectives.

The Ashby plot of specific strength versus specific toughness (Figure 4c; Table S2) places MGCs in a previously inaccessible performance domain relative to representative bio‐based materials, including conventional mycelium composites [16, 17, 18, 30, 31], plant‐cell composites [32], hybrid bioplastics [33], protein‐based bioplastics [34], and spirulina‐derived films [35]. MGCs deliver simultaneous improvements in strength and toughness while maintaining low density, thereby defining a new high‐performance window that exceeds both natural analogues and current synthetic bioplastics. Beyond the bio‐based landscape, the MGCs also surpass several reported graphene‐reinforced polymer composites, which typically occupy lower regions of the Ashby space in both specific strength and specific toughness (Figure S14a and Table S3). When situated within the broader material landscape (Figure S14b), the MGCs reside at the transition between natural cellular solids and lightweight engineered composites, demonstrating a combination of strength and density characteristic of advanced structural materials. These results show that the proposed ML framework enables targeted exploration of competing mechanical objectives and facilitates access to previously unrealized regions of the design space, yielding bio‐derived nanocomposites with structural efficiency that rivals state‐of‐the‐art lightweight materials.

Control experiments systematically compare the respective contributions of nanosheet reinforcement and bio‐assembled interfaces (Figure S13). Blended PVA–PEG–graphene composites achieve high strength but fail catastrophically, whereas mycelium‐assembled PVA composites improve toughness at the expense of strength. In contrast, MGCs that integrate nanosheet pinning with hyphal interlocking achieve concurrent gains in strength and toughness, occupying a distinct high‐performance domain in the Ashby plot (Figure 4c). This synergy between graphene reinforcement and bio‐programmed interfacial structuring resolves the classical incompatibility between strength and toughness, defines a previously inaccessible design space, and establishes a new paradigm for structural biocomposites.

We investigate the interfacial mechanisms underlying the balanced‐design MGCs. Electron microscopy (Figure 4d; Figure S15) reveals fungal hyphae wrapping around graphene nanosheets to form hierarchically entangled, pinned‐and‐bridged junctions that promote mechanical interlocking. Complementary FTIR spectra (Figure S16) show broadened and red‐shifted O─H stretching together with shifts in C═O and C─O─C vibrations, consistent with hydrogen‐bonded networks among PVA, hyphae, and graphene. These interactions, predominantly arising from hydroxyl, carboxyl, and amine groups in fungal cell wall polysaccharides and PVA chains, create dynamic yet robust adhesion at the bio‐derived polymer/graphene interface. PEG acts as a flexible intercalating layer that couples PVA chains with graphene surfaces, reinforces multisite hydrogen‐bonding networks, and improves interfacial adaptability under mechanical loading.

To elucidate the interfacial deformation mechanisms suggested by experiments, molecular dynamics (MD) simulations are performed (Figure 4e; Note S6). The simulations show that graphene nanosheets undergo strain‐dependent relative sliding, while reversible polymer–hypha disentanglement provides temporary load bridging. These nanoscale processes dissipate energy and delay the onset of interfacial cracking. To connect these atomistic events with mesoscale load transfer, finite‐element (FE) simulations are conducted on representative microstructural domains (Note S7). The resulting stress maps (Figure S19) reveal an evolution from edge‐localized pinning at small strain to pronounced interfacial shear as deformation increases. This redistribution forms continuous load‐bearing pathways aligned with the tensile direction, directly linking MD‐predicted sliding behavior to the macroscopic toughening response. In situ tensile SEM observations further corroborate this mechanism. Graphene pull‐out, matrix rupture, and shear‐induced fibrillation (Figure 4f) are consistent with frictional sliding and progressive interfacial debonding during loading. Complementary SAXS data confirm the adaptive structural response: at 12% strain, the initially isotropic nanostructure becomes anisotropic and shows intensified low‐q scattering (Figure 4g,h), indicating nanosheet alignment and domain reorganization that redistribute stress and postpone failure.

These observations establish a cooperative interfacial mechanism in which fungal hyphae, PEG–graphene hybrids, and PVA chains assemble non‐covalent yet mechanically robust junctions through multiscale entanglement, hydrogen bonding, and interfacial adsorption. Under mechanical loading, coordinated nanosheet sliding, polymer‐chain disentanglement, and interfacial reorganization dissipate energy, deflect cracks, and sustain stress transfer, enabling simultaneous increases in strength and toughness. This behavior is consistent with nanoconfined polymer responses, where restricted chain mobility and stress redistribution enhance stiffness and delay fracture [36, 37, 38], providing a molecular‐level rationale for the performance of our bio‐assisted, nanosheet‐pinned architecture.

### 2.6 Magnetic Levitation Performance Analysis

**FIGURE 5:**
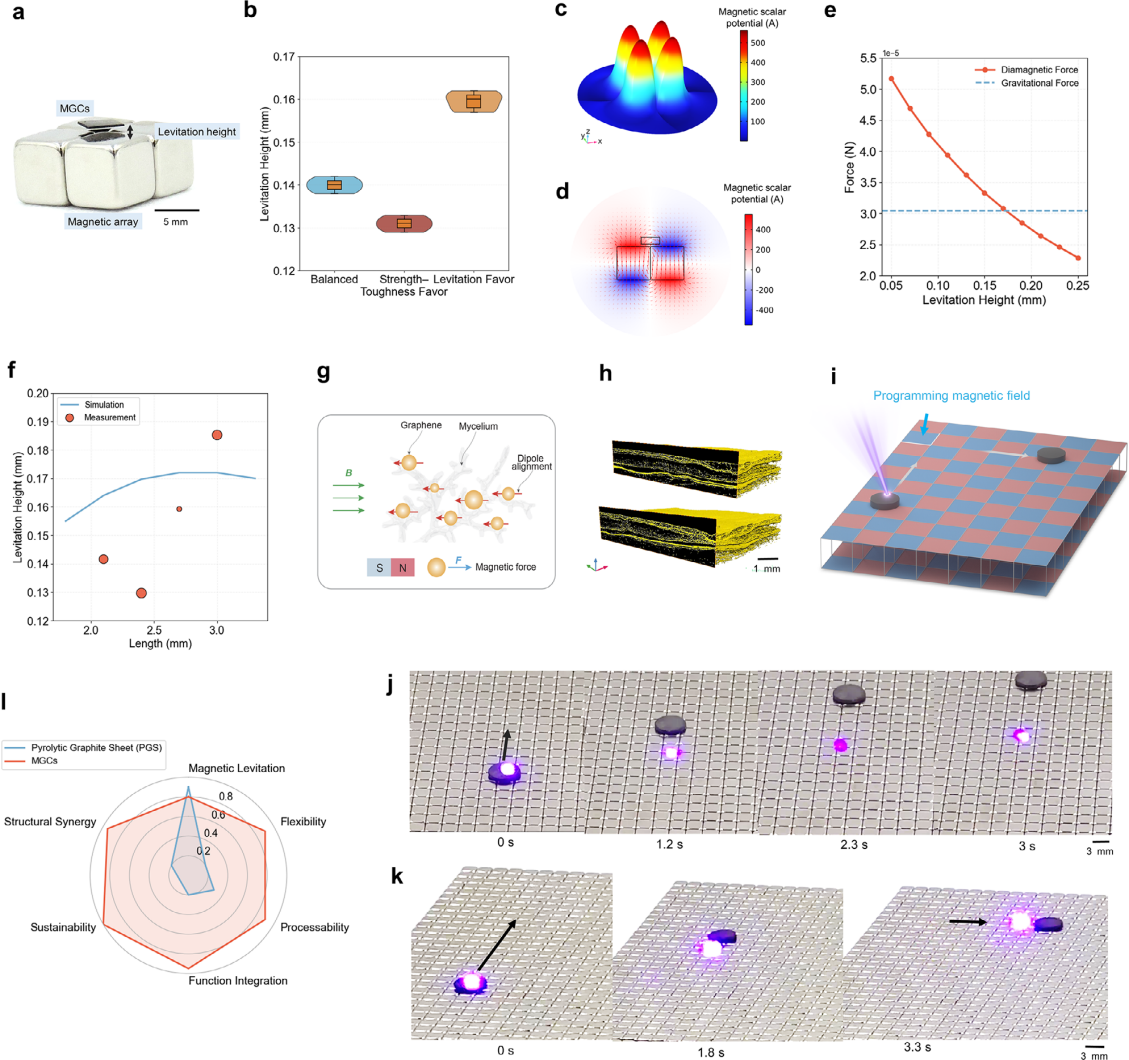
Magnetic levitation and programmable locomotion of MGCs. (a) Optical photograph of a balanced‐design MGCs plate levitating above a permanent magnet array. The levitation height is defined as the vertical air gap between the magnet surface and the underside of the MGCs, as measured using a laser distance sensor. (b) Violin plots of levitation height for three groups of MGCs (n = 6 per group) optimized by different Pareto‐guided design strategies. The levitation‐oriented formulation achieves the highest levitation (∼0.165 mm), confirming tunable functional performance. (c) Finite‐element (FE) simulation of the magnetic scalar potential, showing a quad‐peak confinement field that generates vertical repulsion. (d) Simulated 2D cross‐sectional field distribution with vector arrows highlighting the upward diamagnetic force acting on the levitated MGCs plate. (e) Simulated relationship between levitation force and distance for a 3 mm plate, indicating equilibrium against gravitational force. (f) Comparison of simulated and experimentally measured levitation heights for square plates of different lateral dimensions (thickness ∼0.6 mm). The circle size represents error range. (g) Schematic illustration of dipole alignment in the composite: graphene nanosheets and mycelium domains form a dipole‐responsive network that interacts with the external magnetic field (B), generating upward diamagnetic force (F). (h) X‐ray microtomography (µCT) reconstructions of MGCs showing an anisotropic lamellar architecture with interlocked mycelium–graphene domains. (i) Schematic illustration of laser‐induced actuation on a programmable magnet array. Localized laser irradiation initiates levitated motion of MGCs plate, while introducing gaps between adjacent magnets reconfigures the magnetic field distribution into confinement wells. The coupling of photothermal driving with anisotropic magnetic confinement enables programmable trajectory control. (j) Sequential optical images of an MGC plate under localized laser irradiation, showing vertical levitation and guided translation across the magnet array. (k) Representative trajectories of MGC actuators under laser irradiation, demonstrating controllable displacement and reorientation. The irradiation position defines the initiation point, while the gap‐modulated magnetic field pattern directs translational and turning behaviors. (l) Radar plot comparing multifunctional performance of MGCs with commercial pyrolytic graphite sheets (PGS). Normalized indices benchmark magnetic levitation, flexibility, processability, functional integration, sustainability, and structural synergy. PGS exhibits strong levitation due to its anisotropic electronic structure but shows limited flexibility, processability, and sustainability. In contrast, MGCs display a balanced multifunctional profile with superior adaptability and structural synergy derived from bio‐assisted interfaces.

The three representative MGCs formulations exhibit stable diamagnetic levitation above an array of N52 NdFeB magnets with alternating polarity (Figure 5a; Video S1). Plate‐shaped samples (2.1–7.0 mm lateral dimension), prepared by micro‐laser cutting, levitate reliably at ambient conditions without external energy input and can undergo rapid, unconstrained translation driven by contactless micro‐forces. To probe functional tunability, levitation heights are quantified for each design (Figure 5b). The levitation favored formulation reaches an average of 0.16 mm, compared with 0.14 mm for the balanced design and 0.13 mm for the strength–toughness favored sample, indicating that the PSL–AL framework tailors functional outcomes alongside mechanical robustness.

Finite element (FE) modeling provides quantitative insight into the levitation mechanism (Note S8). The magnetic array generates a scalar potential landscape with multiple minima that stabilize the suspended plate (Figure 5c). Cross‐sectional analysis reveals an upward diamagnetic force counterbalancing gravity (Figure 5d; Video S2), and the computed force–distance relationship defines the equilibrium height at their intersection (Figure 5e; Figure S22). Experimental measurements across plates of varying lateral size (Figure 5f) align closely with simulations, validating model fidelity. Extending the analysis to circular magnet arrays, the axisymmetric field produces a central confinement minimum consistent with simulated energy landscapes (Figure S23), establishing that MGCs sustain stable levitation across different field geometries.

The Governing Magnetic Body Force Follows the Kelvin Force Density [39], Which for an Isotropic Medium Simplifies to

(1) $$F_{m}=\left(M\cdot \nabla \right)\;B=\frac{\chi }{\mu_{0}}\left(B\cdot \nabla \right)B$$

and, over the entire MGCs volume,

(2) $$F_{B}=\int_{V}\frac{\chi }{\mu_{0}}\left(B\cdot \nabla \right)BdV=\frac{1}{2\mu_{0}}\;\int_{V}\nabla \left(\chi_{x}B_{x}^{2}+\chi_{y}B_{y}^{2}+\chi_{z}B_{z}^{2}\right)dV$$

where *V* denotes the effective diamagnetic volume. Because ∇ ***B*** is negative away from the magnet, the resulting diamagnetic force acts upward, counterbalancing gravity.

The levitation behavior of the MGCs arises from the combined effects of material composition and hierarchical structure (Figure 5g). All constituents of the composite, including the polymer matrix and the mycelium network, possess intrinsically negative magnetic susceptibility (χ), whereas graphene nanosheets exhibit an even smaller susceptibility [40] and therefore dominate the overall diamagnetic response of the system. As a result, the composite is magnetized antiparallel to the external field and experiences a net upward magnetic force ***F***_B_ as described in Equations (1) and (2). The spatial arrangement of the low‐ χ components is particularly important. Graphene nanosheets, owing to their very small susceptibility, strongly influence the effective magnetic volume when positioned near regions of high field gradient. µCT imaging (Figure 5h; Video S3) reveals an anisotropic lamellar architecture formed by horizontally stacked mycelium–graphene layers. This layered configuration reduces the effective density and concentrates graphene‐rich regions close to the magnet surface, which enhances vertical magnetic repulsion and supports stable bulk levitation. Within this structural configuration, mycelium primarily contributes a lightweight, porous scaffold that organizes the nanosheets and lowers the overall density, while the dominant magnetic contribution to levitation arises from the diamagnetism of graphene.

In addition to levitation, MGCs also display intrinsic self‐healing and regenerative capacity. Mycelial hyphae bridge fractured or delaminated regions, re‐establishing bulk integrity even after long storage (Figure S24). Unlike synthetic self‐healing polymers that depend on reversible bond dynamics [37, 41], this biologically driven repair reconstructs both the organic matrix and graphene–polymer interfaces, thereby prolonging service lifetime and enabling circular regeneration pathways.

We assess the reproducibility and environmental stability of the MGCs (Figure S25 and Note S10). Across three independently cultured batches, strength, toughness, and levitation height vary by less than two percent. The composites retain more than ninety‐eight percent of their initial performance after thirty days of ambient storage and maintain above ninety‐seven percent of their properties across a wide humidity range. These results show that the MGCs are structurally robust and that the biosynthetic fabrication process is reliably reproducible.

### 2.7 Programmable Contactless Micro Robot

We demonstrate that MGCs can be engineered into levitated micro robots through synergistic coupling of photo thermal and magnetic stimuli (Figure 5i). FE simulations show that localized laser heating reduces the diamagnetic force, destabilizing equilibrium with gravity and initiating motion (Figure S26, Video S4, and Note S11). Meanwhile, introducing programmed gaps between adjacent magnets reconfigures the field distribution, which creates anisotropic force landscapes that guide displacement trajectories (Figure S27). This dual‐control scheme combines laser‐induced thermal modulation with engineered magnetic potentials, thereby enabling contactless and reconfigurable actuation of lightweight diamagnetic composites.

Under focused irradiation, MGC plates translate smoothly along the magnet array axis (Figure 5j) and can be redirected into lateral pathways by magnetic‐field reprogramming (Figure 5k; Video S5). Their trajectories follow the designed potential wells, where the interplay of photo thermal gradients and spatially varying fields produces directional diamagnetic forces. Trajectory control is achieved by adjusting both the irradiation position and the magnetic‐field distribution, which provides high precision. In contrast to conventional micro robotic systems based on mechanical tethers [42], acoustic fields [43], or uniform magnetic actuation [44], this approach integrates localized heating with programmable field architectures, enabling fully programmable levitation and trajectory control in diamagnetic soft micro robots.

To further illustrate multifunctionality, we benchmark MGCs against pyrolytic graphite sheets (PGS) [45, 46], the most widely used commercial diamagnetic material for magnetic levitation (Figure 5l). PGS exhibits strong levitation due to its highly anisotropic electronic structure, yet its brittleness, low flexibility, and poor processability restrict applications in soft or reconfigurable systems. By comparison, MGCs deliver a more balanced performance, coupling competitive levitation with mechanical compliance, sustainability, and structural adaptability. This advantage arises from their hierarchical bio–graphene architecture, where mycelial networks and polymer chains construct adaptive interfaces, while graphene nanosheets provide stiffness and diamagnetic responsiveness. The synergistic integration enables simultaneous optimization of robustness, functional tunability, and environmental compatibility, performance criteria that are rarely satisfied by conventional diamagnetic materials.

Compared with a conventional DoE approach that requires approximately 400 trials to explore the composition, processing, and property space, the proposed ML framework converges after only about 60 experiments, yielding a 74 to 85 percent reduction in experimental effort, project duration, and cost (Figure S28; Note S12). Beyond improved sequential sample efficiency, the proposed PSL–AL framework generates a denser distribution of high‐performance candidates near the Pareto front and leads to more balanced gains in strength, toughness, and levitation performance. Under the same evaluation budget, benchmark baselines including Random Search, Latin hypercube sampling (LHS), Weighted Multi‐Objective Bayesian Optimization (Weighted‐MOBO), Non‐dominated Sorting Genetic Algorithm II (NSGA‐II), Pareto Efficient Global Optimization (ParEGO), and Expected Hypervolume Improvement (EHVI) show earlier stagnation and more limited Pareto‐front expansion. By contrast, PSL–AL continues to improve throughout the sequential search and yields a broader and higher‐quality Pareto front, with more effective recovery of reference Pareto solutions in the experimentally validated high‐performance region (Figure S29 and Note S13).

### 2.8 Validation of Interface Strategy Universality

**FIGURE 6:**
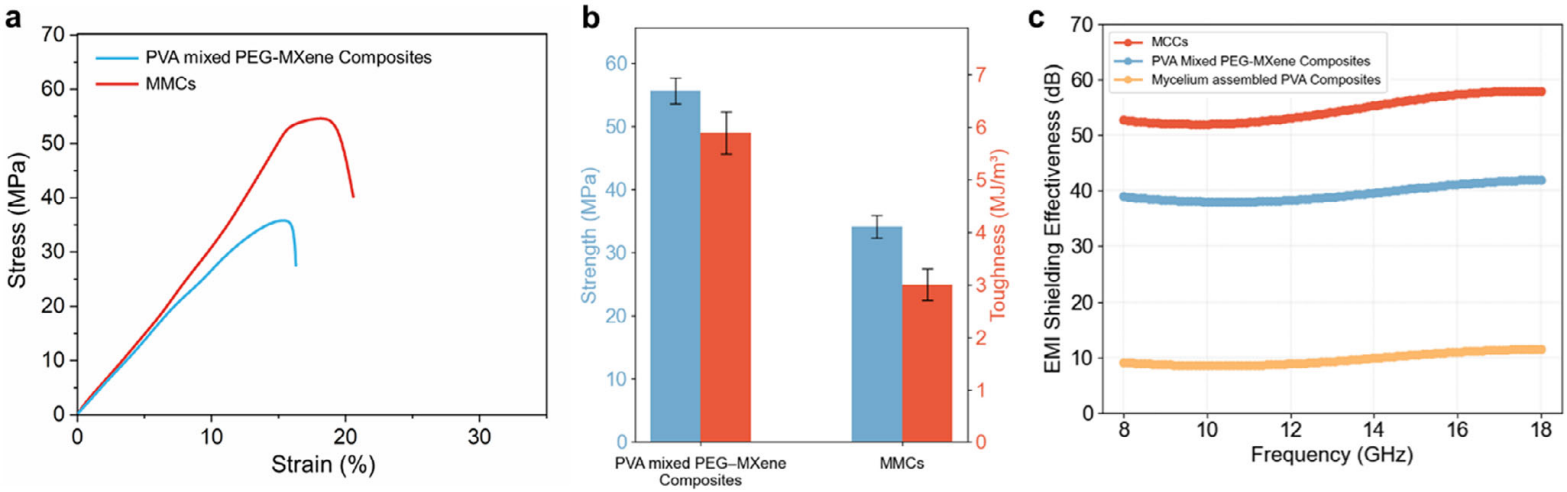
Universality validation of the bio‐interfacial construction strategy. (a) Stress–strain curves of conventionally blended PVA–PEG–MXene composites and mycelium–MXene composites (MMCs), showing that biological assembly produces reinforced architectures absent in physical blending. (b) Quantitative comparison of tensile strength and toughness, demonstrating that MMCs simultaneously achieve high strength and toughness, thereby circumventing the classical strength–toughness trade‐off. (c) Electromagnetic interference (EMI) shielding effectiveness across 8–18 GHz for MMCs, PVA–PEG–MXene composites, and mycelium–PVA composites without MXene. MMCs exhibit superior structural resilience and EMI attenuation, confirming that the bio‐interfacial strategy extends beyond graphene to other classes of two‐dimensional nanomaterials.

The universality of the bio‐interfacial strategy is demonstrated by extending it from graphene to MXene nanosheets, yielding mycelium–MXene composites (MMCs) with markedly enhanced performance compared with conventionally blended PVA–PEG–MXene systems. MMCs achieve a tensile strength of approximately 60 MPa and a toughness of about 6.5 MJ/m^3^ (Figure 6a,b). Elemental mapping (Figure S32) confirms hierarchical integration of Ti‐rich nanosheets with P‐enriched hyphae, showing that fungal filaments pin, bridge, and wrap nanosheets into robust load‐bearing junctions. In addition to mechanical robustness, MMCs deliver strong electromagnetic interference (EMI) shielding of 58–62 dB across 8–18 GHz, outperforming PEG–MXene and mycelium–PVA controls (Figure 6c). These results demonstrate that living growth provides not only structural reinforcement but also functional optimization by reorganizing nanosheet architectures for electromagnetic attenuation.

The successful extension from graphene to MXene establishes this bio‐assisted interfacial strategy as a general design paradigm for multifunctional nanocomposites. By coupling living growth with nanosheet confinement, the approach transcends material specificity, enabling robustness, tunability, and sustainability to emerge from the same principle of biologically programmed interfaces. Its demonstrated adaptability to graphene, MXene, and potentially to other 2D nanomaterials such as boron nitride and clays underscores its promise as a material‐agnostic, scalable strategy for engineering next‐generation structural, energy, and electronic systems.

## 3 Conclusions

This study establishes a machine learning assisted bio‐interfacial design paradigm that resolves the long‐standing challenge of simultaneously achieving high strength, toughness, and functionality in polymer nanocomposites. By integrating Gaussian process surrogates, Pareto set learning, and active learning, the workflow converges after approximately sixty experiments and delivers 74 to 85 percent reductions in experimental count, project duration, and cost relative to conventional design‐of‐experiments approaches, thereby dramatically accelerating materials discovery. Using this data‐efficient strategy, we identify mycelium–graphene composites with strength above 58 MPa, toughness exceeding 6 MJ/m^3^, and stable diamagnetic levitation above 0.14 mm, which a performance profile rarely attainable through empirical screening.

Mechanistic analyses reveal that mycelium growth produces nanosheet‐pinned, hierarchically entangled interface in which hydrogen‐bonded junctions enable dynamic stress‐bridging through reversible nanosheet sliding, crack deflection, and adaptive stress transfer. These cooperative processes explain how the classical strength–toughness trade‐off can be mitigated while simultaneously imparting programmable levitation, laser‐driven actuation, and autonomous self‐healing. The universality of this approach is further confirmed by extending the same interfacial principle to MXene composites, which display enhanced resilience and electromagnetic interference shielding above 40 dB.

These findings define a sustainable and generalizable design paradigm that couples data‐efficient machine learning with bio‐interfacial engineering to create robust, multifunctional nanocomposites. The framework provides a single, scalable route to unite mechanical robustness with precisely tailored functional responses across layered nanomaterials, as exemplified by graphene and MXene, enabling next‐generation structural, energy, and electronic systems.

## 4 Methods

The methods are available in the Supporting Information.

## Author Contributions

Conceptualization: Hao Wang; Methodology: Hao Wang, Xianfeng Chen, Peiyao Yan, Siqi Liu, Biaobiao Yan, Junhua Kong, Siew Lang Teo, Kai Jin, Jie Zhang, Chaobin He; Software: Hao Wang, Xianfeng Chen; Investigation: Hao Wang, Xianfeng Chen, Biaobiao Yan, Jie Zhang, Chaobin He; Foundation: HCB; Supervision and resources: Chaobin He; Writing – original draft: Hao Wang, XFC; Writing – review & editing: Xianfeng Chen, Junhua Kong, Ping Koy Lam, Chaobin He.

## Funding

The authors acknowledge the National University of Singapore for financial support. We also thank the financial support from Singapore USS LCERII (U2307D4001). The National Natural Science Foundation of China (52571393) also provided funding for this research.

## Conflicts of Interest

The authors declare no conflicts of interest.

## Codes and Data Availability

All data and code supporting the findings of this study are provided in the Supplementary Information and are also available at https://github.com/Wanghao‐1234/ML.git.

## Supporting information




**Supporting File 1**: adma72915‐sup‐0001‐SuppMat.docx.

**Supporting File:2**: adma72915‐sup‐0002‐Video S1.mp4.

**Supporting File:3**: adma72915‐sup‐0003‐Video S2.mp4.

**Supporting File:4**: adma72915‐sup‐0004‐Video S3.mp4.

**Supporting File:5**: adma72915‐sup‐0005‐Video S4.mp4.

**Supporting File:6**: adma72915‐sup‐0006‐Video S5.mp4.

## Data Availability

The data that support the findings of this study are available from the corresponding author upon reasonable request.
