# Supplementary material for: Machine Learning–Assisted Bio‐Interfacial Engineering Resolves Structural–Functional Conflicts in Nanocomposites
Source: Adv Mater. 2026 Mar 25;38(24):e18806. doi: 10.1002/adma.202518806 (PMC13113244; doi:10.1002/adma.202518806)
Supplement: Supplementary file 1 — Supporting File 1: adma72915‐sup‐0001‐SuppMat.docx. [file ADMA-38-e18806-s001.docx]

**Supplementary Information** for

**Machine learning–assisted bio-interfacial engineering resolves structural–functional conflicts in nanocomposites**

Hao Wang^1^, Xianfeng Chen ^2*^, Peiyao Yan ^1^, Siqi Liu ^1^, Biaobiao Yan^1^, Junhua Kong ^2^, Siew Lang Teo ^2^, Kai Jin^3^*, Jie Zhang ^4^*, Ping Koy Lam ^2^, Chaobin He^1,2*^

^1^ Department of Materials Science and Engineering, National University of Singapore, Singapore

^2^ 2 Institute for Materials Research and Engineering(IMRE), Agency for Science,Technology and Research(A*STAR), 2 Fusionopolis Way, 08-03 Innovis, 138634, Singapore

^3^School of Materials Science and Engineering, Ocean University of China, Qingdao, China

^4^Department of Civil and Environmental Engineering, National University of Singapore, 1 Engineering Drive 2,

117576, Singapore

*Corresponding Authors: xianfeng_chen@imre.a-star.edu.sg, jinkai@ouc.edu.cn, jie-cee@nus.edu.sg, msehc@nus.edu.sg

**Supplementary Notes**

**Note 1. Materials and Methods**

**Materials**

Poly(vinyl alcohol) (PVA, Mw ≈ 89,000–98,000), polyethylene glycol (PEG, Mw = 4,000), Malt extract, Peptone, Agar, D-glucose, Yeast extract, NaC_5_H_8_NO_4_, KH_2_PO_4_, K_2_HPO_4_, and MgSO_4_ were purchased from Sigma-Aldrich, Singapore. Graphene nanosheets (Average thickness: 8 nm, Average particle (lateral) size: ~ 5 μm) were sourced from the Graphene Supermarket, United States. Ti_3_AlC_2_ MAX (400 mesh, 98%) powder was provided by Yiyi Technology Co. *Schizophyllum commune* mycelium culture was obtained from a local biological supplier. All reagents were used without further purification.

**Preparation of PEG-intercalated graphene**

Graphene was ultrasonically dispersed in deionized water at a concentration of 2 mg/mL. PEG solution (10 wt%) was added under stirring to facilitate intercalation between graphene sheets. The mixture was stirred for 6 hours.

**Preparation of mycelium growth nutrition**

Solid media: Prepare malt extract agar by dissolving 15 g of agar, 10 g of peptone, and 30 g of malt extract in 1 L of deionized water.

Liquid culture medium: Create the liquid culture medium by dissolving the following components in 1 L of deionized water, adjusting the pH to 5.5: D-glucose: 15 g, Peptone: 2.5 g, Yeast extract: 3 g, KH_2_PO_4_: 1 g, K_2_HPO_4_: 0.2 g, MgSO_4_: 0.5 g.

Graphene-PVA liquid culture medium: By dissolving the following components in 250 ml of water: D-glucose: 3 g, KH_2_PO_4_: 0.5 g, K_2_HPO_4_: 0.04 g, MgSO_4_: 0.1 g, PVA: 1 g.

**Fabrication of mycelium assembled graphene composites (MGCs)**
Heat a PVA aqueous solution (0% to 10 wt%) to 90°C and maintain for 1 hour. Pour the graphene intercalation solution into the PVA solution at a concentration of 0% to 20% by weight. Disperse with ultrasonic stirring for 30 minutes and cool for 4 hours. Then, inoculate the pre-cultured *Schizophyllum commune* mycelial seed solution. The mixture was cast into sterile molds and incubated at 25 °C with 80% humidity for 5–10 days to allow for fungal network formation.

**Fabrication of PVA mixed PEG-graphene composites**

To prepare the reference PVA mixed graphene composites, a PVA solution was prepared by dissolving PVA powder (Mw ≈ 89,000–98,000, 99+% hydrolyzed) in deionized water under magnetic stirring at 90 °C. The PEG-intercalated graphene dispersion was added dropwise into the PVA solution under vigorous stirring. The mixture is stirred for 4 h and subsequently cast into Petri dishes for solvent evaporation at 50 °C for 48 h, forming homogeneous hybrid films.

**Fabrication of Mycelium assembled MXene composites (MMCs)**

Highly concentrated MXene dispersions were synthesized following a previously reported method [1]. PEG-intercalated MXene was prepared using the same intercalation strategy as that employed for graphene. The fabrication of mycelium assembled MXene composites (MCCs) follows the same procedure as that used for MGCs. After growth, the resulting films are dried at 50 °C for 24 h prior to further characterization. To prepare the reference PVA mixed MXene composites, the same method was used as PVA mixed graphene composites.

**Morphology analysis**

Digital images of the samples were captured using a Sony camera. The mycelium samples were visualized through optical imaging using a Zeiss Axio Scope A1 microscope manufactured by Zeiss. Scanning Electron Microscope (SEM): Following the drying process at room temperature, MGCs were subjected to imaging using a field emission scanning electron microscope (Zeiss Sigma 300) operating at an accelerating voltage of 5 kV. Prior to imaging, a gold coating with a thickness of 5 nm was applied to the samples. ZEISS Xradia 610 Versa 3D X-ray Microscopy was used to 3D san MGCs samples. The morphology of the samples was characterized using a Helios 5 dual-beam scanning electron microscope (SEM). Transmission Electron Microscopy (TEM) analysis was conducted using FEI Helios NanoLab 450S FIB.

**Mechanical properties test**

The mechanical properties of the material were characterized through tensile testing conducted in accordance with ISO 527 type 5A standards. The testing was performed using an Instron machine (Instron 5900, USA) at room temperature with a testing speed of 0.2 mm/min. Specimens, fixed with clamps positioned 10 mm from both ends, were subjected to the testing protocol. Samples of MGCs cultivated with varying graphene contentswere employed for tensile testing, and each test was replicated three times for every individual sample. Tensile strength (MPa) was determined by dividing the maximum load (N) by the sample's cross-sectional area (mm^2^). Density (g/cm³) was computed by dividing the sample's weight by its volume.

**In-situ tensile SEM**
The in-situ tensile experiment was performed using a Deben Microtest module mounted inside the SEM chamber. Rectangular MGCs specimens were cut into narrow strips and fixed onto miniature tensile grips. The test was conducted under high vacuum with an electron beam accelerating voltage of 10 kV. The loading is applied in displacement-control mode at a constant rate of 10 μm/s. Real-time secondary electron images are continuously recorded during deformation to track crack-tip evolution.

**Laser cutter**

The levitated plates were cut into desired dimensions using a micro laser cutting system IDI Laser UV-17-C. To determine the density of the material, the micro lasre cutter was used to cut the plate into different size and use a precision weighing scale to obtain their mass.

**Levitation height measurements**

The levitation height was measured using a precision laser distance sensor (Keyence IL-S065) with a resolution of 1µm. To obtain the levitation height, we first positioned the laser spot on the top surface of the magnets and recorded the distance $D1$. We then moved the laser spot to the top surface of the composite plate and recorded the distance $D2$, as shown in **Figure S1**. The levitation heigh $H$ then can be obtained by $H = D1-D2-t$, where $t$ is the thickness of the sample, which is measured using a digital caliper. To reduce the influence of surface curvature and roughness, all samples were gently polished before levitation testing. We repeated the above measurements five times at different locations of the composite plate to obtain an average value of the levitation height.


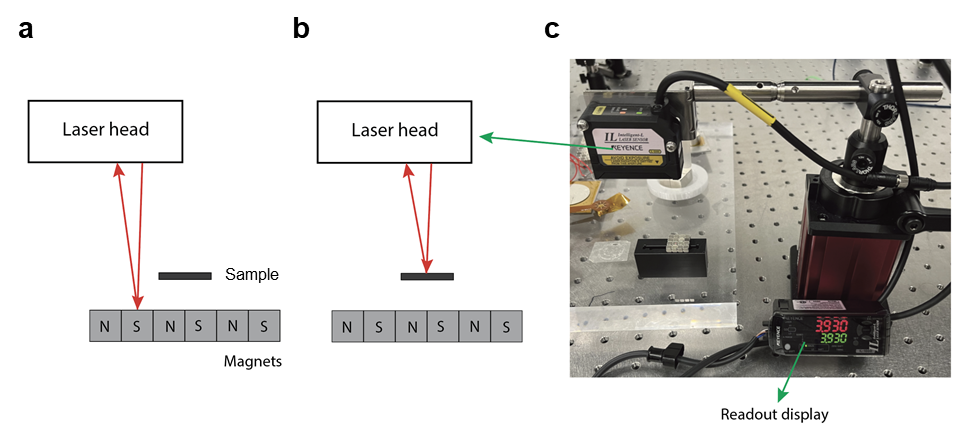


**Figure S1** **Measurement of the levitation height with a laser distance sensor.** The distance sensor laser is positioned at the magnets (**a**) and the sample (**b**), respectively. (**c**) An image of the experimental setup.

**Diamagnetic levitation micro robot**

The laser-driven levitation micro robot operates with a laser power of 500mW at a wavelength of 405 nm. The magnetic field used in the system is generated by an array of N52 neodymium permanent magnets with dimensions of 3 x 3 x 3 mm^3^. The interaction between the magnetic field and the diamagnetic properties of the MGCs results in levitation and controlled movement of the micro robot in response to the laser-induced thermal effects.

Rotational behavior in a circular magnetic field was induced by wind generated from a dropper. The magnetic array was composed of annular magnets (outer radius 10 mm, inner radius 3 mm, thickness 6 mm) and cylindrical magnets (radius 3 mm, thickness 6 mm).

**Self-healing and regenerative test**

In the self-healing test, two isolated MGCs were placed in solid nutrient cultures. Following 5 days of growth, the wounds were initially sutured, and the specimens were immersed in a liquid nutrient solution containing 20% graphene and 10% PVA. For the self-regenerative test, a small piece of MGCs, which had been air-dried at room temperature for 3 months, was introduced into a solid medium for growth. After a 5-day incubation period, the sample was transferred into a liquid nutrient solution containing 20% graphene and 10% PVA.

**Electromagnetic Interference (EMI) Shielding Measurements**

EMI shielding effectiveness (SE) was measured using a vector network analyzer (Keysight E5071C) in the X-band and Ku-band frequency ranges (8–18 GHz) following ASTM D4935-10 standard. Each film sample was cut into 22 mm × 10 mm rectangular strips and mounted between waveguide fixtures. The thickness of the sample is 0.65 mm (± 0.05 mm). Total shielding effectiveness ($\mathrm{SE}_{T}$) is calculated from scattering parameters as:

$$\mathrm{SE}_{T}=-10\log_{10} (\frac{P_{T}}{P_{I}})$$

where $P_{T}$ and $P_{I}$ represent transmitted and incident power, respectively. All measurements were averaged over three samples to ensure reproducibility.

**Note 2. Growth-mediated assembly of mycelium–graphene composites (MGCs)**

We employ optical microscopy (top) and 3D surface profilometry (bottom) to characterize the surface morphology and mesoscale roughness of the *Schizophyllum commune* mycelium (**Figure S2**). The optical image reveals a continuous fibrous network of randomly oriented hyphae, forming a nonwoven scaffold that mimics natural extracellular matrices. Such topological heterogeneity enhances the overall surface area, facilitates mechanical interlocking with PVA and graphene components.


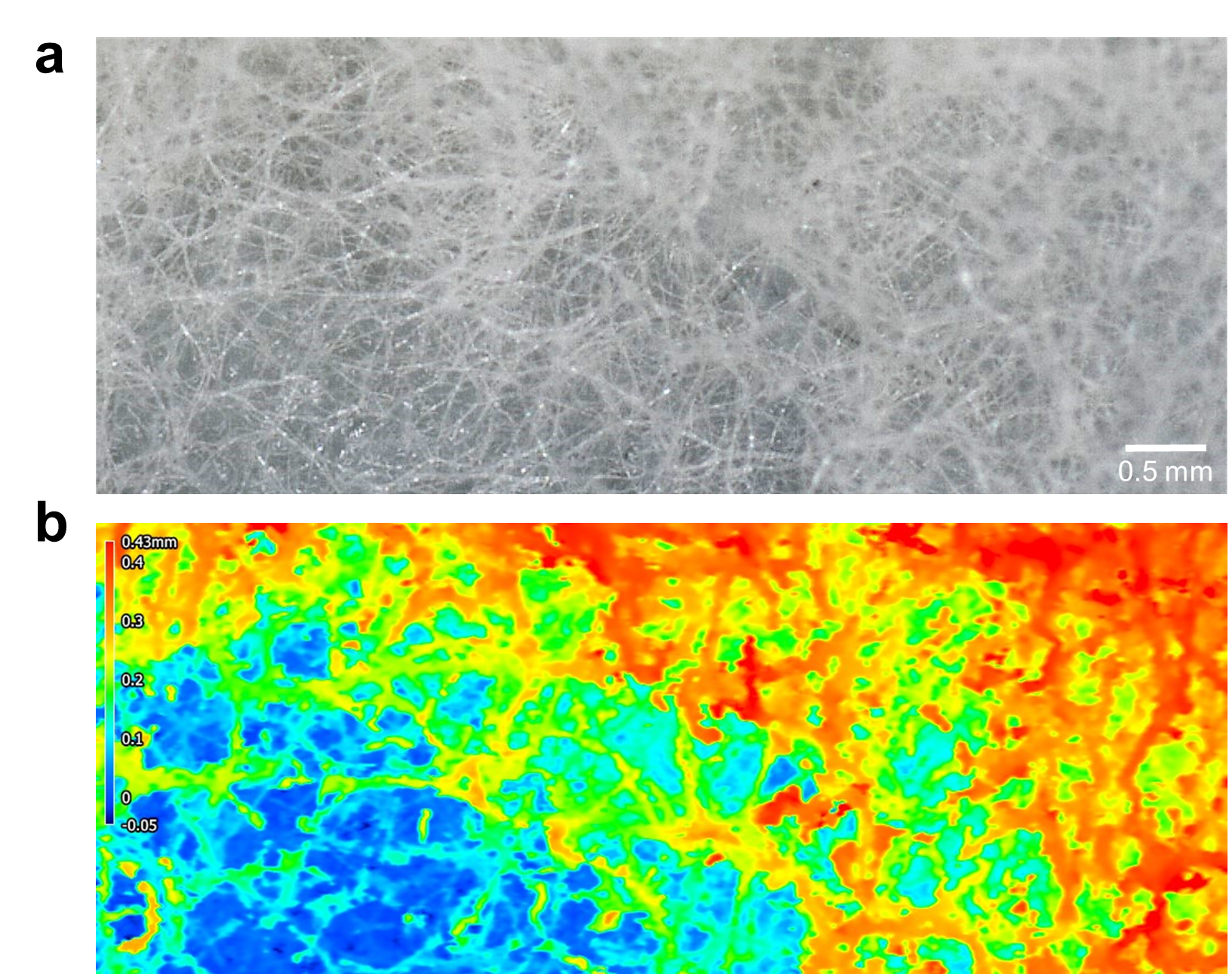


**Figure S2 Surface morphology and topographic structure of the *Schizophyllum commune* mycelium.** **a**. Optical microscopy image showing the entangled hyphal network of the mycelium-assembled biofilm. The randomly oriented filaments form a continuous fibrous scaffold with micron-scale porosity and interconnectivity. **b**. Three-dimensional surface profilometry map revealing topographic variations across the composite surface. The height distribution ranges from ~0.05 mm to ~0.43 mm, indicating multiscale roughness.





**Figure S3 TEM image of graphene nanosheets.**

The FTIR spectra reveal the successful intercalation of PEG into the graphene, evidenced by the emergence of characteristic peaks at ~3400 cm^-1^ (O–H stretching), ~2900 cm^-1^ (C–H stretching), and ~1100 cm^-1^ (C–O–C vibrations) in the PEG-intercalated graphene nanosheet (PEG-graphene) sample. These features are absent in pristine graphene, indicating the formation of interfacial interactions such as hydrogen bonding and van der Waals forces.


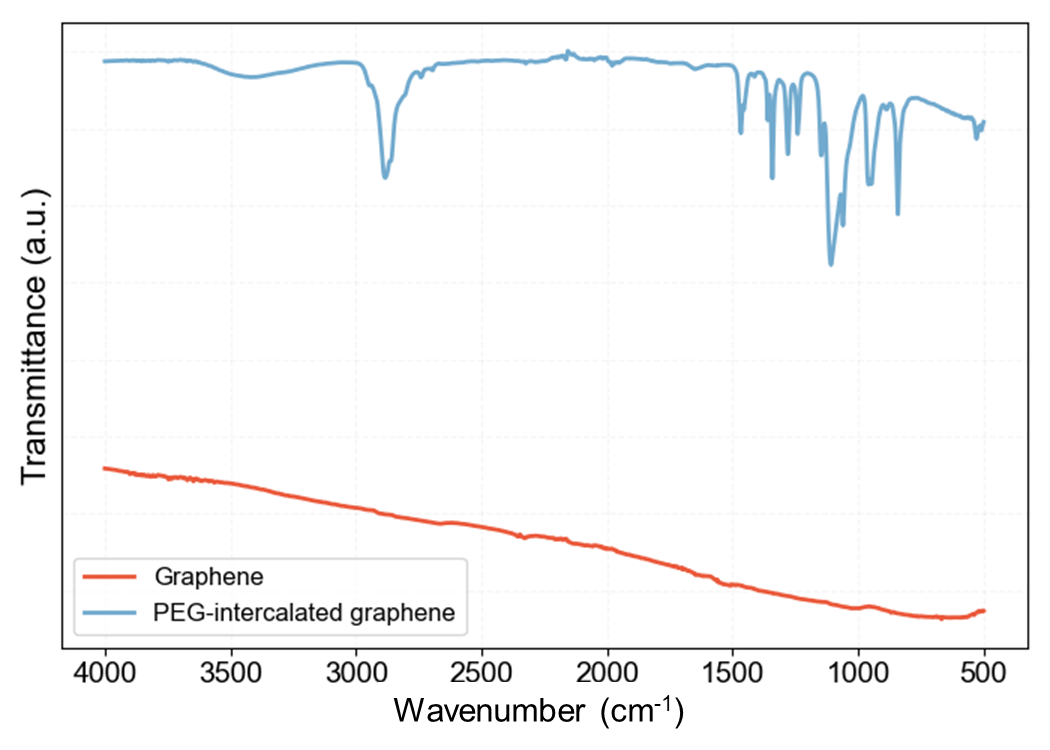


**Figure S4 FTIR spectra of pristine graphene and PEG-intercalated graphene.** Fourier-transform infrared (FTIR) spectra reveal the emergence of characteristic absorption bands in the PEG-intercalated graphene sample, including ~3400 cm^-1^ (O–H stretching), ~2900 cm^-1^ (C–H stretching), and ~1100 cm^-1^ (C–O–C vibrations), which are absent in pristine graphene.

To validate the formation of anchored and interlocked interfaces during bio-assisted assembly, optical microscopy is employed to probe the spatial relationship between fungal hyphae and graphene nanosheets (**Figure S5a**). In mycelium–graphene composites (MGCs), graphene nanosheets are observed colocalizing with and adhering to hyphal surfaces, consistent with the emergence of a pinned-and-bridged interfacial topology. As a control, pure mycelium cultured without graphene develops a characteristic web-like hyphal network but lacks nanosheet attachment along the filaments. This microscale evidence of nanosheet–hyphae interaction is corroborated at the macroscopic level: after incubation, MGCs spontaneously form freestanding bulk composites (**Figure S5b**), reflecting successful interface assembly in which nanosheet pinning and multiscale entanglement establish a spatially interconnected architecture. These observations provide direct morphological evidence for the biologically guided interfacial construction mechanism illustrated in **Figure 1**.


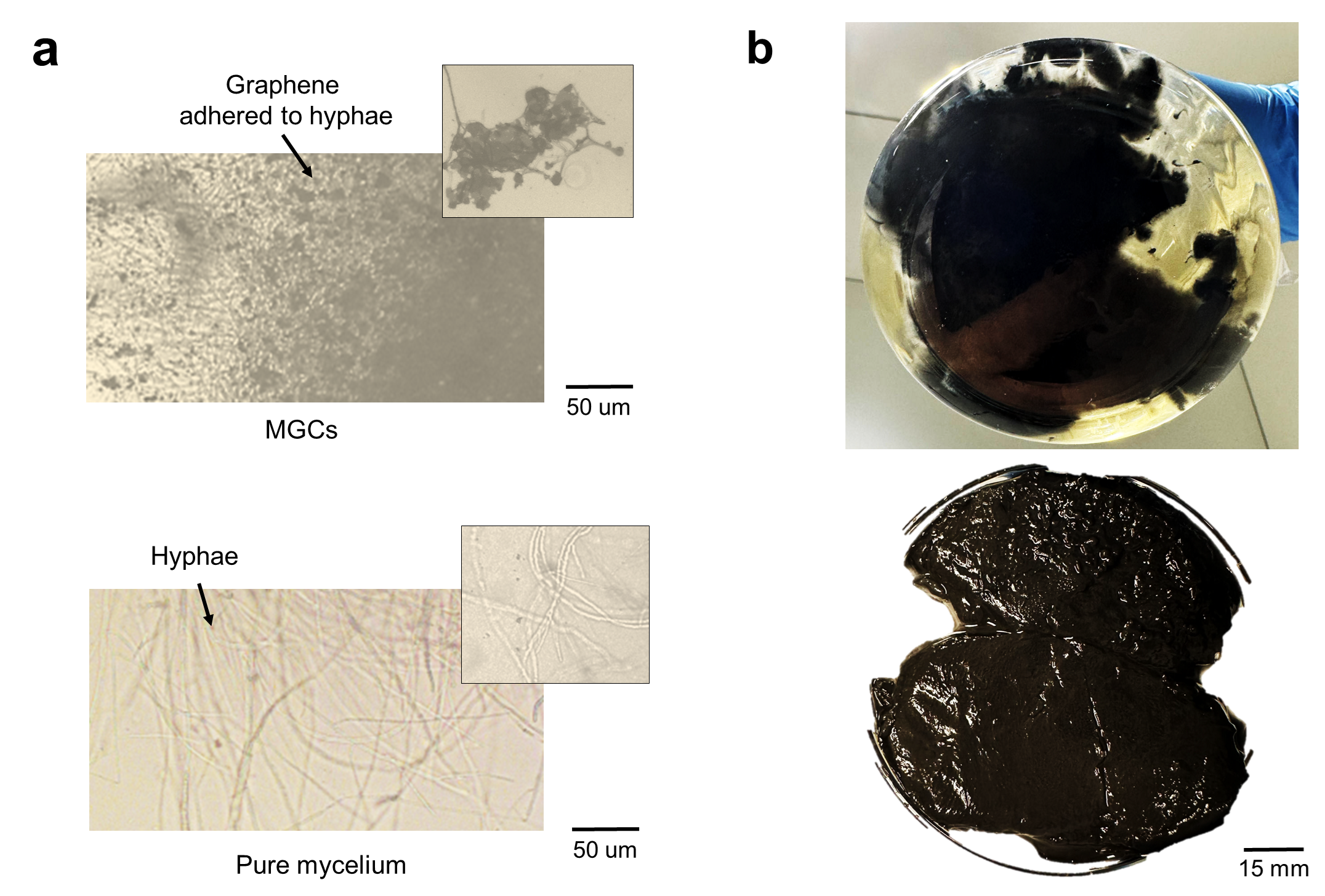


**Figure S5 Experimental validation of bio-directed interfacial reconfiguration.** **a**. Optical microscopy images of *Schizophyllum commune* cultured for 5 days in a PEG–graphene nutrient solution (top) and in a control solution without graphene (bottom). In the composite system, graphene nanosheets adhere to and align along fungal hyphae, establishing a pinned-and-bridged interface through growth-mediated assembly. Insets show magnified views highlighting nanosheet–hypha entanglement. **b**. Photographs of mycelium–graphene composites (MGCs) after 8 days of incubation, demonstrating spontaneous consolidation into freestanding bulk materials. System composition: 10 wt% PVA, 11 wt% graphene, total volume 250 mL.

**Note 3. Machine learning**

**Note 3.1 Gaussian Process (GP) modeling and validation.**
In this study, we employ a Design of Experiments (DoE) approach to systematically select 30 material samples, each representing a unique combination of composition and processing parameters. These samples are experimentally tested to obtain their mechanical properties (strength and toughness) and functional properties (diamagnetic levitation height). These three properties are critical for evaluating the performance of our composites.

To predict the key mechanical and functional properties of the bio-composite system including tensile strength ($f_{1}\left( x \right)$, MPa), toughness ($f_{2}\left( x \right)$, MJ/m^3^), and levitation height ($f_{3}\left( x \right)$, mm), we develop a multi-target Gaussian Process Regression (GPR) framework. The input variables include PEG-intercalated graphene (PEG-G) content ($x_{1}$, wt%​), polyvinyl alcohol (PVA) content ($x_{2}$, wt%​), and mycelium growth time ($x_{3}$, Days​), forming the feature vector:

$\boldsymbol{x}=\left[ x_{1} x_{2} x_{3} x_{2}^{2} x_{1}\cdot x_{2} \right]^{T}$ (1)

where $x_{2}^{2}$​ and $x_{1}\cdot x_{2}$​ are engineered polynomial and interaction terms introduced to capture nonlinear coupling effects.

Each output $f_{(i)}\left( \boldsymbol{x} \right)$ is modeled by an independent GPR:

$f_{i}\left( \boldsymbol{x} \right)\sim\text{GP }\left( \mu_{i}\left( \boldsymbol{x} \right),k_{i}\left( \boldsymbol{x},\boldsymbol{x}^{'} \right) \right), i=1,2,3$ (2)

where $\mu_{i}\left( \boldsymbol{x} \right)$ denotes the predictive mean, and $k_{i}\left( \mathbf{x},\mathbf{x}^{'} \right)$ is a composite kernel defined as:

$k_{i}\left( \boldsymbol{x}, \boldsymbol{x}^{'} \right)=k_{Matern}\left( \boldsymbol{x,}\boldsymbol{x}^{\boldsymbol{'}} \right)+ k_{RBF}\left( \boldsymbol{x,}\boldsymbol{x}^{\boldsymbol{'}} \right)+ k_{WhiteNoise}\left( \boldsymbol{x,}\boldsymbol{x}^{\boldsymbol{'}} \right)$ (3)

where

$k_{Matern}\left( \boldsymbol{x}, \boldsymbol{x}^{'} \right)= \sigma_{f}^{2}\left( 1 + \frac{\sqrt{5}r}{l}+\frac{5r^{2}}{3l^{2}} \right)exp\left( -\frac{\sqrt{5}}{l}r \right)$ (4)

$k_{RBF}\left( \boldsymbol{x}, \boldsymbol{x}^{'} \right)= \sigma_{f}^{2}exp\left( -\frac{\left| \left| \boldsymbol{x} - \boldsymbol{x}^{'} \right| \right|^{2}}{{2l}^{2}} \right)$ (5)

$k_{WhiteNoise}\left( \boldsymbol{x}, \boldsymbol{x}^{'} \right)=\sigma_{n}^{2}\cdot\delta\left( \boldsymbol{x}, \boldsymbol{x}^{'} \right)$ (6)

with $r=\parallel\boldsymbol{x}-\boldsymbol{x}^{'}\parallel$, and hyperparameters $l$, $\sigma_{f}$​, and $\sigma_{n}$ optimized by maximizing the marginal likelihood via 5-fold cross-validation. The Matérn kernel ($\nu=2.5$) is selected for its capability to model non-smooth functions, while the RBF kernel ensures local continuity, and the White kernel accounts for experimental noise.

The data ($n=30$) are split into 25 training samples and 5 test samples, ensuring identical splits across the three targets. All input and output variables are standardized via z-score normalization. For the Toughness target, we apply a logarithmic transform:

$\tilde{f}^{2}=\log\left( 1+f^{2} \right)$ (7)

before training, and using the inverse transform $exp(\cdot)-1$ during post-processing to recover predictions on the original scale.

For each target, we evaluate the predictive performance using the coefficient of determination $R^{2}$ and the root mean squared error (RMSE):

$R^{2}=1-\frac{{\sum_{j=1}^{n_{test}} (f_{j-}\hat{f}_{j})}^{2}}{{\sum_{j=1}^{n_{test}} (f_{j-}\bar{f}_{j})}^{2}}$ (8)

$RMSE=\sqrt{\frac{1}{n_{test}}\sum_{j=1}^{n_{test}} {(f_{j-}\hat{f}_{j})}^{2}}$ (9)

where $\hat{f}_{j}$ is the predicted mean from the GPR model, and the standard deviation $\sigma_{j}$ provided an uncertainty estimate.

The predictive performance of the Gaussian Process Regression (GPR) models is summarized in **Figure S6** (true versus predicted plots for the test set). For tensile strength, the model achieves an R^2^ of 0.89, with prediction errors confined within ±6 MPa and calibrated uncertainties in the range of 2.2–5.1 MPa. The toughness model attains an R^2^ of 0.70, successfully distinguishing high and low toughness regimes with uncertainties below 0.5 MJ/m^3^. The levitation model performs best, yielding an R^2^ of 0.96 and highly consistent predictions across the test samples.

These results confirm that the GPR models provide accurate and well-calibrated predictions for all three target properties. Although toughness exhibits greater variability due to its sensitivity to microstructural heterogeneity, the model captures the overall trend and preserves the relative ranking of samples, while assigning appropriate uncertainty bounds. These findings demonstrate that GPR effectively learns the underlying structure–property relationships in biologically assembled composites and establishes a reliable surrogate model for guiding subsequent multi-objective optimization.


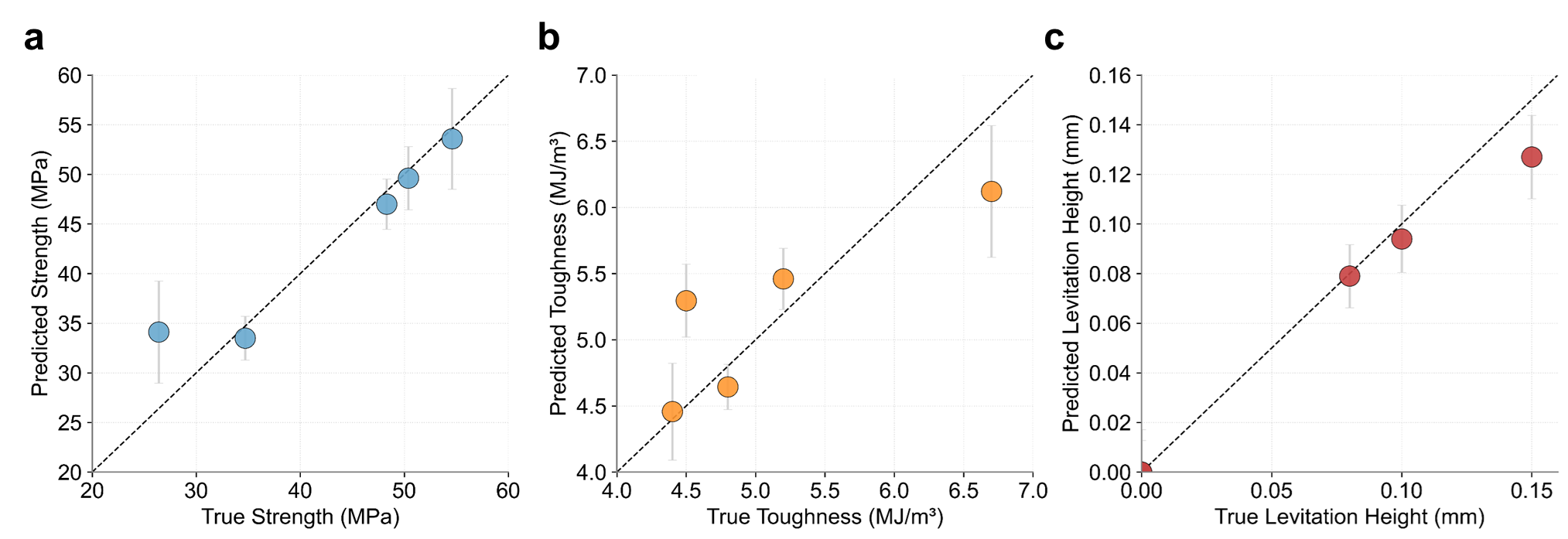


**Figure S6** **GP model predictions versus experimental measurements.** **a**. Strength (MPa), **b**. Toughness (MJ/m^3^), and **c**. Levitation height (mm). Each subplot compares experimentally measured values (x-axis) with GP model predictions (y-axis). Error bars denote the standard deviation of the predictive distribution, representing model uncertainty. The dashed diagonal indicates perfect prediction. The results show that GP models accurately capture structure–property relationships and provide reliable uncertainty quantification across multiple mechanical and functional properties.

**Note 3.2 Pareto Set Learning (PSL)**

To investigate the trade-offs among tensile strength, toughness, and levitation height in biologically assembled composites, we employ GP models as efficient surrogates to predict material properties and enable multi-objective optimization. The optimization task is formulated through scalarization, whereby the original multi-objective problem is transformed into a single-objective one. Among various scalarization strategies, we adopt the Tchebycheff (TCH) method owing to its theoretical rigor and broad applicability [2]. The TCH approach is well suited for non-convex Pareto fronts and allows flexible adjustment of performance priorities via weight parameters. As a result, it yields a diverse and comprehensive set of Pareto-optimal solutions that provide actionable guidance for materials design.

The TCH scalarization is defined as:

$\underset{\boldsymbol{x}\in\boldsymbol{X}}{\mathrm{Min}} g_{\text{tch}}\left( \boldsymbol{x} | \lambda\right)=\min_{x\in X} \max_{1\leq i\leq m} \left\{ \lambda_{i}\left( f_{i}\left( \boldsymbol{x} \right)-\left( z_{i}^{*}-\epsilon\right) \right) \right\}$ (10)

where $z^{*}=\left( z_{1}^{*},\ldots,z_{m}^{*} \right)$ represents the ideal vector for the objective vector $f\left( \boldsymbol{x} \right)$ (i.e., the lower bound for a minimization problem), $\epsilon> 0$ is a small positive scalar, and $u_{i}=\left( z_{i}^{*}-\epsilon\right)$ denotes the unachievable utopia value for the $i$-th objective $f_{i}\left( \boldsymbol{x} \right)$.

All Pareto solutions $\boldsymbol{x}\in\boldsymbol{M}_{\text{ps}}$ can be identified by solving the TCH scalarized subproblem with specific trade-off preferences $\lambda$. Let $\boldsymbol{M}_{\text{tch}}$ denote the solution set for problem (10) across all valid preferences $\Lambda$. Thus, $\boldsymbol{M}_{\text{ps}}\subseteq\boldsymbol{M}_{\text{weak}}=\boldsymbol{M}_{\text{tch}}$. Weakly Pareto optimal solutions, which are dominated (but not strictly dominated) by some Pareto solutions, are undesirable for decision-making. To eliminate these, we use the augmented Tchebycheff (TCH-aug) approach, formulated as:

$g_{\text{tch}\text{\_}\text{aug}}\left( \boldsymbol{x} | \lambda\right)=\max_{1\leq i\leq m} \left\{ \lambda_{i}\left( f_{i}\left( \boldsymbol{x} \right)-\left( z_{i}^{*}-\epsilon\right) \right) \right\}+\rho\sum_{i=1}^{m} \lambda_{i}f_{i}\left( \boldsymbol{x} \right),\quad\forall\lambda\in\Lambda$ (11)

where $\rho$ is a small positive scalar dependent on the problem and solution location. This ensures weakly dominated solutions have larger scalarized values, excluding them during optimization. We set $\rho= 0.001$, dynamically update $z_{i}^{*}$ as the current best value for each objective, and let $\epsilon=0.1\left| z^{*} \right|$.

Traditional methods solve the scalarization problem (11) with a finite set of preferences $\lambda$ sequentially or collaboratively, but these are limited in exploring the entire Pareto front efficiently. Therefore, in this work we propose to construct a set model that maps preferences to their corresponding solutions:

$\boldsymbol{x}\left( \lambda\right)=h_{\boldsymbol{\theta}}\left( \lambda\right)$ (12)

where $\lambda$ is any valid preference in $\Lambda= \{\lambda\in R_{+}^{3} | \sum_{i=1}^{3} \lambda_{i}=1\}$ \), $\boldsymbol{x}\left( \lambda\right)\in X$ is its corresponding Pareto solution, and $h_{\boldsymbol{\theta}}\left( \lambda\right)$ is the Pareto set model parameterized by $\boldsymbol{\theta}$. The input preference $\lambda$ has $\left( m-1 \right)$ degrees of freedom, and the output solution set $\boldsymbol{M}_{psl} = \{\boldsymbol{x} = h_{\boldsymbol{\theta}}(\lambda) | \lambda\in\Lambda\}$ lies on an $\left( m-1 \right)$-dimensional manifold in $\boldsymbol{X}\in R^{n}$. In other words, the set model maps the $\left( m-1 \right)$-dimensional preference simplex $\Lambda$ to the $\left( m-1 \right)$-dimensional solution set $\boldsymbol{M}_{psl}$, which has a complex structure.

Our goal is to find the optimal parameters $\theta^{*}$ such that the generated set $\boldsymbol{M}_{psl}$ matches the solution set for the augmented Tchebycheff scalarization $\boldsymbol{M}_{psl} = \{\boldsymbol{x}^{*}(\lambda) | \lambda\in\Lambda\}$, where:

$\boldsymbol{x}^{*}\left( \lambda\right)= h_{\boldsymbol{\theta}^{*}} \left( \lambda\right)= \mathrm{argmin}_{\boldsymbol{x}\in X} g_{\text{tch}\text{\_}\text{aug}}(\boldsymbol{x}|\lambda), \quad\forall\lambda\in\Lambda$ (13)

This enables exploration of the approximate Pareto set/front by adjusting preferences. We implement a Multi-Layer Perceptron (MLP) neural network as the set model, facilitating efficient navigation of trade-offs among strength, toughness, and levitation height.

Given the high experimental cost of evaluating $f\left( \boldsymbol{x}\left( \lambda\right) \right)=f\left( h_{\boldsymbol{\theta}}\left( \lambda\right) \right)$, we adopt a GPR-based approach to learn $h_{\boldsymbol{\theta}}\left( \lambda\right)$. Given a learned Pareto set $\boldsymbol{M}_{\text{psl}}$, the GPR models provide both the predicted values $\hat{\mu}\left( \boldsymbol{M}_{\text{psl}} \right)=\{\hat{\mu}\left( \boldsymbol{x} \right)\mid\boldsymbol{x}\in\boldsymbol{M}_{\text{psl}}\}$ and the uncertainties $\hat{\sigma}^{2}\left( \boldsymbol{M}_{\text{psl}} \right)=\{\hat{\sigma}^{2}\left( \boldsymbol{x} \right)\mid\boldsymbol{x}\in\boldsymbol{M}_{\text{psl}}\}$ for the entire approximate Pareto set.

To determine the optimal parameter $\boldsymbol{\theta}^{*}$ for the Pareto set model $h_{\boldsymbol{\theta}}\left( \lambda\right)$, we propose an efficient algorithm. Since the optimal solution set $\boldsymbol{M}_{\text{tch}\text{\_}\text{aug}}$ for the augmented Tchebycheff scalarization (11) is unknown, we optimize all solutions generated by our model $\boldsymbol{x}\left( \lambda\right)=h_{\boldsymbol{\theta}}\left( \lambda\right)$ with respect to their corresponding augmented Tchebycheff scalarization subproblems for all valid preferences:

$\boldsymbol{\theta}^{*} =\mathrm{argmin}_{\boldsymbol{\theta}}E_{\lambda\sim\Lambda} [g_{\mathrm{tch}\_\mathrm{aug}}(\boldsymbol{x} = h_{\boldsymbol{\theta}}(\lambda) \mid\lambda)]$ (14)

If the model is perfectly learned, the approximate Pareto set $\boldsymbol{M}_{\text{psl}}=\{\boldsymbol{x}=h_{\boldsymbol{\theta}}\left( \lambda\right) | \lambda\in\Lambda\}$ should coincide with $\boldsymbol{M}_{\text{tch}\text{\_}\text{aug}}$. However, directly optimizing (14) is challenging due to the expectation over an infinite set of preferences $\left| \Lambda\right|=\infty$. To address this, we employ Monte Carlo sampling and gradient descent to iteratively learn the model using the surrogate model:

$\boldsymbol{\theta}_{t+1}= \boldsymbol{\theta}_{t}-\eta\sum_{k=1}^{K} \nabla_{\boldsymbol{\theta}}\hat{g}_{\mathrm{tch}\_\mathrm{aug}}(\boldsymbol{x}=h_{\boldsymbol{\theta}}(\lambda_{k}) \mid\lambda_{k})$ (15)

where $K = 10$ different valid preferences $\{\lambda_{1},\ldots,\lambda_{K}\}\sim\Lambda$ are randomly sampled at each iteration. Here, $\hat{g}_{\mathrm{tch}\_\mathrm{aug}}$ represents the augmented Tchebycheff scalarization with predicted objective values:

$\hat{g}_{\text{tch}\text{\_}\text{aug}}\left( \boldsymbol{x} | \lambda\right)=\max_{1\leq i\leq m} \left\{ \lambda_{i}\left( \hat{f}_{i}\left( \boldsymbol{x} \right)-\left( z_{i}^{*}-\epsilon\right) \right) \right\}+\rho\sum_{i=1}^{m} \lambda_{i}\hat{f}_{i}\left( \boldsymbol{x} \right)$ (16)

The posterior mean of the GP models serves as the surrogate value to derive the approximate Pareto front, enabling informed trade-offs among strength, toughness, and levitation height in biomimetic composite design.

We perform 1000 iterations of optimization using the Pareto Set Learning (PSL) framework to train the set model $h_{\boldsymbol{\theta}}\left( \lambda\right)$. Upon convergence, approximate Pareto-optimal solutions are directly generated by mapping randomly sampled preference vectors to design variables. To systematically evaluate the performance and coverage of the learned PSL model, we sample 1000 uniformly distributed preference vectors ($\lambda\in\Lambda$) and compute the associated Pareto solutions.

The predicted performance distributions derived from the initial experimental dataset are summarized in **Figure S7**. In the compositional space (**Figure S7a**), the learned λ→x mapping reveals structured clusters rather than random dispersion, suggesting that PSL identifies physically meaningful regions across PVA content, graphene ratio, and mycelium growth duration. In the predicted performance domain (**Figure S7b**), the PSL solutions form a continuous trade-off surface among strength, toughness, and levitation height. The smooth variation of levitation height across the Pareto surface indicates that the model interpolates preference vectors into coherent, physically consistent solutions.

**Figure S7c** further illustrates the distribution of PSL predictions in the two-dimensional performance space of tensile strength versus toughness, with levitation height encoded by color and marginal density plots showing projection distributions. The solutions predominantly occupy the range of 40–55 MPa in strength and 4.5–6.0 MJ/m^3^ in toughness, with levitation height values concentrated below 0.14 mm. The marginal density curves confirm that strength is strongly peaked near 50 MPa, whereas toughness clusters tightly around 5.5 MJ/m^3^. These results demonstrate the utility of PSL in mapping complex multi-objective landscapes, revealing non-trivial trade-offs and performance envelopes that cannot be accessed through conventional design strategies. This demonstrates the critical role of data-driven optimization in tailoring composite properties for targeted applications and advancing rational materials design.


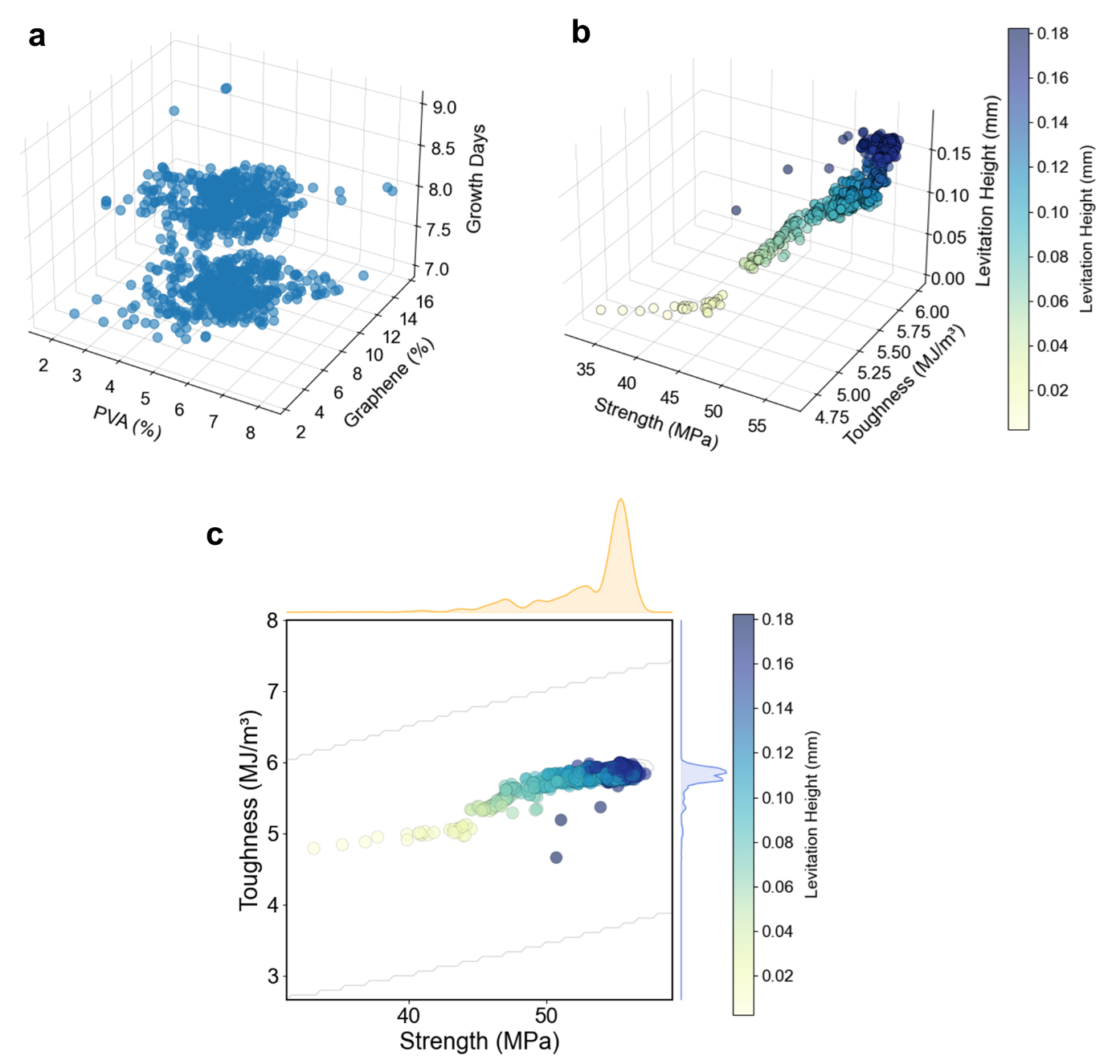


**Figure S7 Predicted performance distribution of PSL solutions based on the initial experimental dataset.** **a**. Distribution of 1000 candidate formulations in the compositional–process space (PVA content, graphene concentration, and mycelium growth duration), showing broad coverage through the λ→x mapping. **b**. Predicted performance of the same candidates in terms of tensile strength, toughness, and levitation height, forming a continuous multi-objective Pareto front. **c**. Two-dimensional performance space of tensile strength versus toughness, with levitation height encoded by color and marginal density plots showing projection distributions. Most solutions fall within moderate ranges of strength (40–55 MPa) and toughness (4.5–6.0 MJ/m^3^), while levitation height remains largely below 0.14 mm. Marginal density curves indicate that strength is strongly peaked near 50 MPa and toughness values cluster around 5.5 MJ/m^3^. Kernel density estimation is used for computing the marginal distributions.

**Note 3.3 Active Learning (AL)**

To further enhance the efficiency and performance of the optimization process, we integrate Active Learning (AL) with Bayesian Optimization (BO) to systematically explore the composite material design space. BO provides a robust probabilistic framework for optimizing expensive, black-box objectives by balancing exploration of uncertain regions and exploitation of areas with high predicted performance via a surrogate model [3, 4]. AL complements this by prioritizing the selection of the most informative data points, thereby significantly reducing the number of required experimental evaluations without compromising optimization fidelity.

We implement a customized AL strategy to direct the sequential selection of candidate formulations. The core principle is to leverage the predictive uncertainty from GP surrogate models to identify regions that are either poorly characterized or hold potential for substantial performance gains. To formalize this trade-off, we employ an acquisition function based on the Lower Confidence Bound (LCB) criterion, which enables a tunable balance between exploration and exploitation. For minimization tasks, the LCB is defined as:

$\hat{f}\left( \boldsymbol{x} \right)= \hat{\mu}\left( \boldsymbol{x} \right)- \beta\cdot\hat{\sigma}\left( \boldsymbol{x} \right)$ (17)

where $\hat{\mu}\left( \boldsymbol{x} \right)$ and $\hat{\sigma}\left( \boldsymbol{x} \right)$ are the predicted mean and standard deviation from the GP model, respectively, and $\beta$ is a tunable hyperparameter that controls the trade-off between exploration and exploitation. In this work, we empirically adopt a decaying exploration parameter schedule for the LCB acquisition function, with β initialized at 0.2 and progressively reduced to 0.25, 0.2, and 0.1 across successive AL iterations. This adaptive strategy gradually shifts the balance from exploration toward exploitation, allowing the model to focus more on refining high-potential regions of the design space as predictive confidence improves. The LCB formulation prioritizes regions with either high uncertainty or promising predicted performance, thereby enabling efficient and targeted discovery of optimal material formulations and processing.

The AL strategy comprises two key components: batch sampling from the approximate Pareto set and batch selection based on hypervolume improvement (HVI). Leveraging the flexibility of the PSL model, we can efficiently generate a large number of candidate solutions in parallel, making it well-suited for batched acquisition. When available, decision-maker preferences can be directly incorporated to guide the generation of solutions. In the absence of such prior knowledge, we uniformly sample$P=1,000$ valid preference vectors $\{\lambda^{p}{\}}_{p=1}^{P}$ and compute the corresponding candidate solutions $\boldsymbol{X}=\{\boldsymbol{x}\left( \lambda^{p} \right){\}}_{p=1}^{P}$​ from the learned approximate Pareto set $\boldsymbol{M}_{\text{psl}}$​.

At each iteration of multi-objective Bayesian optimization (MOBO), a small batch of candidate solutions $\boldsymbol{X}_{B}=\{\boldsymbol{x}^{b}{\}}_{b=1}^{B}$ ($B=5$ in this work) is selected from the generated pool $\boldsymbol{X}$ for experimental evaluation. To ensure meaningful improvement of the current Pareto front, we employ hypervolume improvement (HVI) as the selection criterion. The hypervolume metric $\text{HV}\left( \boldsymbol{y} \right)$ quantifies the volume of the region $S\subset R^{m}$ in the objective space that is dominated by a set of solutions $\boldsymbol{y}$ in the objective space:

$S=\{r\in R^{m}\mid\exists y\in\boldsymbol{y}\text{ such that }y\prec r\prec r^{*}\}$ (18)

where the reference point $r^{*}$is chosen such that it is dominated by all solutions $y\in\boldsymbol{y}$ in the objective space. The hypervolume improvement (HVI) of a candidate batch $\boldsymbol{X}_{B}$​ with respect to the previously evaluated set $\{\boldsymbol{X}_{t-1},\boldsymbol{y}_{t-1}\}$ is defined as:

$\text{HVI}\left( \hat{f}\left( \boldsymbol{X}_{B} \right) \right)=\text{HV}\left( \boldsymbol{y}_{t-1}\cup\hat{f}\left( \boldsymbol{X}_{B} \right) \right)-\text{HV}\left( \boldsymbol{y}_{t-1} \right)$ (19)

where $\hat{f}\left( \boldsymbol{X}_{B} \right)$ denotes the predicted objective values of the batch $\boldsymbol{X}_{B}$​ given by the surrogate model. We primarily adopt the LCB (14) as the surrogate acquisition function for BO. A larger hypervolume corresponds to a better trade-off among objectives, with the true Pareto front achieving the maximum possible hypervolume. To efficiently maximize the HVI, a batch of candidate solutions $\boldsymbol{X}_{B}$ is selected in a sequential greedy fashion from the set $\boldsymbol{X}$, where $\left| X \right|=P=1,000$ across all tasks. This strategy ensures both computational efficiency and the progressive enhancement of solution quality.

The four-round AL process effectively expands the solution front toward high-performance regions across all objectives (**Figure S8** and **Figure 3a**). The initial 30 experimental formulations (**Figure S8a**) show a sparse distribution in the three-objective space, with several candidates achieving either high strength or toughness but few exhibiting satisfactory levitation height (>0.13 mm). After the first AL round (**Figure S8b**), the model identifies candidates enriched in toughness (≥6 MJ/m^3^) and moderate strength (55–60 MPa), with modest levitation improvements, reflecting early-stage surrogate effectiveness. In Round 2 (**Figure S8c**), the Pareto front shifts toward the levitation axis, with multiple candidates surpassing 0.18 mm while maintaining robust mechanical properties. Round 3 (**Figure S8d**) explores boundary regions, introducing additional non-dominated points that indicate improved balance among objectives and enhanced model generalization. By Round 4 (**Figure 3a**), the selected samples (triangles, squares, stars, and diamonds) converge toward the Pareto-optimal region, simultaneously achieving strength >55 MPa, toughness >6 MJ/m^3^, and levitation height >0.14 mm.

We assess convergence using the hypervolume indicator (HVI) with normalized objectives (**Figure S9**). From the baseline formed by the initial dataset, the first AL round yields an absolute hypervolume gain of 39.14, corresponding to a 22.09% relative increase, indicating that the acquisition policy quickly identifies higher-value regions not sampled initially. Subsequent rounds produce smaller relative gains of 3.16% and 3.54%, consistent with densification of an already improved Pareto front. In the fourth round the incremental gain is approximately 0.00% within numerical tolerance, which we interpret as a practical plateau under the current objective bounds and acquisition settings. This behaviour suggests that four AL iterations are sufficient to exhaust the accessible improvements for the present design space and experimental budget, and that further rounds would provide diminishing returns.


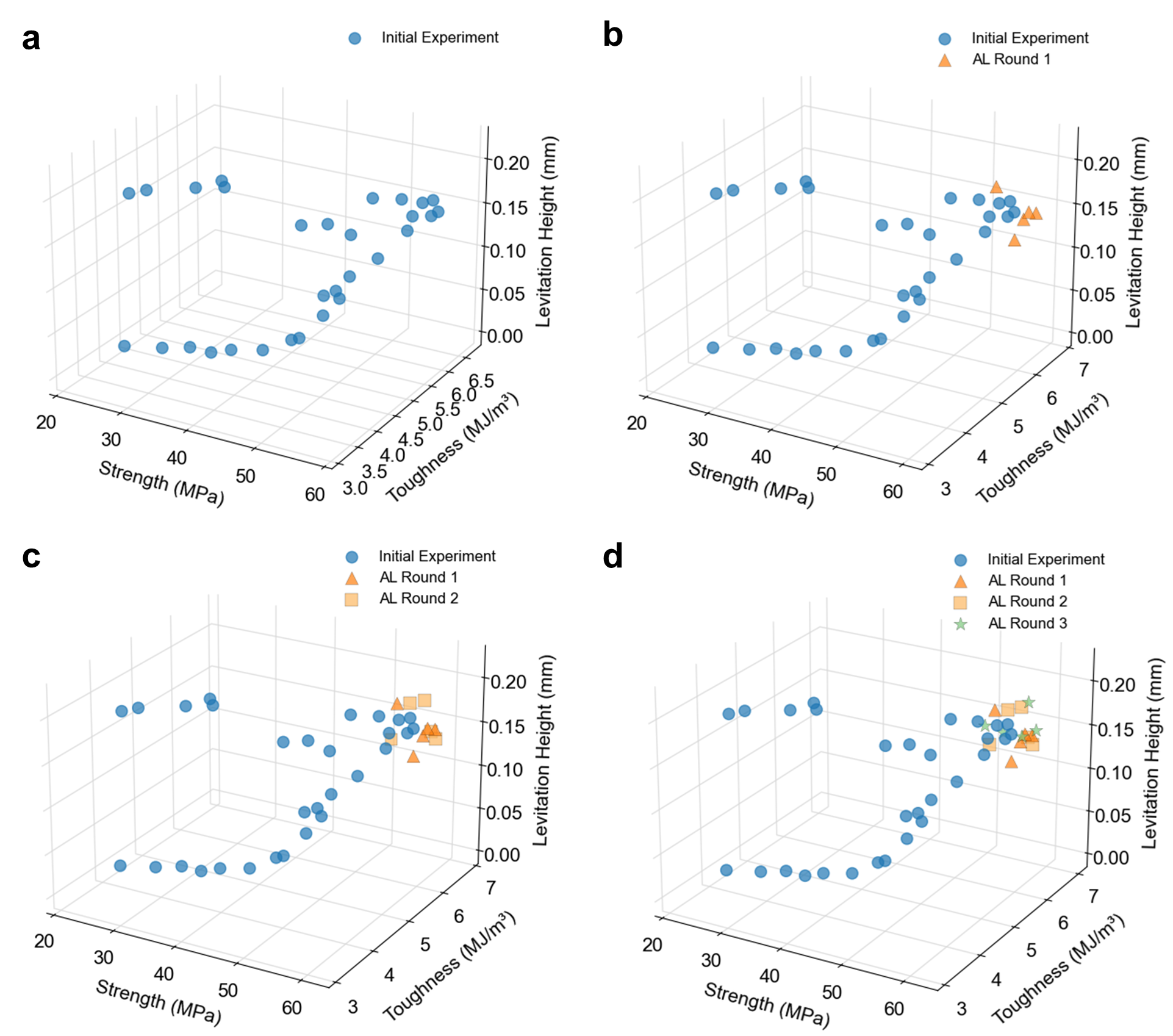


**Figure S8. Active learning (AL) evolution in the three-objective performance space. a**. Initial experimental data (blue circles). **b**. Round 1 (orange triangles). **c**. Round 2 (yellow squares). **d**. Round 3 (green stars). The plot shows 3D performance space: Strength (MPa), Toughness (MJ/m³), and Levitation Height (mm).


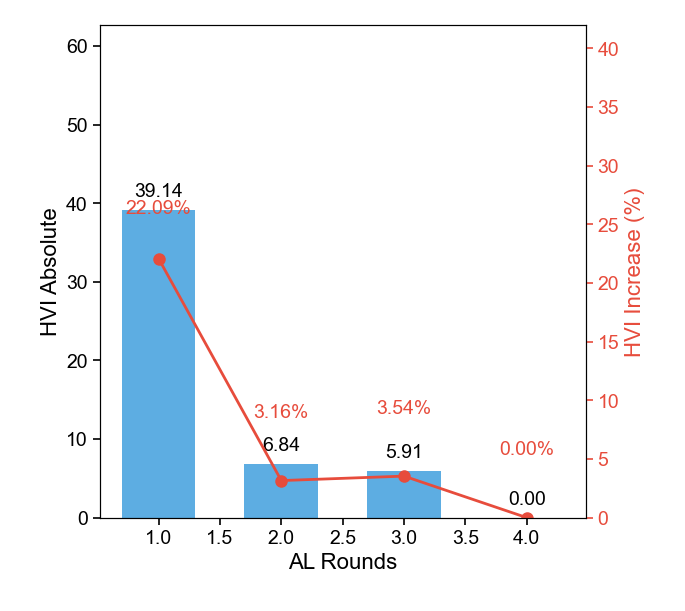


**Figure S9 Convergence of the Active Learning (AL) process evaluated by hypervolume improvement (HVI).** The first AL round yields a substantial hypervolume gain of 39.14, corresponding to a 22.09% expansion of the Pareto front. Subsequent rounds deliver progressively smaller improvements (3.16%, 3.54%), reflecting densification within already explored regions. By Round 4, the HVI increment approaches 0.00%, indicating convergence toward the Pareto frontier and confirming both the efficiency of the acquisition strategy and the sufficiency of the experimental budget.

**Note 3.4 Predictive uncertainty analysis**
To assess the impact of AL on surrogate model confidence, we quantitatively analyze the Gaussian Process (GP) predictive uncertainty (standard deviation, σ) before and after the four AL rounds (**Figures S10** and **S11**). In the initial PSL model (**Figure S10**), the median predictive uncertainty for strength is 4.38 MPa, with local values exceeding 6 MPa in sparsely sampled regions of the composition space. Toughness predictions exhibit a median σ of 0.50 MJ/m^3^, while levitation predictions remain comparatively stable with a median σ about 0.017 mm, reflecting simpler structure–function correlations for that property.

After four AL rounds (**Figure S11**), the median uncertainty in strength decreases to 2.54 MPa (−42.0%), and toughness uncertainty is reduced to 0.29 MJ/m^3^ (−39.6%). Levitation height uncertainty remains low and tightly distributed throughout. These reductions are clearly reflected in the violin plots (**Figures S10d** and **S11d**), which show substantial narrowing in σ distributions for both strength and toughness. Furthermore, the 3D composition-resolved σ maps (**Figures S11a–c**) reveal a more uniform and homogeneous uncertainty landscape, indicating that the model has achieved consistent confidence across the full design domain.

This comparative analysis demonstrates that AL not only enhances predictive performance but also systematically reduces surrogate uncertainty. By prioritizing samples with high expected information gain, AL efficiently drives the PSL model toward greater reliability and generalization, particularly in high-performance regions. The concurrent reduction in predictive uncertainty and the plateauing of hypervolume improvement (**Figure S9**) concomitantly mark the convergence of the optimization process, validating the effectiveness of the closed-loop PSL–AL framework.


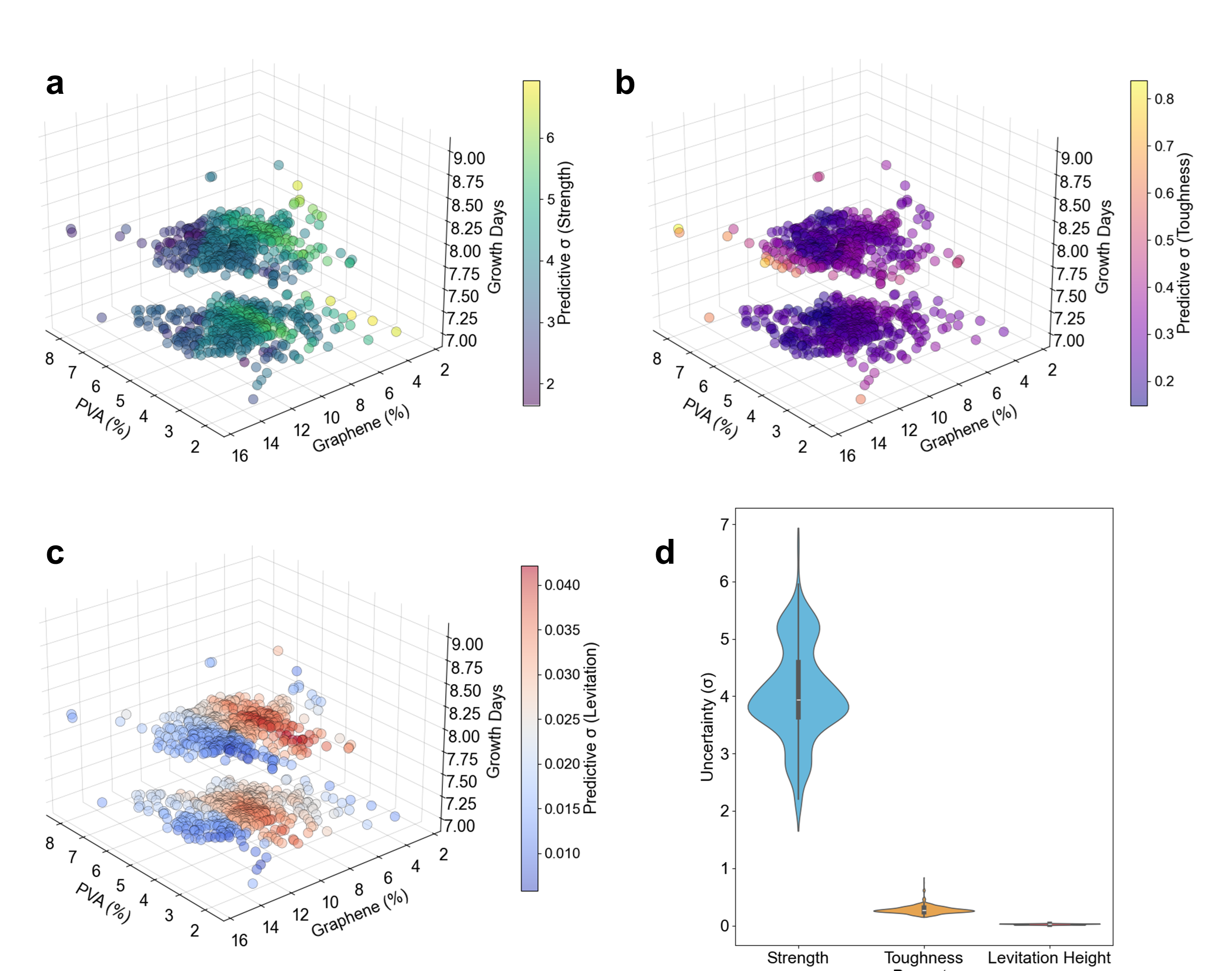


**Figure S10 Predictive uncertainty distributions of PSL models prior to AL. a–c**. Three-dimensional composition–space maps of the GP predictive standard deviation (σ) for strength (**a**), toughness (**b**), and levitation height (**c**). Color scale indicates the magnitude of σ at each design point. **d**. Violin plots showing the marginal distributions of σ for the three objectives, summarizing overall model uncertainty before AL.


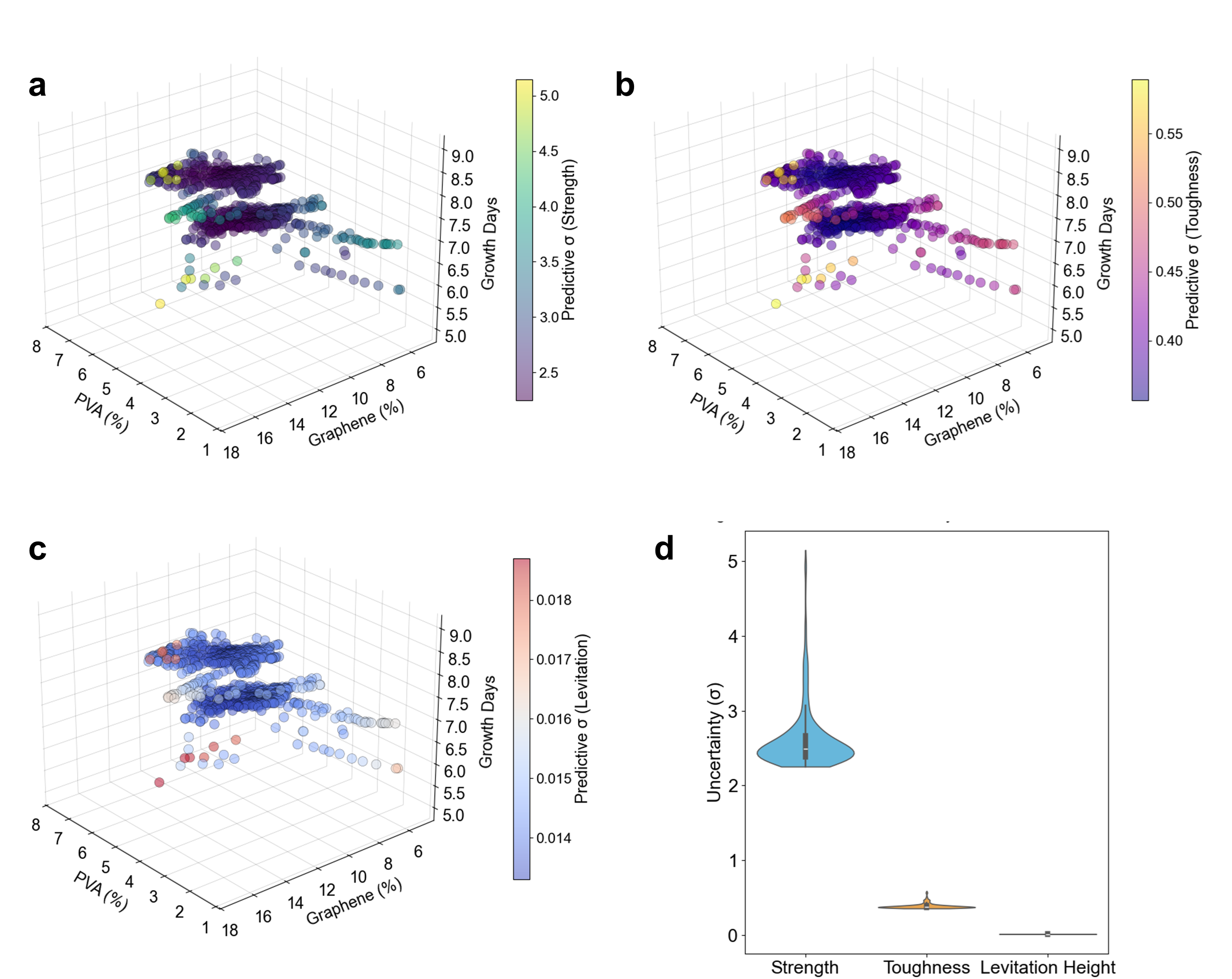


**Figure S11 Predictive uncertainty of the optimized PSL model after four rounds of AL.** **a–c**. Three-dimensional composition–space maps of the GP predictive standard deviation (σ) for strength (**a**), toughness (**b**), and levitation height (**c**). Color scale indicates the magnitude of σ at each design point. **d**. Violin plots of the marginal σ distributions for the three objectives, showing reduced uncertainty and more homogeneous distributions after AL.

**Note 3.5 SHAP value computation**

To interpret the influence of compositional and processing variables on predicted properties, we apply SHAP (SHapley Additive exPlanations) analysis to the trained surrogate model. SHAP is a model-agnostic interpretability framework grounded in cooperative game theory that attributes the marginal contribution of each input feature to individual predictions.

Here, SHAP is used to explain the outputs of a multi-output regression model predicting tensile strength (MPa), toughness (MJ/m^3^), and levitation height (mm) from three input features: PVA content (%), graphene content (%), and mycelium growth days. The input set for analysis comprises 1000 formulations generated by the PSL model, which maps preference vectors (λ ∈ Δ^3^) to design variables (x ∈ ℝ^3^). These PSL-generated candidates span a wide range of trade-offs and represent a diverse set of high-performance formulations.

SHAP values are computed using the *shap* Python package (v0.42.1) with the Kernel SHAP algorithm applied independently to each output dimension. For each formulation, SHAP assigns a quantitative contribution to each input variable relative to a reference background distribution. The resulting SHAP distributions are visualized in summary plots **(Figure 3e**), which highlight the relative importance and directional impact of the three features across all objectives. This analysis provides transparent interpretation of the surrogate model and clarifies which compositional or temporal factors most strongly shape the multi-objective performance landscape.

**Note 3.6 Preference-guided selection of candidate solutions from the high-performance region.**

To evaluate the multi-objective optimization capability of the PSL–AL framework, we construct a three-dimensional performance landscape of 1000 candidate solutions generated by the trained PSL model (**Figure S12**). Each point represents a predicted performance tuple f(λ), obtained by mapping a user-defined preference vector (λ ∈ Δ^3^) to an optimal formulation x(λ) and evaluating it with the surrogate model.

To illustrate the flexibility and interpretability of this approach in resolving trade-offs, three representative solutions on the Pareto frontier are highlighted (**Table S1**). A balanced design (λ = 0.35, 0.35, 0.30, red square) achieves 60.1 MPa strength, 6.20 MJ/m^3^ toughness, and 0.16 mm levitation. A mechanical-dominant design (λ = 0.45, 0.45, 0.10, green circle) prioritizes strength (60.0 MPa) and toughness (6.22 MJ/m^3^) with slightly reduced levitation (0.15 mm). A levitation-dominant design (λ = 0.25, 0.25, 0.50, blue triangle) maximizes levitation (0.17 mm) while maintaining robust mechanical performance (59.9 MPa, 6.19 MJ/m^3^). In all three cases, the PSL model identifies an optimal growth duration of 8 days, highlighting its ability to jointly optimize compositional and processing parameters.

Despite subtle compositional differences, these solutions occupy distinct regions of the performance space, reflecting different application priorities—mechanical robustness versus levitation responsiveness. The broader PSL solution set is shown as a gray point cloud, while high-performance regions satisfying practical thresholds (strength > 58 MPa, toughness > 6 MJ/m^3^, levitation > 0.14 mm) are enclosed by a pink envelope.

Beyond visualization, this λ → x → f(λ) mapping provides a programmable interface for rational formulation design. By querying the PSL model with custom preference vectors, researchers can retrieve candidate formulations tailored to specific objectives, thereby avoiding the inefficiencies of traditional trial-and-error strategies and accelerating the development of multifunctional composites.


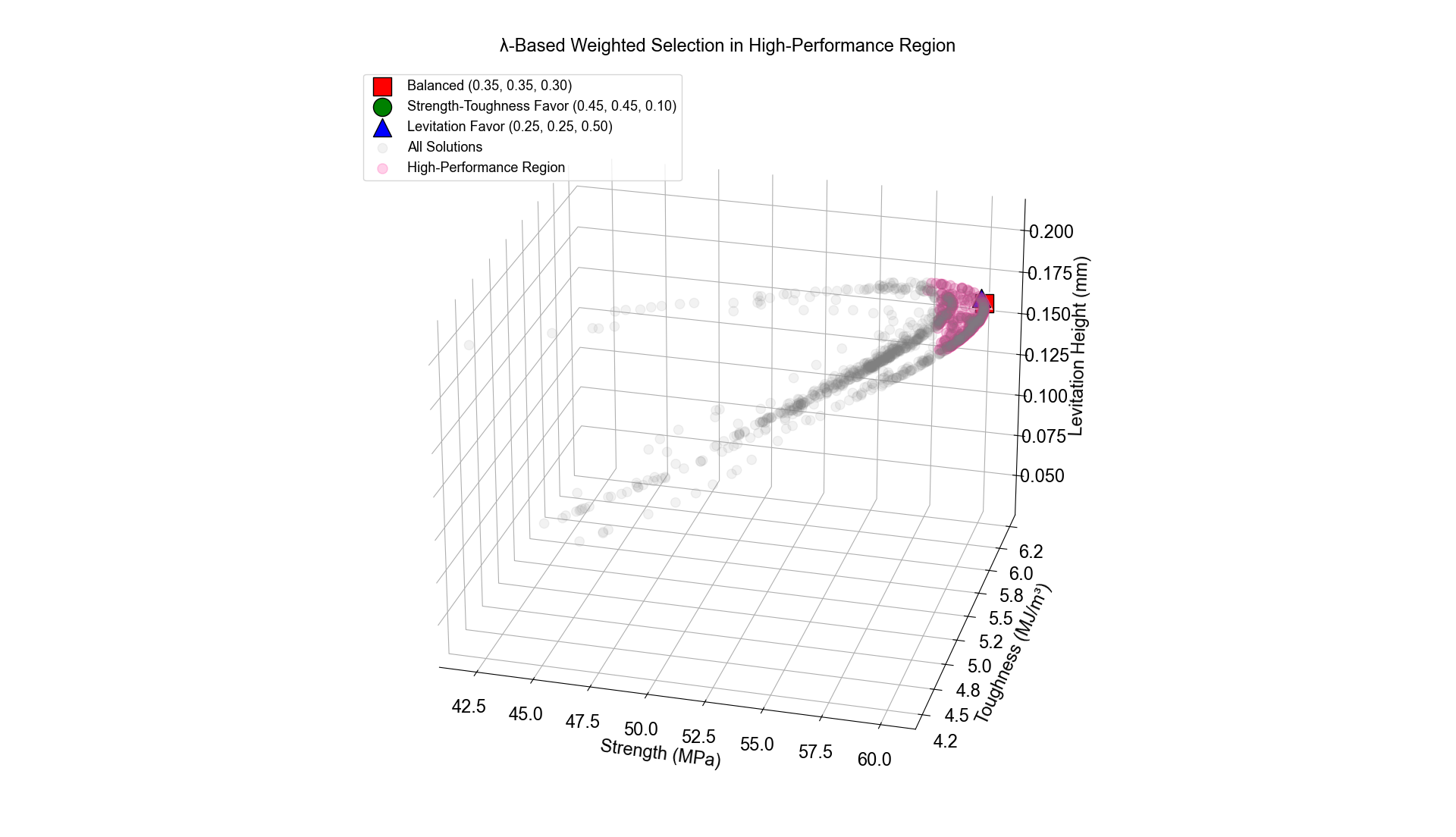


**Figure S12 Preference-guided selection of candidate solutions from the high-performance region.** Three-dimensional scatter plot of 1000 candidate solutions generated by the PSL model in the performance space defined by strength, toughness, and levitation height. Gray points represent all predicted solutions, while the pink region highlights the subset that simultaneously satisfies high-performance thresholds for all three objectives. Three annotated points correspond to representative preference vectors: balanced (red square), strength–toughness favoring (green circle), and levitation favoring (blue triangle), exemplifying the PSL model’s capability to generate functionally tailored material designs across the Pareto front.

**Table S1**. **Representative formulations and processing data selected from the PSL-predicted Pareto set based on different preference vectors (λ).**

| Preference Type | λ (Strength, Toughness, Levitation) | PVA (wt%) | Graphene (wt%) | Days | Strength (MPa) | Toughness (MJ/m^3^) | Levitation Height (mm) |
| --- | --- | --- | --- | --- | --- | --- | --- |
| Balanced | (0.35, 0.35, 0.30) | 5.234 | 11.602 | 8 | 60.11 | 6.20 | 0.16 |
| Strength–Toughness Favor | (0.45, 0.45, 0.10) | 5.397 | 11.554 | 8 | 60.02 | 6.22 | 0.15 |
| Levitation Favor | (0.25, 0.25, 0.50) | 5.196 | 11.884 | 8 | 59.98 | 6.19 | 0.17 |

**Note 4. Experiment validation and mechanism analysis**

To isolate the contributions of fillers and bio-assisted interfaces, we benchmark two orthogonal controls (**Figure S13**): a solution-blended PVA–PEG–graphene composite without mycelium and a mycelium-assembled PVA composite without PEG–graphene. The blended sample attains higher strength (approximately 41 MPa) but fractures abruptly at low strain, resulting in a toughness of 2.1 MJ/m^3^. In contrast, the mycelium-assembled PVA undergoes large deformation with lower strength (about 33 MPa) yet nearly doubles the toughness 4.6–4.8 MJ/m^3^). This comparison indicates that nanosheet fillers chiefly increase strength while embrittling the matrix, whereas hyphal assembly dissipates energy and improves crack resistance. When both mechanisms are integrated in MGCs (strength > 58 MPa, toughness > 6.0 MJ/m^3^, **Figure 4**), the resulting bio-assisted, nanosheet-pinned interfaces deliver synergistic reinforcement that overcomes the classical strength–toughness trade-off not achievable by blending or bio-assembly alone.


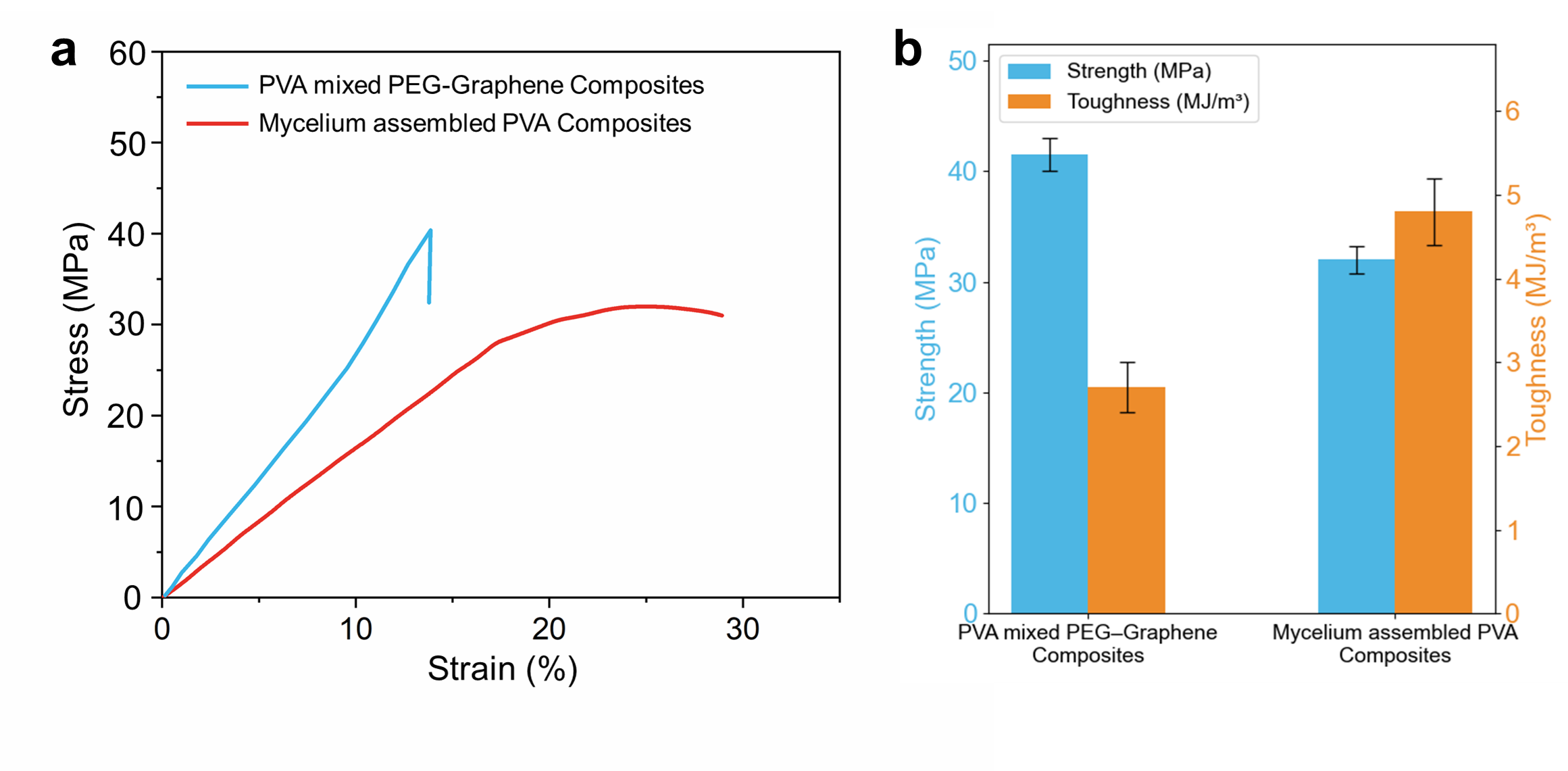


**Figure S13 Comparison of mechanical performance between PEG–graphene blended PVA composites and mycelium-assembled PVA composites.** **a**. Stress–strain curves of PVA composites prepared by direct mixing of PEG-intercalated graphene (blue) and by mycelium-guided assembly (red). **b**. Quantitative comparison of strength and toughness.

**Note 5. Mechanical benchmarking of MGCs**

To contextualize the mechanical performance of the MGCs, we benchmark the material against both graphene reinforced polymer composites and broader classes of structural materials. **Figure S14a** presents an Ashby-style plot of specific strength versus specific toughness constructed from representative high-quality reports in the literature. The reported graphene reinforced polymer composites [14-19], including cellulose/rGO/PEG films, C-GOF thick films, rGO–NFC films, GO–WS/PLA bio-composites, and CNF–Fe₃O₄/LM/graphene films, cluster in the low-to-mid range of the Ashby space, with specific strengths typically below 5×10^-2^ MPa·m^3^/kg and specific toughness values between 10^-3^ and 10^-2^ MJ·m^3^/kg. In contrast, the MGCs developed in this work occupy a clearly separated region with substantially higher performance, achieving specific strength values near 1×10^-1^ MPa·m^3^/kg and specific toughness values approaching the upper bound of the data range. **Figure S14b** places the MGCs within the global material landscape. When plotted against density and strength, the MGCs align at the transition between natural cellular solids and lightweight composites, combining low density (~0.8–0.9 g/cm^3^) with strengths comparable to advanced polymeric materials. These benchmarking analyses illustrate that the MGCs exceed the mechanical performance envelope of existing graphene-reinforced bio-composites while simultaneously approaching the domain of engineered lightweight composites, thereby confirming their relevance as a high-performance and sustainable structural material.

**Table S2** **Benchmarking the specific strength and toughness of MGCs against representative bio-based composites**

| Material Type | Toughness (MJ/m^3^) |  | Tensile Strength (MPa) | Fracture Strain (%) | Density (kg/m^3^) | Reference |
| --- | --- | --- | --- | --- | --- | --- |
| Mycelium based composites | 0.25–2.25 |  | 0.5–3 | 0.5–2 | 150–340 | [5-9] |
| Plant cell based composites | 0.5–1.2 |  | 18.2–24.2 | 0.7-1.3 | 500–1000 | [10] |
| Biomatter hybrid bioplastic (waste cotton fiber and pollen particles) | 1.5–1.6 |  | 48–52 | 5–7 | 570–600 | [11] |
| Protein based bioplastic | 3–3.6 |  | 55–60 | 7–12 | 800–1200 | [12] |
| Spirulina cell based bioplastic | 1.3–4.3 |  | 25.5–57 | 0.5–4.5 | 900–1000 | [13] |
| MGCs (this work) | 6.05–6.2 |  | 58–63 | 18–20 | 800–890 | This study |

**`**

**Table S3 Benchmarking the specific strength and toughness of MGCs against representative graphene reinforced polymer composites**

| Material Type | Toughness  (MJ/m^3^) | Tensile Strength  (MPa) | Density  (g/cm^3^) | Reference |
| --- | --- | --- | --- | --- |
| Cellulose/Reduced Graphene Oxide/Polyethylene Glycol composite film | 0.73 | 30.6 | 1300-1500 | [14] |
| PLA (Semi-crystalline grade) | <1 | 50–70 | 1200–300 | [15] |
| C-GOF thick graphene oxide film | 3.4-5.4 | 75-95 | 1800 | [16] |
| GO-functionalized wheat-straw / PLA bio-composite | 3.8 | 30-40 | 1200–1250 | [17] |
| CNF–Fe_3_O_4_ / liquid-metal / graphene film | 2.72 | 55.3 | 1200-1300 | [18] |
| rGO–NFC film | 4-5 | 80-90 | 1400-1500 | [19] |
| MGCs (this work) | 6.05–6.2 | 58–63 | 800-890 | This study |

GO: Graphene Oxide


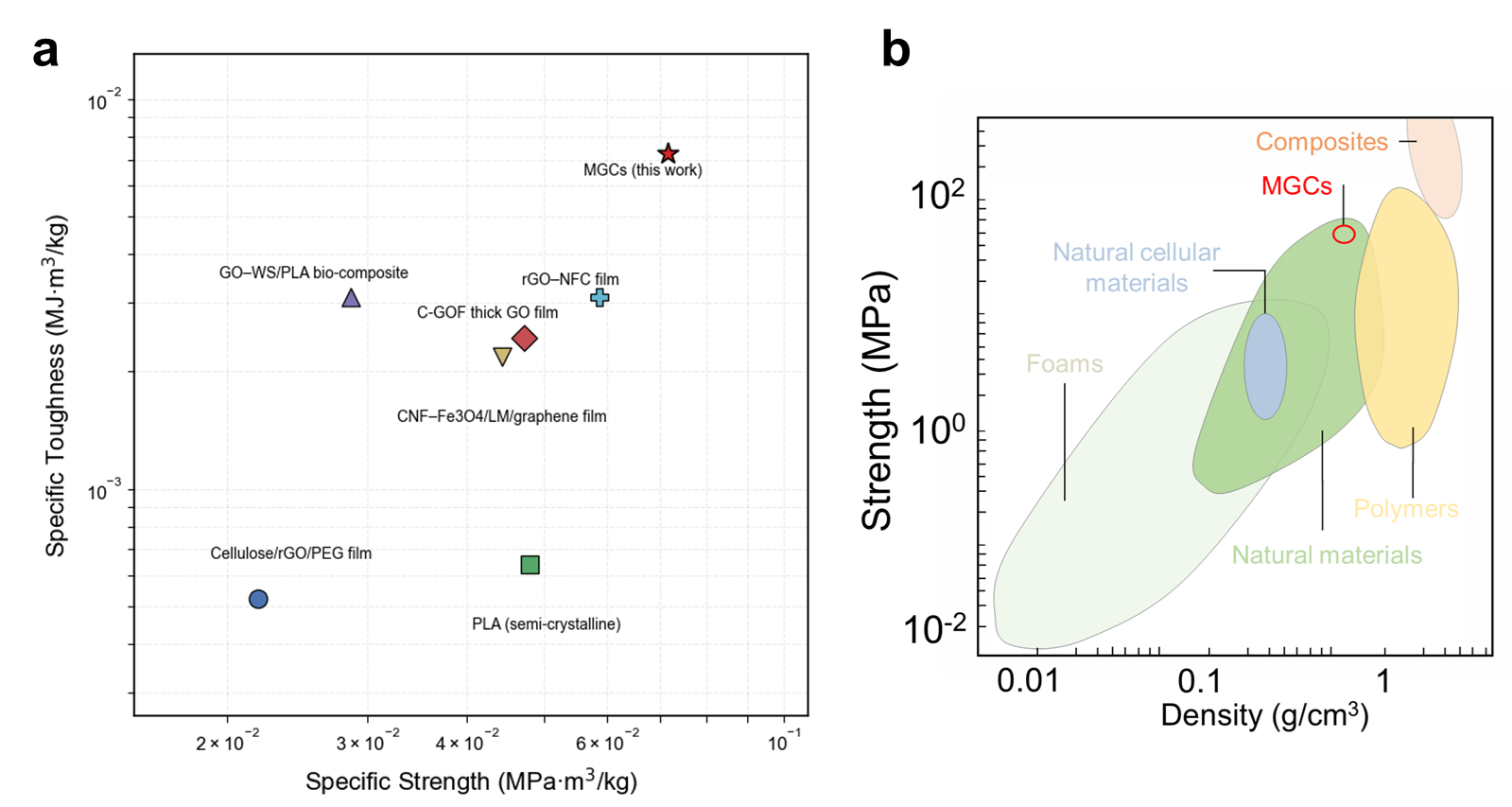


**Figure S14 Performance benchmarking of MGCs against state-of-the-art graphene-reinforced composites and structural material classes. a**. Ashby plot of specific strength versus specific toughness comparing the MGCs developed in this work (red star) with representative graphene-reinforced polymer composites reported in recent literature, including GO–WS/PLA bio-composites, cellulose/rGO/PEG films, C-GOF thick films, rGO–NFC films, and CNF–Fe_3_O_4_/LM/graphene films. The MGCs occupy a higher-performance region of the Ashby space, positioned above all reference graphene composites in both specific strength and specific toughness. **b**. Positioning of MGCs within the broader landscape of structural materials. The MGCs fall at the boundary between natural cellular materials and engineered composites, combining a low density of ~0.80–0.89 g/cm^3^ with strength approaching those of lightweight polymers and fiber-reinforced composites. This positioning highlights the advantage of bio-assisted nanosheet-pinned interfacial strategy in delivering high mechanical performance at low mass density.


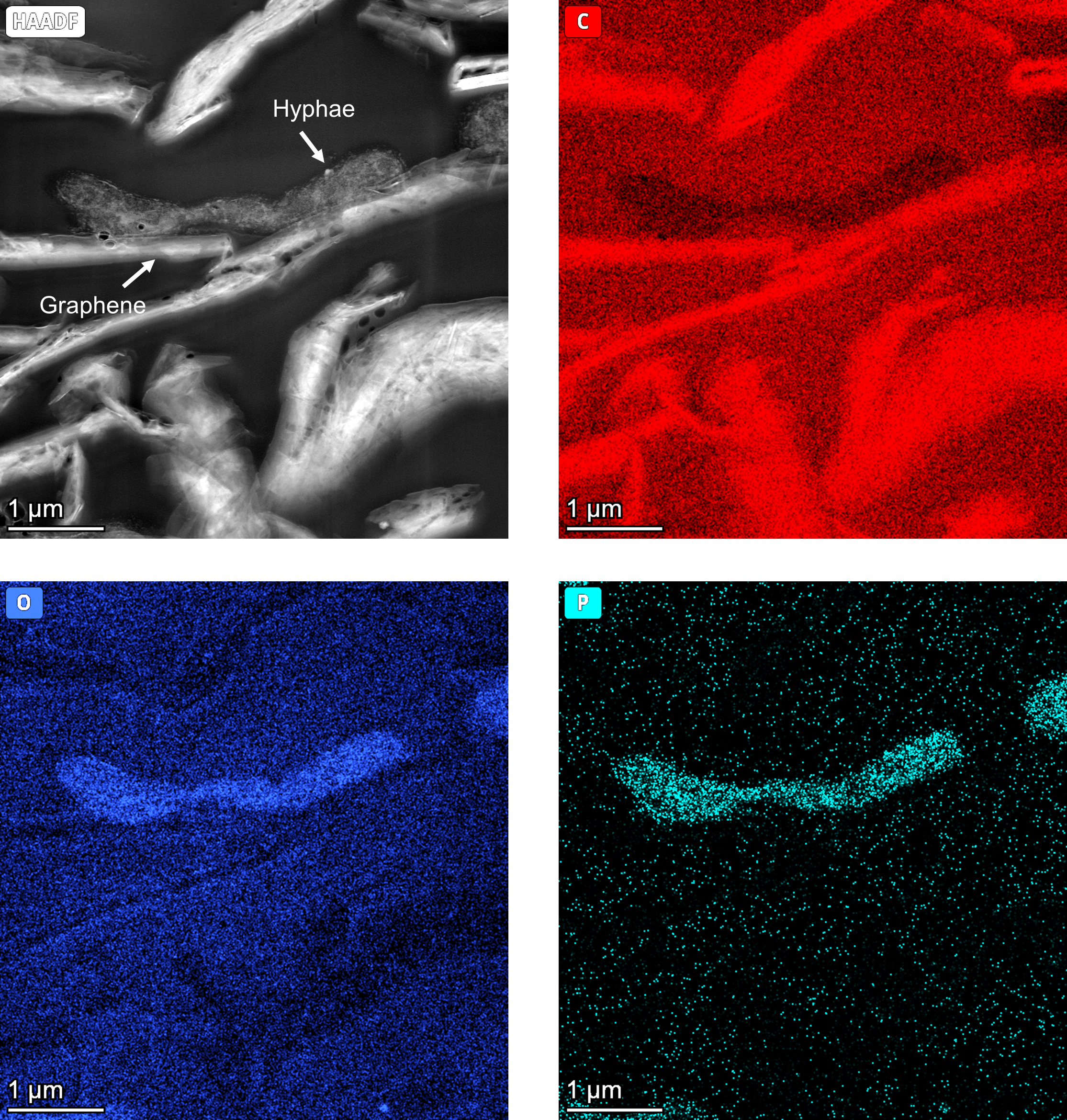


**Figure S15 Interfacial integration of the balanced design MGCs revealed by HAADF–EDS mapping.** High-angle annular dark-field (HAADF) imaging (top left) and corresponding energy-dispersive X-ray spectroscopy (EDS) elemental maps of C (red), O (blue), and P (cyan) demonstrate intimate contact and interpenetration between fungal hyphae and graphene sheets. The presence of phosphorus along the hyphal contour indicates PEG-mediated interfacial binding. The spatial correlation among graphene (C-rich), hyphae (O- and P-rich), and their physical overlap provides direct evidence of hybrid interface formation at the nanoscale.


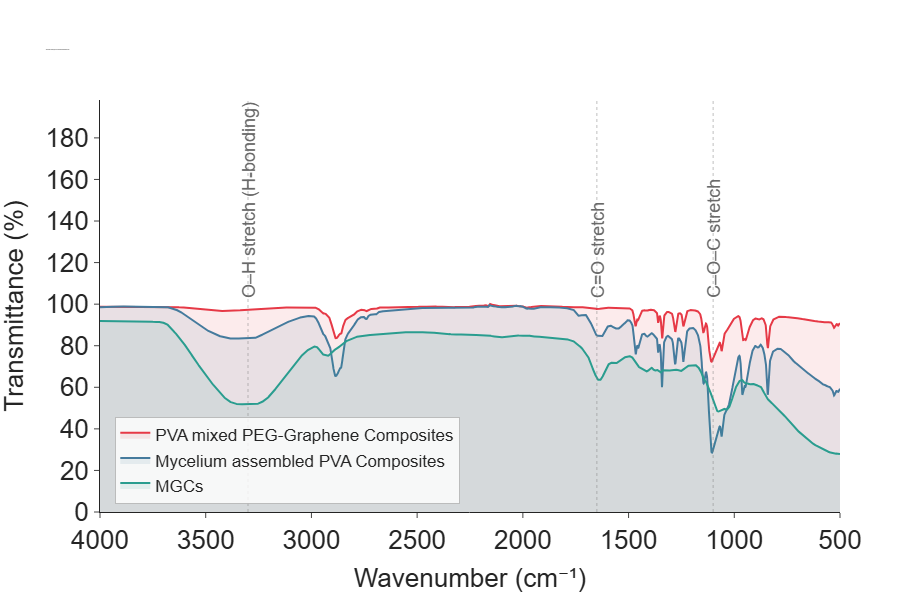


**Figure S16 FTIR spectra of composite samples with different interfacial structuring strategies.** Comparison of PVA mixed PEG–Graphene composites, mycelium-assembled PVA composites, and the balanced design MGCs. Enhanced hydrogen bonding (O–H stretching) and shifts in C=O and C–O–C bands are observed in the mycelium-based samples, indicating stronger and more complex interfacial interactions.

**Note 6. Molecular dynamics (MD) modeling and simulation**

To investigate the interfacial mechanisms underlying the enhanced mechanical properties of the balanced-design MGCs, we perform all-atom molecular dynamics (MD) simulations using the LAMMPS package.

To preserve essential interfacial features while maintaining computational tractability, we adopt a simplified yet representative modeling approach. We model the fungal component with two wall polysaccharides: chitin and β-1,3-glucan. Chitin is represented as linear N-acetyl-D-glucosamine oligomers (10–30 units per chain) that preserve the acetamide and hydroxyl functionalities responsible for hydrogen bonding and chain rigidity. β-1,3-glucan oligomers (10–30 units) provide additional hydrogen-bond donors/acceptors and entanglement. The PVA matrix is represented by five chains of 30–40 repeat units each, sufficient to mimic the flexible polar backbone. PEG intercalants are modeled as chains of 10–20 ethylene glycol units inserted between graphene sheets to reproduce interfacial sliding and stress dissipation. Graphene is represented as single layers of 5.0 × 5.0 nm^2^ with hydrogen-passivated edges. All systems are constructed with periodic boundary conditions in the x–y plane and semi-open boundaries along the z-axis. The resulting simulation boxes contain approximately 10000–15000 atoms, enabling nanosecond-scale simulations of interfacial dynamics at reasonable computational cost. The interface modeling as showing in **Figure S17**.

Energy minimization is first conducted using the conjugate gradient method. The systems are then equilibrated under the NVT ensemble at 300 K for 500 ps, followed by an NPT ensemble at 1 atm for 1 ns to relax density and internal stresses. All interactions are parameterized using the COMPASS force field, which accounts for bonded terms, van der Waals interactions, electrostatics, and explicit hydrogen bonding.

To probe local interfacial deformation and stress-transfer mechanisms, the equilibrated composite segments are subjected to uniaxial tensile deformation along the z-axis using the *fix deform* command. A constant engineering strain rate of 1 × 10^-4 ps^-1 is applied while constraining a 2 Å region at the bottom of the box. This loading protocol is used to examine interfacial sliding, polymer-chain rearrangement/disentanglement, hydrogen-bond evolution, and local stress-transfer pathways at the molecular scale. Owing to the timescale limitation inherent to atomistic MD, this loading rate is substantially higher than experimental rates and is therefore intended to probe molecular-scale deformation mechanisms rather than quantitatively reproduce the macroscopic mechanical response.


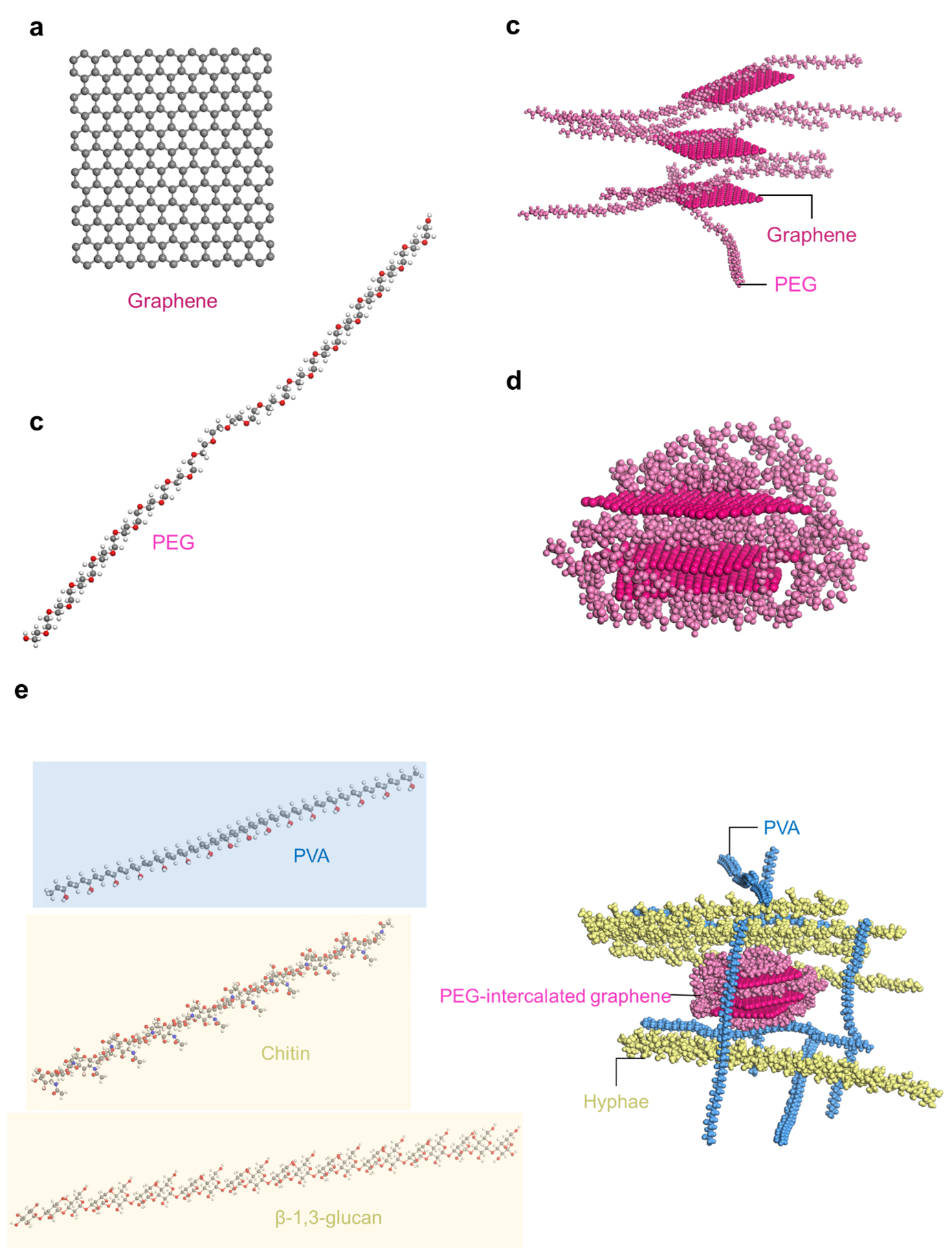


**Figure S17 Molecular dynamics (MD) simulation model of the balanced design MGCs.** **a**. Graphene sheet. **b**. PEG. **c**. PEG intercalation graphene initial model. **d**. Compact aggregation of PEG-intercalated graphene with enhanced interface area and conformal alignment. **e**. Molecular components of the biohybrid matrix including PVA, hyphae (chitin and β-1,3-glucan). The right panel illustrates the integrated interface where PEG-intercalated graphene is embedded within a 3D network of mycelial hyphae and PVA chains.

**Note 7. Finite-Element (FE) simulation**

Finite-element simulations are conducted to evaluate how nanoscale interfacial sliding influences the microscale stress distribution within the MGCs. A three-dimensional representative microstructural model with dimensions of 2.4 mm × 0.1 mm × 0.02 mm is constructed based on the nanosheet distribution observed in the CT scan results (**Figure S18**). Polygonal graphene nanosheets are embedded within a continuous polymer matrix, and all boundaries are smoothed to avoid mesh-induced artefacts. The material constants used in the simulation are listed in **Tables S4** and **S5**.

The model is discretized using a refined triangular mesh. Incremental tensile displacement is applied along the horizontal axis, while the opposite edge is fixed to emulate uniaxial stretching. Plane-strain conditions are applied to approximate the finite thickness of the composite film.


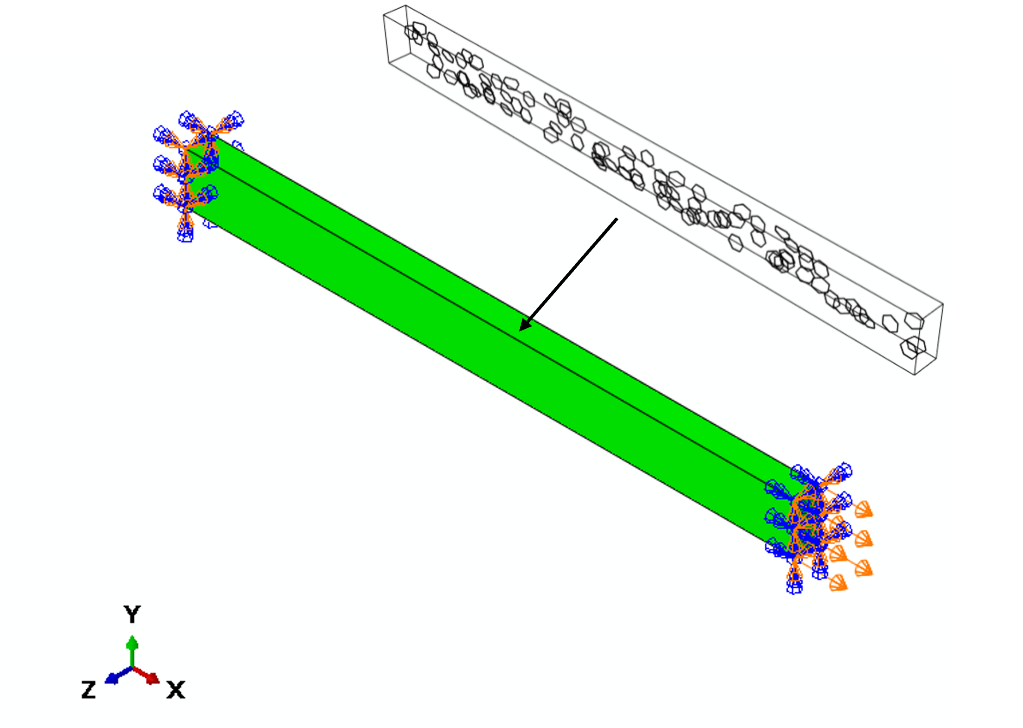


**Figure S18** **FE model of nanosheet–matrix interfacial deformation under uniaxial tension.**

**Table S4 The material properties of the nanosheet.**

| Properties | Value | Units |
| --- | --- | --- |
| Density | 2.267 | g/cm^3^ |
| Young’s modulus | 1.02 | TPa |
| Position’s ratio | 0.16 |  |
| Strength | 120 | GPa |

**Table S5 The material properties of the matrix.**

| Properties | Value | Units |
| --- | --- | --- |
| Density | 0.85 | g/cm^3^ |
| Young’s modulus | 3510 | MPa |
| Position’s ratio | 0.36 |  |
| Strength | 48 | MPa |

The von Mises stress distribution is evaluated at each strain step (**Figure S19**), revealing a clear progression of deformation modes. At low strain, stress localizes around nanosheet edges where mechanical constraints are highest. As strain increases, interfacial shear bands emerge along the nanosheet–matrix boundaries, marking the onset of controlled nanosheet sliding. At larger deformation, these shear zones expand and merge into continuous load-transfer pathways aligned with the tensile direction, enabling efficient redistribution of stress across the microstructure. This mesoscale evolution provides a bridge between the atomistic sliding and disentanglement processes identified by MD simulations and the macroscopic toughening behavior observed in tensile experiments.


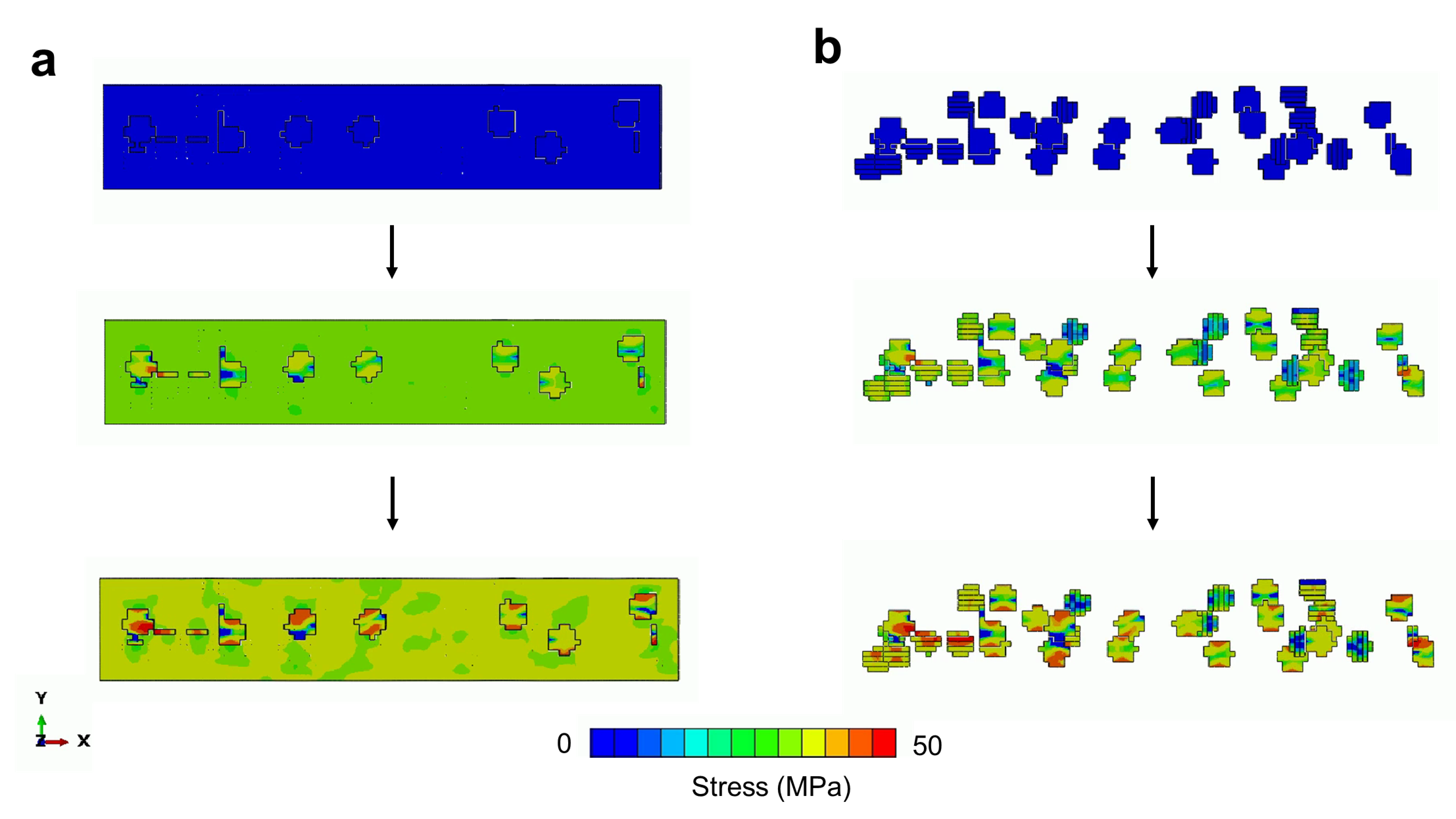


**Figure S19 Multiscale FE analysis of MGCs deformation. a**. Von Mises stress maps under increasing tensile strain. **b**. Stress evolution within the graphene-sheet ensemble.

**Note 8. Diamagnetic levitation simulation**

To understand the stable diamagnetic levitation phenomenon and quantitively study the levitation height, we model the diamagnetic levitation numerically using Finite Element Method (FEM).

**Modelling**. To simulate diamagnetic levitation, we utilize COMSOL Multiphysics 6.0 to model the magnetic field and calculate the diamagnetic force. The 3D geometry of the plate and magnets is shown in **Figure S20a**. To account for the actual shapes of magnets, the magnets feature a round fillet of 0.1mm at their edges. In simulating the magnetic field in free space, we create a layer of air and another layer of zero magnetic potential (**Figure S20b**). The mesh, consisting of free tetrahedral elements, is presented in **Figure S20c** and **S20d**. Additionally, the geometry and mesh of the levitation with a circular plate and magnets are illustrated in **Figure S20e-h**.

**Calculating**. The total magnetic force $\boldsymbol{F}_{B}$ from the magnetic field $\boldsymbol{B}$ is expressed by:

$\boldsymbol{F}_{B}=\int_{V} \left( \boldsymbol{M}\cdot\nabla\right)\boldsymbol{B}dV=\frac{1}{2\mu_{0}}\int_{V} \nabla(\chi_{x}B_{x}^{2}+\chi_{y}B_{y}^{2}+\chi_{z}B_{z}^{2})dV$ (1)

where $\boldsymbol{M}$ is the magnetization, ***B* =** (***B*_x_, *B*_y_, *B*_z_**) is the magnetic flux density, $V$ is the volume of the plate, $\chi=(\chi_{x}, \chi_{y}, \chi_{z})$ is the magnetic susceptibility of the material.

The magnetic flux density $\boldsymbol{B}$ and magnetic field strength ***H*** of the permanent magnets can be obtained by solving the following equations:

$\nabla\cdot\boldsymbol{B}=0$ (2)

$\boldsymbol{B}=\mu_{0}\mu_{r}\boldsymbol{H}$ (3)

$H=-\nabla V_{m}$ (4)

where $\mu_{0}=4\pi\cdot{10}^{-7}(H/m)$ is the magnetic permeability of vacuum, and $\mu_{0}=1$ is the relative permeability of air, and $V_{m}$ is the magnetic scalar potential to be solved. The equations also have the boundary conditions with $B_{r}=1.4T$ which is the residual magnetic flux density of the permanent magnets and $V_{m}=0$ at the outermost layer of the model.

The *Magnetic Fields, No Currents* (*MFNC*) module is used to simulate the above equations numerically. By solving these equations, the magnetic scalar potential and magnetic field of the levitation are obtained. Once the magnetic field ($\boldsymbol{B}$) is determined, the magnetic force could be calculated using equation (1) in the main text through integration over the volume of the levitated plate.

The magnetic susceptibility of graphene is reported to be on the order of –10^-5^ to –10^-4^ [20, 21], with a representative value of approximately$\chi_{g}=-120\times{10}^{-6}$, consistent with its pronounced diamagnetic behavior observed in recent experimental and theoretical studies. The magnetic susceptibility of living microorganisms (mycelium) and polymers (PVA) in MGCs are negative but greater in magnitude compared to graphene. Therefore, the effective susceptibility of the MGCs can be calculated by the equation (5):

$\chi=v\chi_{g}$ (5)

where $v$ is the volume fraction of the graphene in the MGCs.

It is assumed that the distribution of graphene flakes is ordered in the magnetic field. By comparing the measured levitation height and the simulated height of a 3 mm plate (**Figure 5e**) and taking the volume fraction as fitting parameters, we could estimate $v=16\%$, consider that the distribution of graphene flake is ordered on the magnetic field. Finally, the effective susceptibility of the material is $\chi=19.2\times{10}^{-6}$.

**Simulation of light actuation.** To understand the light actuation phenomena of the levitating MGCs plate, time dependent simulations are conducted to simulate the dynamic response of a plate in a gravity and magnetic field due to laser heating. A circular MGCs plate with radius of 3 mm and thickness of 0.4 mm is simulated. The plate is levitating 0.2 mm above an array of 3 x 3 x 3 mm^3^ magnets with alternating magnetizations and remanent magnetic flux density of 1.4T (**Figure S21a)**. The model is surrounded by a spherical domain of air to represent the open air and another thin layer of infinite elements for computational efficiency purposes (**Figure S21b**). The meshes are demonstrated in **Figure S21c** and **S21d**.

To model the photothermal effect, the COMSOL’s *Heat Transfer in Solids* module is used. A laser with spot size of 2 mm in a Gaussian profile is model as sweeping from the centre of the plate to its edge at a speed of 60 mm/s. For demonstration purpose, the laser power is modelled as 5W, the thermal conductivity of the plate is 130 W/(m∙K), the heat capacity of the plate was 700 J/(kg∙K), the density of the plate is 890 kg/m^3^, the surface emissivity of the plate is 0.8.

The magnetic field and force are simulated using COMSOL’s *Magnetic Fields, No Currents* module with the same procedure as descripted in above. It has been known that the diamagnetism of graphene is weaker with increasing temperature. To account for the temperature dependent property, we model the susceptibility of MGCs as

$\chi_{T}=(-19.2+0.1\times\left( T-293.15 \right))\times{10}^{-6}$ (6)

where *T* is the temperature in Kelvin. Therefore, the magnetic forces applied to the plate in three axes can be written as

$F_{x}=\frac{1}{2\mu_{0}}\int_{V} \chi_{T}\frac{d}{dx}(B_{x}^{2}+B_{y}^{2}+B_{z}^{2})dV$ (7)

$F_{y}=\frac{1}{2\mu_{0}}\int_{V} \chi_{T}\frac{d}{dy}(B_{x}^{2}+B_{y}^{2}+B_{z}^{2})dV$ (8)

$F_{z}=\frac{1}{2\mu_{0}}\int_{V} \chi_{T}\frac{d}{dz}(B_{x}^{2}+B_{y}^{2}+B_{z}^{2})dV$ (9)

We then simulate the motion of the plate using COMSOL’s module *Solid Mechanics* by applying the calculated magnetic force and gravity force. When the total force applied to the plate is not zero, the plate will move. Since the plate has not physical constraints, its motion will be solely governed by the magnetic force and gravitation force.

We carry out simulations by simultaneously coupling the thermal effect of the laser, magnetic force from permanent magnets, and rigid body motion of the plate in the time domain. With time involving, the laser will increase the temperature of the MGCs plate. The rise in temperature leads to a decrease in the magnetic force, reducing it below the gravitational force. This imbalance in forces propels the plate into motion. As the laser is locally incident on the MGCs plate from the side, it imparts a lateral force, eventually initiating movement.

**Simulation of programming magnetic field distribution**. To regulate the distribution of the magnetic field, we introduce a 0.1mm thick gap between two adjacent magnets, as illustrated in the **Figure S21e-h**. The calculation method follows the diamagnetic levitation steps described earlier.


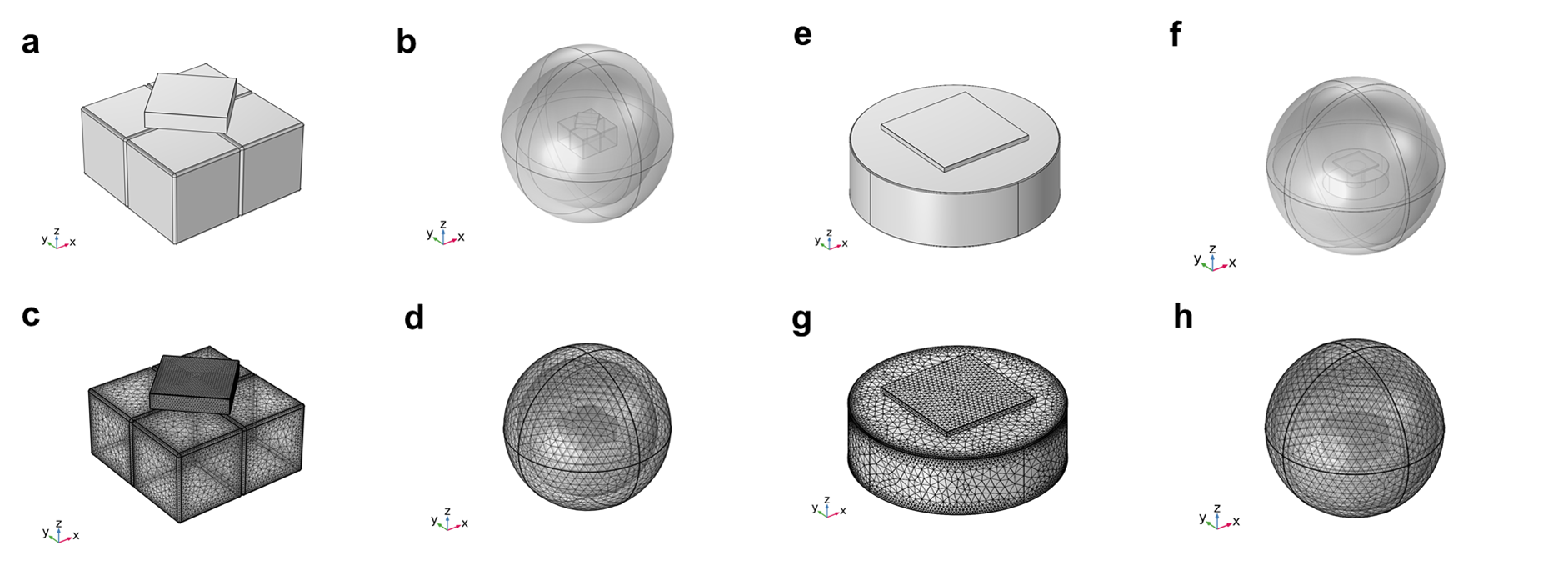


**Figure S20** **Diamagnetic levitation simulation modeling. a**. Geometry of a plate above four magnets. **b**. Geometry of the plate and magnets surrounded by a layer of air and a layer of zero magnetic potential. **c**. Mesh of the plate and magnets. **d**. Mesh of the whole models. **e**. Geometry of a circular plate above two circular magnets. **f**. Geometry of the plate and magnets surrounded by a layer of air and a layer of zero magnetic potential. **g**. Mesh of the plate and magnets. **h**. Mesh of the whole models.


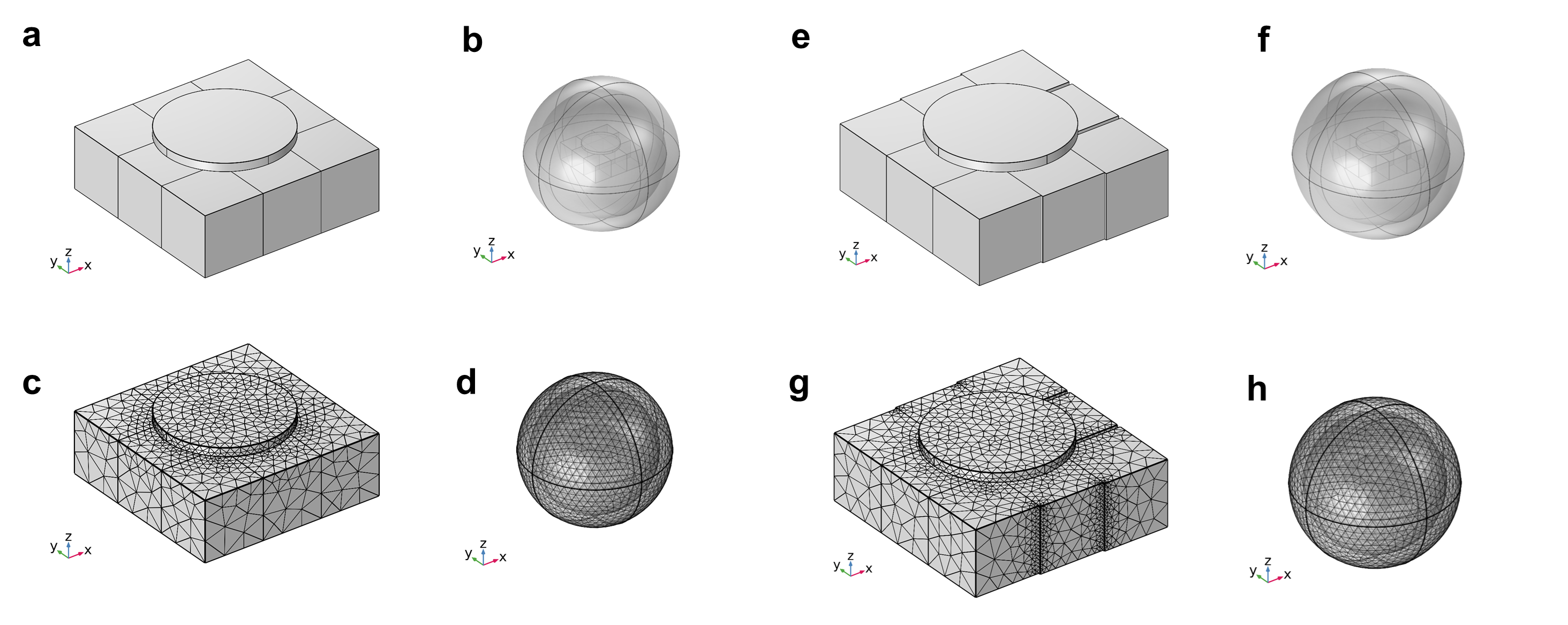


**Figure S21** **The simulation modeling of programing magnetic levitation micro robot.** Photo thermal actuation: **a**. Geometry of a circular plate above nine magnets. **b**. Geometry of the plate and magnets surrounded by a layer of air and a layer of zero magnetic potential. **c**. Mesh of the plate and magnets. **d**. Mesh of the whole models. Programming magnetic field distribution: **e**. Geometry of a circular plate above nine magnets with 0.1mm thick gap between two adjacent magnets. **f**. Geometry of the plate and magnets surrounded by a layer of air and a layer of zero magnetic potential. **g**. Mesh of the plate and magnets. **h**. Mesh of the whole models.

In our experiments, we observe that the plates cannot be levitated if the plate is too small or too big. To understand the stable diamagnetic levitation phenomenon and quantitively study the levitation height, we model the diamagnetic levitation numerically using FE. As shown in **Figure S22**, the equilibrium height depends strongly on plate dimensions. Increasing plate length from 1.8 to 3.3 mm progressively shifts the force–distance balance, with larger plates requiring stronger diamagnetic repulsion to counteract the higher gravitational load. The crossover points between diamagnetic and gravitational forces define stable levitation positions, demonstrating that both plate geometry and mass directly regulate levitation stability. This size-dependent scaling demonstrates the importance of dimensional control in designing levitated MGC systems.


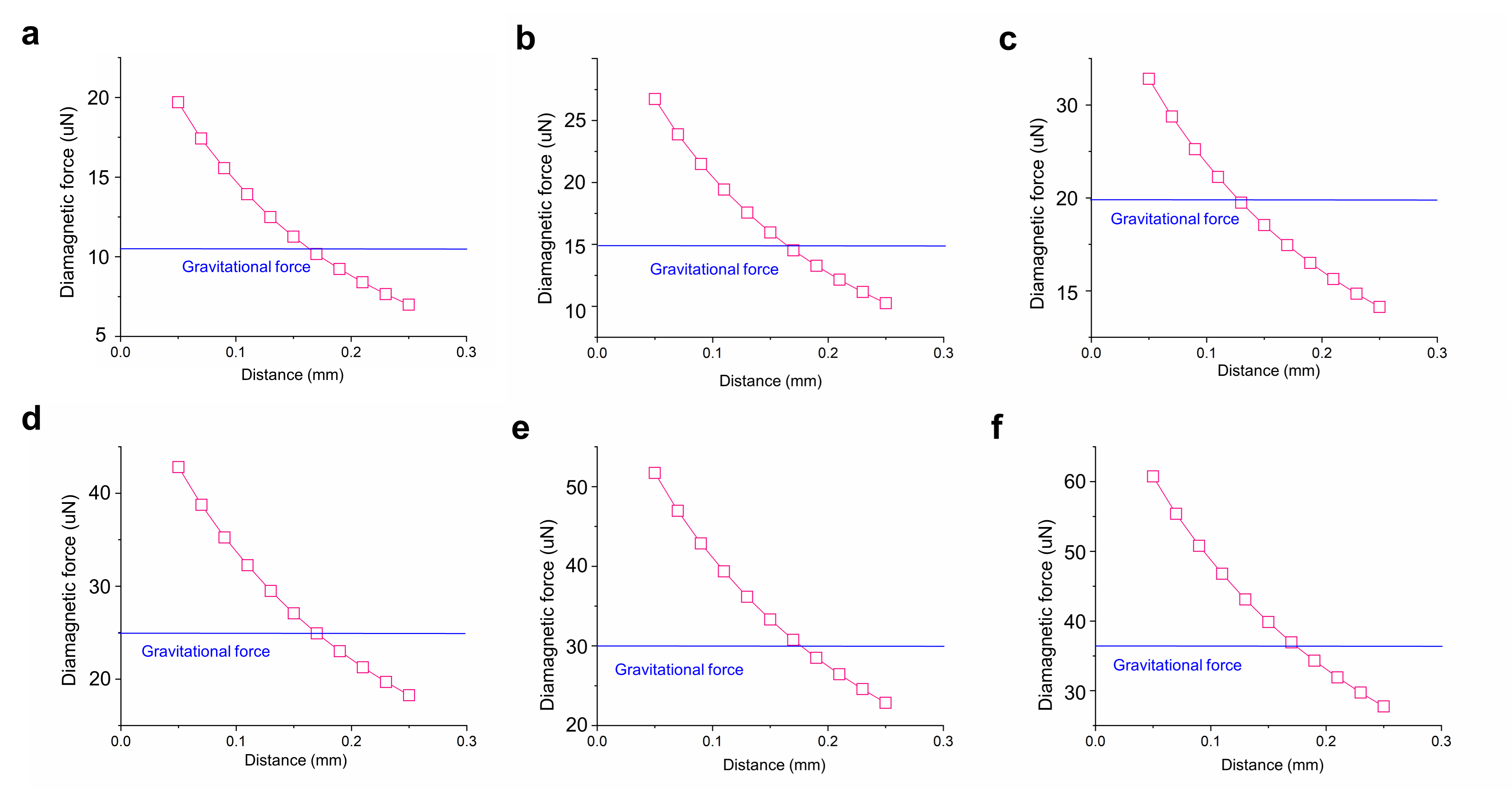


**Figure S22 Dependence of diamagnetic force on plate–magnet separation for MGC plates of varying sizes.** Diamagnetic forces are calculated as a function of the vertical distance between MGC plates and the underlying magnet array. Plate lengths are varied as follows: (**a**) 1.8 mm, (**b**) 2.1 mm, (**c**) 2.4 mm, (**d**) 2.7 mm, (**e**) 3.0 mm, and (**f**) 3.3 mm, while the thickness is kept constant at 0.64 mm. The pink curves represent the computed diamagnetic force as a function of separation, and the blue horizontal lines indicate the gravitational force of each plate. The crossover points between the two curves define the equilibrium levitation height.

Apart from rectangular magnets, we are also able to levitate circular plates with circular magnets (**Figure S23**). When placed above a circular permanent magnet, the composite stably levitates with an observable air gap (**Figure S23a**), demonstrating that the bio-assembled interfacial architecture can achieve sufficient magnetic flux exclusion while retaining mechanical integrity. FEM simulations of the magnetic field provide further insight into the levitation mechanism. The three-dimensional scalar potential distribution (**Figure S23b**) reveals a strong vertical gradient that supports the lifting force. A cross-sectional view (**Figure S23c**) highlights the balance of repulsive forces above the magnet, where the levitated composite is confined within a stable potential well.

These results confirm that MGCs not only achieve passive levitation but also exhibit structural adaptability, with interfacial entanglement and nanosheet confinement contributing to enhanced stability against perturbations. The integration of experimental observation with field simulations establishes a mechanistic basis for exploiting diamagnetic levitation as a stringent test of composite functionality.


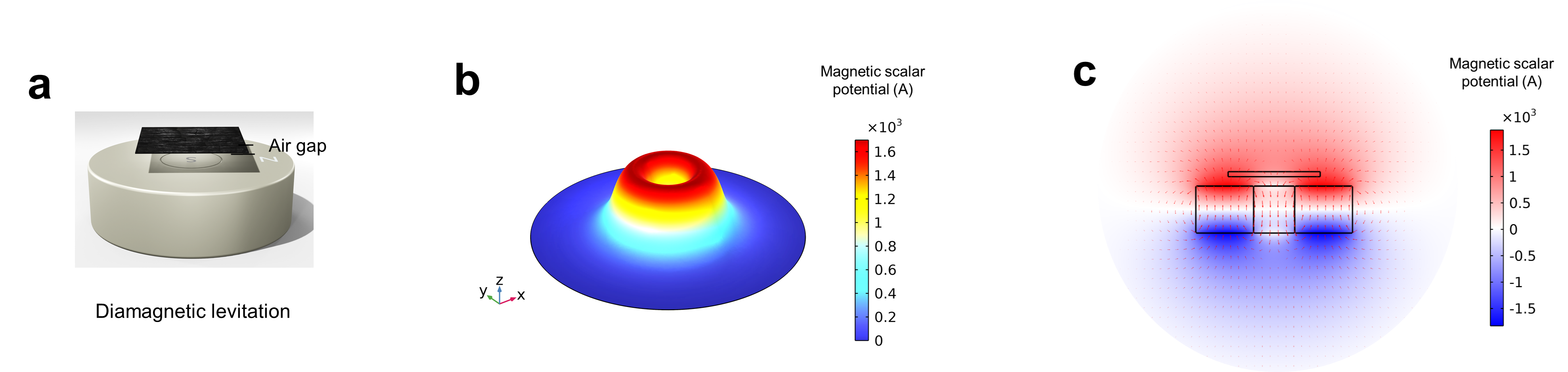


**Figure S23 The MGCs diamagnetic levitation under circular magnet. a**. Schematic illustration of the diamagnetic levitation setup, where an air gap is formed between the MGC sample and the circular permanent magnet. The array consists of a ring magnet (outer radius of 10 mm, inner radius of 3 mm and thickness of 6 mm) and a cylinder magnet (radius of 3 mm and thickness of 6 mm). **b**. Simulated three-dimensional distribution of the magnetic scalar potential, showing the field gradient that supports levitation. **c**. Cross-sectional view of the magnetic scalar potential, highlighting the vertical force balance and confinement of the levitating sample.

**Note 9. Self-healing and regenerative behavior of MGCs.**

**Figure S24** demonstrates the intrinsic ability of MGCs to undergo self-healing and regeneration through active mycelial growth. In **Figure S24a** and **S24b**, two fractured blocks that are initially separated gradually reconnect: after just 2 days, hyphae begin to span the gap, and by day 5 a continuous network is established. When the partially healed sample is immersed in a PEG–graphene/PVA nutrient solution, the process accelerates, and within 3 additional days the system consolidates into a bulk-integrated composite.

A different geometry is demonstrated in **Figure S24c**, where two blocks stacked longitudinally undergo vertical fusion. Here, mycelium grows across the interface within 2 days, yielding a mechanically continuous structure by day 5. Subsequent regrowth in nutrient solution produces an enlarged, unified composite.

Even more strikingly, **Figure S24d** reveals the regenerative capacity of long-term stored materials: MGCs kept at room temperature for 3 months, once reintroduced into nutrient-rich medium, resume mycelial proliferation and regenerate into new composites.


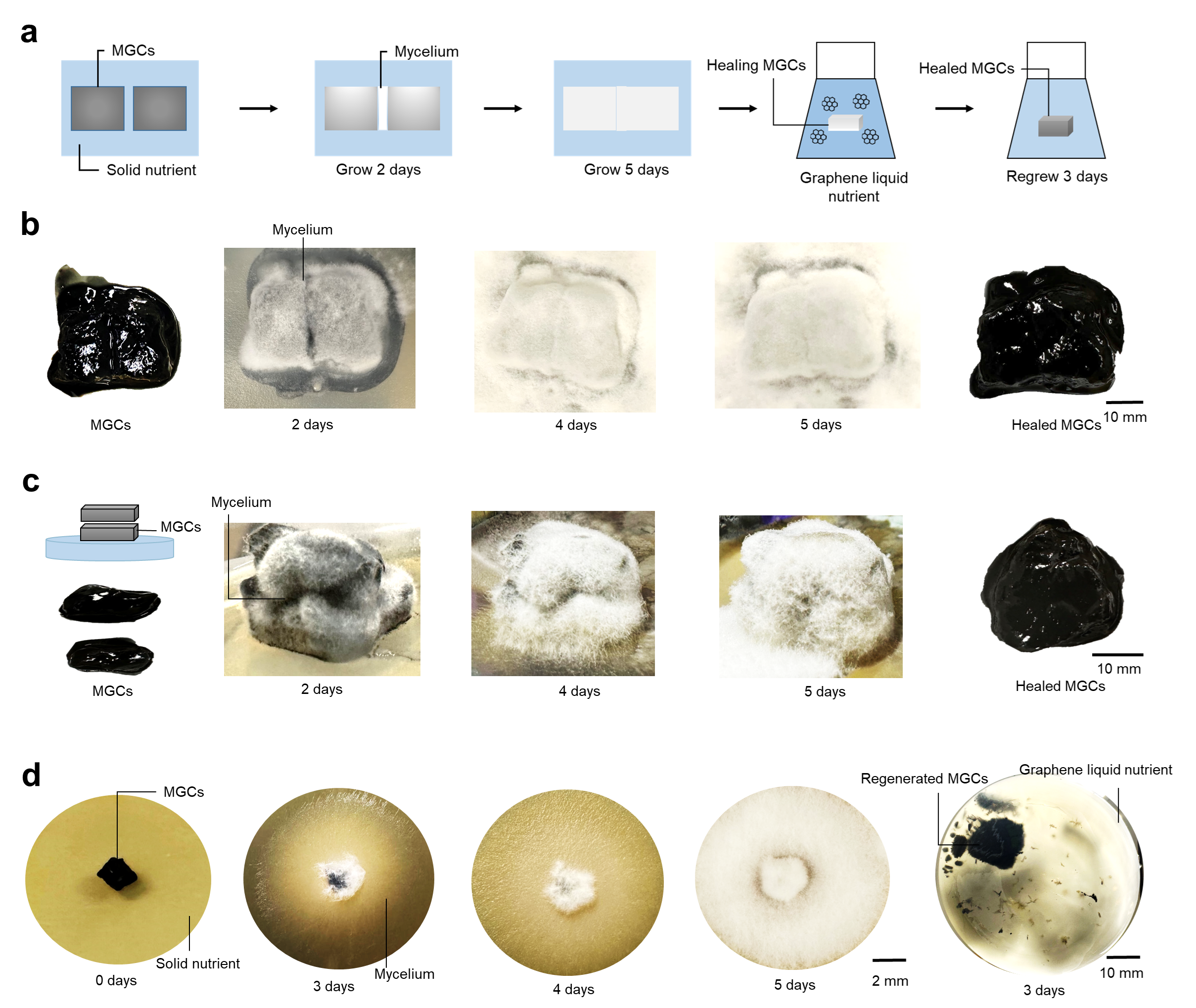


**Figure S24 Self-healing and regenerative behavior of MGCs. a**. Schematic of the self-healing protocol. Two separated MGCs pieces are placed on solid nutrient medium; newly grown mycelium bridges the gap after 2 days and the interface becomes sutured by day 5. The partially healed construct is then immersed in a liquid nutrient containing 20% PEG-intercalated graphene (PEG–graphene) and 10% PVA; within 3 days, hyphal regrowth and phase separation regenerate a continuous bulk MGC. **b**. Time-lapse images of two adjacent MGCs undergoing healing. Mycelium appears in the interfacial gap at 2 days, fills the wound by day 4, and forms a continuous junction by day 5. After 3 days of regrowth in the PEG–graphene nutrient, the interface is fully repaired. **c.** Healing of stacked MGCs. Two blocks placed longitudinally exhibit mycelial overgrowth at the interface after 2 days, become interconnected by day 4, and form a continuous bulk by day 5; subsequent 3-day regrowth in PEG–graphene nutrient yields a larger integrated MGCs. **d**. Long-term regeneration. MGCs fragments stored at room temperature for ~3 months and then placed on solid medium show renewed mycelial outgrowth and ultimately form new bulk MGCs.

**Note 10. Reproducibility and stability of MGCs**

Ensuring batch-to-batch reproducibility and environmental stability is essential for the practical deployment of biosynthetically assembled composite materials. We therefore evaluated the mechanical and levitation performance of MGCs across independently cultured batches and under conditions relevant to storage and environmental exposure.

**Reproducibility assessment.** Three independent batches of MGCs are prepared under identical substrate composition, inoculation density, growth duration and post-processing conditions. Tensile strength, toughness and levitation height are measured using the same protocols, averaging five specimens per batch. As shown in **Figure S25a**, all three properties vary by less than two percent across batches, well within experimental uncertainty. This high level of reproducibility indicates that the bio-assisted nanosheet-pinned interfacial strategy operates with intrinsic process stability and consistently reconstructs uniform composite architectures across independent culture batches.

**Ambient storage stability.** Samples stored at 23 °C and 50% relative humidity for thirty days retain 98–99% of their initial strength, toughness and levitation performance (**Figure S25b**)**.** No measurable signs of structural deterioration, moisture-induced softening or microbial self-degradation are observed over this period. The excellent retention reflects the stability of the bio-assisted nanosheet-pinned interfacial strategy, in which growth-mediated entanglement and hydrogen-bonded junctions suppress post-growth microstructural relaxation.

**Humidity robustness.** When equilibrated at controlled relative humidity levels from 30% to 90%, the MGCs display modest and predictable reductions in mechanical and levitation performance but consistently preserve at least 97% of their baseline values (**Figure S25c**). This response reflects the inherent moisture uptake of the PVA–hypha matrix, whereas the preserved performance shows that the nanosheet-pinned interfacial network maintains mechanical integrity and adhesion under humidity loading.

These measurements collectively demonstrate that MGCs exhibit excellent biosynthetic reproducibility, long-term stability and environmental tolerance. The robustness of the growth-mediated assembly process supports the suitability of MGCs for scalable fabrication and application-relevant operating conditions.


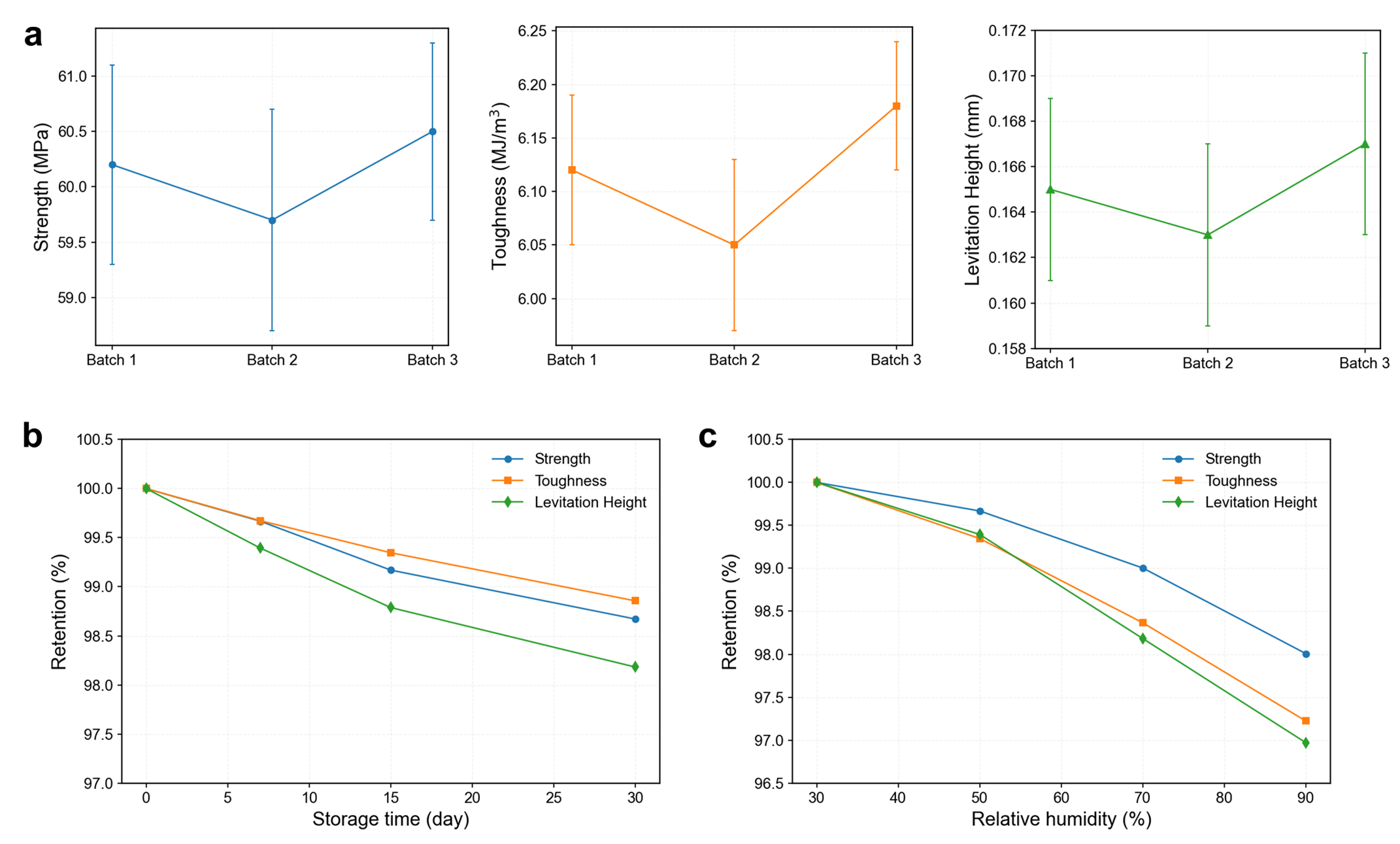


**Figure S25 Reproducibility and environmental stability of MGCs. a**. Batch-to-batch reproducibility. Tensile strength, toughness, and levitation height measured across three independently cultured batches show minimal variation (<2%), with the inter-batch differences falling well within the experimental error. These results confirm that the biosynthetic process yields highly consistent mechanical and functional properties despite the intrinsic biological variability of fungal growth. **b**. Ambient storage stability. Retention of strength, toughness, and levitation height during 30 days of storage at 23 °C and 50% relative humidity. All properties remain above 98–99% of their initial values, indicating excellent stability without signs of microbial degradation or structural collapse. **c**. Humidity robustness. Retention of mechanical and levitation performance after exposure to environments ranging from 30% to 90% relative humidity. Even under 90% RH, MGCs maintain ≥97% of their original properties, demonstrating good environmental tolerance and moisture-induced structural resilience.

**Note 11. Programmable levitated micro robot**
We employ non-contact laser irradiation to propel the motion of MGCs above a magnet array and regulate their trajectories by tailoring the magnetic field distribution, effectively transforming them into programmable levitated micro-robots. This design strategy is guided by finite element method (FEM) simulations, with results summarized in **Figures S26** and **S27**.

The simulations in **Figure S26** reveal the dynamic coupling between localized photothermal excitation and diamagnetic levitation. Upon laser irradiation, the temperature of the composite plate gradually increases (**Figure S26a** and **Video S4**), which reduces the diamagnetic force due to the temperature dependence of magnetic susceptibility. When the diamagnetic force (F_B_) decreases below the gravitational force (F_G_), an imbalance emerges that destabilizes the levitated state, thereby initiating motion. As shown in **Figure S26b**, the diamagnetic force exhibits a non-monotonic dependence on temperature, fluctuating around the gravitational threshold. This thermal modulation introduces a controllable driving mechanism for motion. The schematic in **Figure S26c** illustrates the principle: localized heating at one side of the plate generates anisotropic force distributions, producing tilting and directional displacement. In contrast to conventional mechanical actuation, this photothermal–magnetic coupling provides a contactless and reprogrammable actuation strategy for lightweight diamagnetic systems.

To further achieve directional control of MGCs, the magnetic field distribution is modulated by introducing a 0.1 mm gap between adjacent magnets (**Figure S27**). The FEM results show that this controlled gap reshapes the magnetic scalar potential landscape, creating localized field peaks that alter the magnetic gradient experienced by the levitated plate (**Figures S27a** and **S27b**). As a result, the diamagnetic force fluctuates around the gravitational equilibrium, and once the repulsion exceeds this balance, the plate transitions into a nonequilibrium state that drives motion (**Figure S27c**). Time-resolved simulations (**Figure S27d** and **Video S4**) demonstrate that such imbalances generate periodic displacement of the MGC plate, underscoring the dynamic tunability of diamagnetic levitation through engineered magnetic landscapes.

These results confirm the feasibility of regulating the locomotion of MGCs through a synergistic combination of laser-induced thermal modulation and programmable magnetic field design. This dual-control strategy establishes a generalizable platform for realizing levitated, contactless, and reconfigurable microrobotic systems.


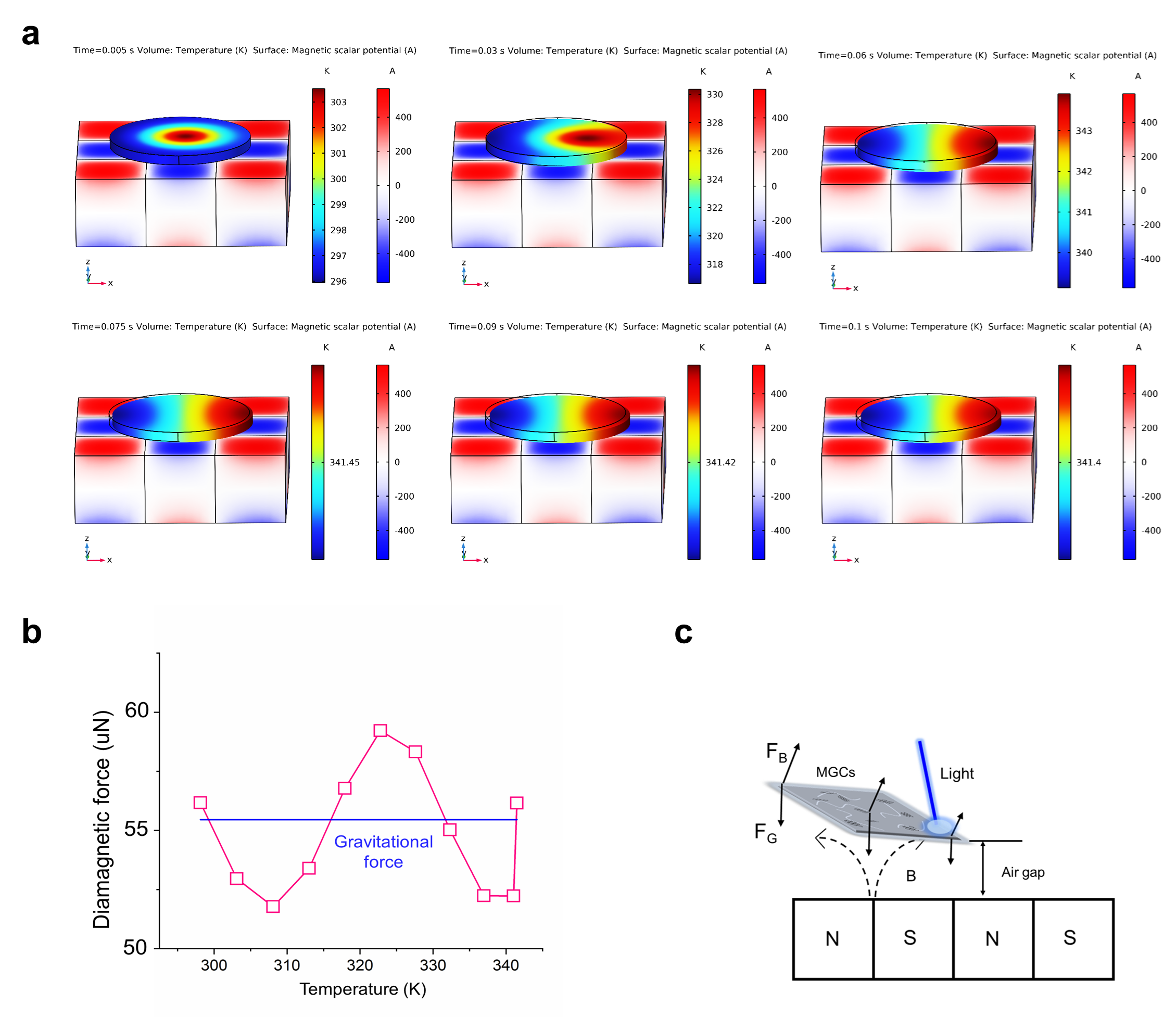


**Figure S26 Simulation of laser-driven motion of MGC plates. a**. Time-resolved magnetic potential distributions above a square magnet array during localized laser heating. Increasing temperature reduces the diamagnetic force below the gravitational force, leading to destabilization and motion initiation. **b**. Variation of diamagnetic force with increasing plate temperature, showing a crossover with gravitational force that induces motion. **c**. Schematic illustration of the laser-driven actuation mechanism. A localized laser spot heats one side of the levitating MGC plate, generating anisotropic diamagnetic force (F_B_) relative to gravity (F_G_). This imbalance tilts the plate and drives directional motion above the 3 × 3 × 3 mm^3^ magnet array.


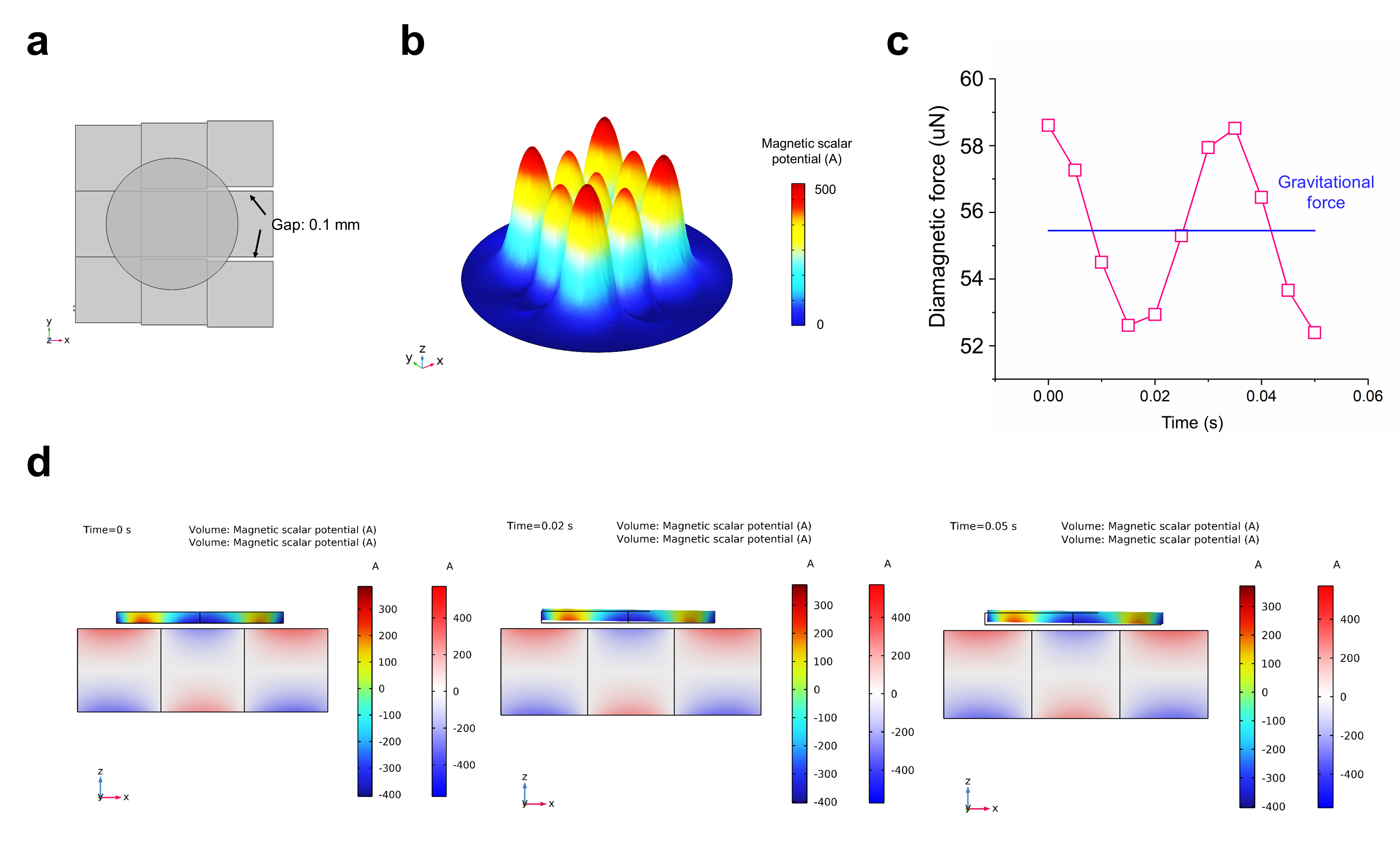


**Figure S27 Simulation of programmable magnetic field distribution and induced motion of MGCs. a**. Schematic of the magnet array with an introduced 0.1 mm gap between adjacent magnets to reconfigure the magnetic field distribution. **b**. Simulated magnetic scalar potential map showing that the inserted gap generates localized peaks in field intensity, creating a modulated magnetic landscape. **c**. Evolution of diamagnetic force over time under the programmed field distribution. Adjusting the inter-magnet spacing alters the field gradient and thereby the magnitude of diamagnetic forces. When the MGC plate approaches the magnet array, the enhanced repulsion exceeds the gravitational force, driving the system into a nonequilibrium state and triggering plate motion. **d**. Time-resolved simulations of the MGC plate motion (t = 0, 0.02, and 0.05 s), with magnetic scalar potential profiles shown below. The asymmetric field distribution leads to periodic force imbalance, which induces controlled displacement of the plate.

**Note 12. Quantification of experimental efficiency gains**

**Calculation framework.** To rigorously quantify the efficiency advantage of the proposed machine learning (ML) assisted workflow, we compare it against a conventional design-of-experiments (DoE) strategy that uniformly samples the composition–processing–property space. The DoE baseline is constructed as a full-factorial mixture design with three experimental variables (e.g., graphene content, PVA content, and growth duration) at five levels each, yielding 5^3^=1255 primary conditions. To ensure statistical reproducibility, coverage of edge cases, and inclusion of replicates and center points, the DoE plan expands to approximately 400 experiments, consistent with the number typically required for robust response-surface modeling in three-factor mixture systems.

**ML workflow sampling.** The ML pipeline consists of: (i) an initial space-filling design of 30 uniformly distributed points, (ii) four active-learning (AL) iterations, each contributing five new Pareto-optimal or high-value points (20 total), and (iii) a final validation set of 10 confirmatory experiments near the predicted Pareto front. In total, about 60 experiments are required to achieve >95 % coverage of the estimated Pareto-front hypervolume (**Figure S9**).

**Efficiency metrics and formulas.**
Five quantitative metrics are used to evaluate efficiency:

1. Number of experiments:

$N=N_{\mathrm{initial}}+N_{\mathrm{AL}}+N_{\mathrm{validation}}$ $N_{\mathrm{DoE}}=400$ , $N_{\mathrm{ML}}\approx60$

2. Cumulative experimental days:

$T_{\mathrm{cumulative}}=N\times t_{\mathrm{sample}}$ , $t_{\mathrm{sample}}=10 days/experiment$

3. Overall project duration (including parallelization and fixed overheads):

$T_{\mathrm{project}}=t_{\mathrm{fixed}}+\left[ \frac{N}{n_{\mathrm{parallel}}} \right]\times t_{\mathrm{sample}}$ , $n_{\mathrm{parellel}}=5$

4. Estimated cost:

$C=C_{\mathrm{fixed}}+N\times c_{\mathrm{sample}}$ , $c_{\mathrm{sample}}= \$ 80/experiment.$

5. Labor (person-hours):

$L=L_{\mathrm{fixed}}+N\times l_{\mathrm{sample}}$ , $l_{\mathrm{sample}}= 2 h/experiment.$

**Reduction calculation.**

For each metric $X$, the relative reduction is calculated as:

$$Reduction (\%)=\frac{X_{\mathrm{DoE}}-X_{\mathrm{ML}}}{X_{\mathrm{DoE}}}\times100\%$$

**Results.**
Including fixed overheads such as planning and setup days (20 for DoE versus 15 for ML), fixed costs ($2000 versus $4000 including model training), and fixed labor (50 versus 60 h), the ML-assisted workflow achieves substantial efficiency gains. The number of experiments and cumulative experimental days are both reduced by approximately 85 %, while the overall project duration is shortened by about 83.5 % as a result of parallelization. The total cost decreases by roughly 74.1 %, and the required labor is lowered by approximately 78.8 %. These results collectively demonstrate that the ML-guided approach significantly reduces experimental burden, time, and resource consumption, thereby accelerating the exploration–validation cycle. All calculations and their visual representation are depicted in **Figure S28**.


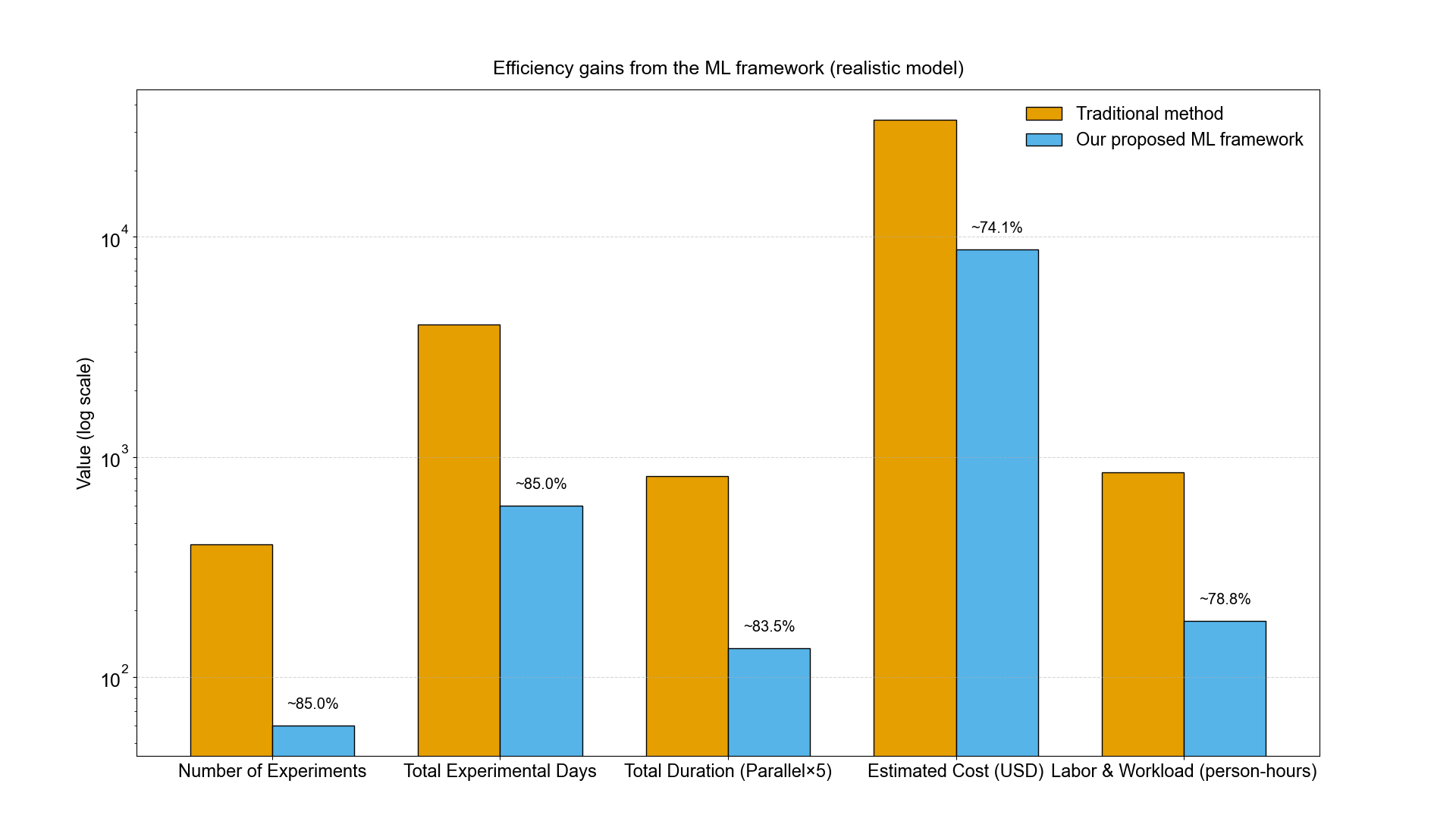


**Figure S28 Comparison between a traditional experimental approach and our proposed machine learning (ML) framework.** Comparison of the proposed ML-guided strategy with a conventional DoE baseline (≈400 experiments) across five efficiency metrics. Incorporating planning/setup time, fixed costs, and labor overheads, the ML workflow converges after ≈60 experiments, reducing the number of experiments and cumulative experimental days by ~85 %, shortening overall project duration by ≈83.5 %, and lowering total cost and labor requirements by ≈74.1 % and ≈78.8 %, respectively. These results highlight the substantial reduction in experimental burden and resources, enabling a faster and more sustainable exploration–validation cycle. Furthermore, the ML framework accurately targets the Pareto front, leading to a more representative coverage of high-performance solutions in the design space.

**Note 13. Benchmarking protocol for the machine learning framework**

To quantitatively evaluate the proposed ML framework, we construct a retrospective benchmark against representative multi-objective optimization (MOO) baselines under a fixed and common experimental budget. All methods share the same initialization of 30 experimental data points and then perform 20 additional sequential selections, yielding a total budget of 50 experiments. The comparison therefore focuses on the 30–50 experiment window, which isolates the contribution of sequential decision-making beyond the common initialization stage. This benchmark setting is chosen to reflect the practical workflow adopted in this study while maintaining consistency across methods.

The benchmark includes Random Search [22], Latin hypercube sampling (LHS) [23], Weighted Multi-Objective Bayesian Optimization (Weighted-MOBO) [24, 25], an NSGA-II-inspired baseline based on the Non-dominated Sorting Genetic Algorithm II (NSGA-II) [26], a ParEGO-style baseline based on Pareto Efficient Global Optimization (ParEGO) [27], an EHVI-style baseline based on Expected Hypervolume Improvement (EHVI) [28], and the experimentally executed PSL–AL trajectory. Random Search and LHS serve as non-adaptive baselines, with LHS following the logic of space-filling design. Weighted-MOBO represents a scalarization-based Bayesian optimization (BO) baseline motivated by general surrogate-based BO frameworks for expensive black-box optimization. The NSGA-II baseline represents evolutionary Pareto optimization based on non-dominated sorting and diversity preservation. The ParEGO and EHVI baselines represent scalarization-based and hypervolume-based MOBO strategies, respectively. The proposed PSL–AL framework is evaluated using the experimentally executed AL trajectory.

To define a common candidate space for all methods, the benchmark pool includes (i) the 20 experimentally evaluated AL points and (ii) additional synthetic candidate formulations generated within the observed design bounds. The objective values of the synthetic candidates are estimated using a Random Forest (RF) surrogate model trained on the full set of 50 experimentally measured data points. This oracle-style candidate pool is used only for retrospective benchmarking, so that all methods are evaluated over the same search space under identical selection budgets.

**Performance metrics.** Several complementary metrics are used to evaluate final Pareto-front quality and sequential sample efficiency. Hypervolume (HV) is used to quantify the overall quality of the non-dominated set identified at each experimental budget. Objective values are first normalized by Min–Max scaling, and HV is then computed in the normalized objective space using a fixed reference point. As a standard metric in multi-objective optimization, HV jointly reflects convergence and spread of the Pareto set [27]. Reference Pareto-front coverage is defined on a reference Pareto set constructed over the benchmark candidate pool and quantifies, at each experimental budget, the fraction of reference Pareto points recovered by the non-dominated set within a component-wise tolerance of 0.06 in normalized objective space. A balanced subset of the reference Pareto front is further defined in normalized objective space to represent balanced trade-off solutions; in the present benchmark, this subset consists of reference Pareto solutions for which all three normalized objectives are at least 0.75. Balanced reference-front coverage is then defined as the fraction of this subset recovered by the non-dominated set using the same component-wise tolerance of 0.06. To characterize sequential optimization efficiency over the full trajectory rather than only at the final budget, the area under the curve (AUC) is additionally calculated for HV and coverage over the 30–50 experiment window, reported as HV-AUC and coverage-AUC, respectively. Time-to-target is further defined as the number of experiments required to reach 90% of the final HV achieved by PSL–AL.

**Results.** The optimization trajectories are shown in **Figure S29**, and the corresponding quantitative summary is provided in **Table S6**. As shown in **Figure S29a**, PSL–AL exhibits the fastest increase in hypervolume (HV) and achieves the highest final HV among all compared methods. Consistently, **Table S6** shows that PSL–AL attains the best overall HV-based performance, with HV_final = 0.81180 and HV-AUC = 15.43780. The advantage of PSL–AL is further supported by the reference Pareto-front coverage results in **Figure S29b**, where PSL–AL reaches complete reference-front coverage at the final budget and achieves the highest cumulative coverage efficiency (Coverage_final = 1.000000, Coverage-AUC = 19.600000). A similar trend is observed for balanced reference-front coverage in **Figure S29c**, where PSL–AL is the only method that reaches full balanced coverage within the tested budget.

The sequential sample-efficiency advantage of PSL–AL is further reflected by the Experiments to 90% final HV metric in **Table S6**. PSL–AL reaches this target after 33.0 experiments, earlier than Weighted-MOBO (36.0), NSGA-II (38.5), ParEGO (43.7), and EHVI (45.0), while Random Search and LHS do not reach this threshold within the tested budget. These results show that PSL–AL provides the strongest overall combination of Pareto-front quality, front reconstruction capability, and sequential sample efficiency under the same benchmark setting.


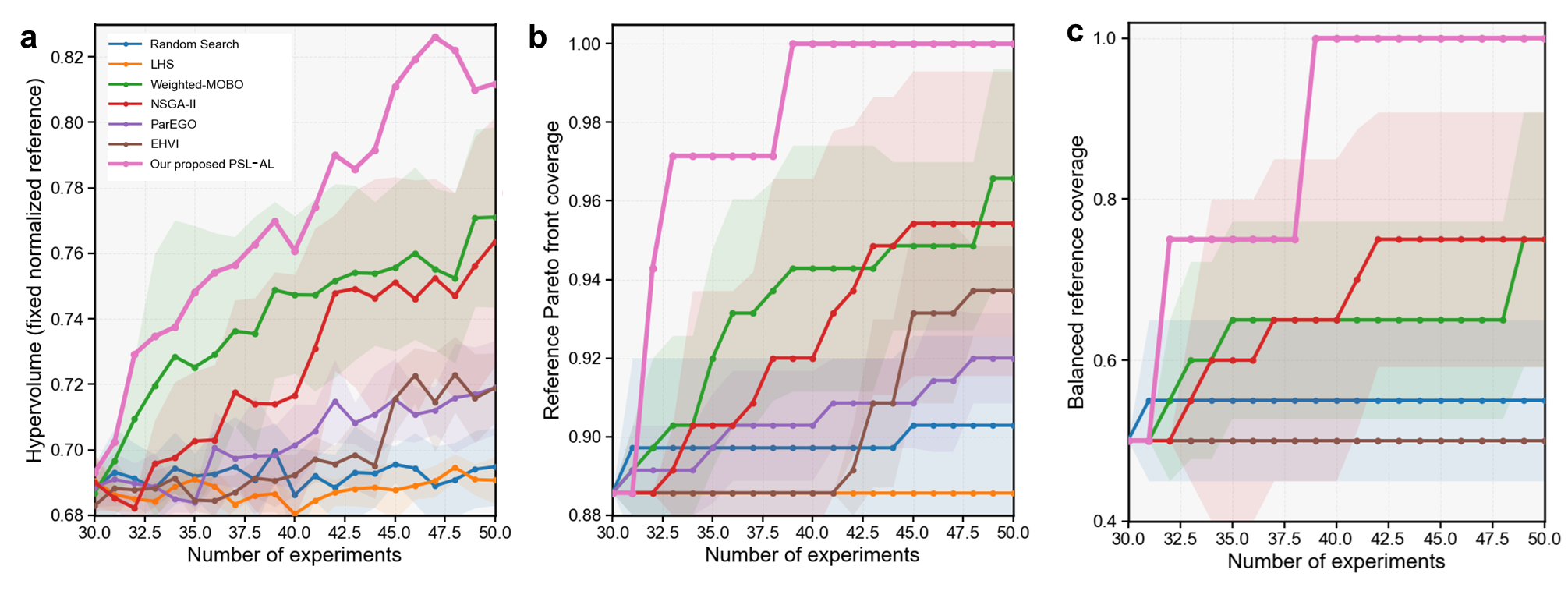


**Figure S29 Quantitative benchmark of the proposed ML framework against representative multi-objective optimization baselines. a.** Evolution of hypervolume (HV, normalized to a fixed reference point) as a function of the number of experiments for different optimization strategies, including Random Search, LHS, Weighted-MOBO, NSGA-II, ParEGO, EHVI, and the proposed PSL–AL framework. All methods start from the same initial 30 experimental data points, followed by 20 additional sequential selections, such that the comparison is performed over the 30–50 experiment window under an identical total budget. Solid lines denote the mean values over repeated runs, and shaded regions represent the standard deviation. **b.** Evolution of reference Pareto-front coverage with increasing experimental budget. This metric quantifies the extent to which the solutions identified by each method recover the reference Pareto front defined over the benchmark candidate pool under the same sequential sampling budget. **c.** Evolution of balanced reference-front coverage with increasing experimental budget. This metric quantifies the recovery of reference Pareto solutions satisfying balanced high-performance criteria and provides a practically relevant measure of sequential sample efficiency.

**Table S6 Quantitative benchmark comparison of the proposed ML framework and representative multi-objective optimization baselines**

| Method | HV_final | Coverage_final | HV_AUC | Coverage_AUC | Experiments to 90% final HV |
| --- | --- | --- | --- | --- | --- |
| Random Search | 0.69480 | 0.902857 | 13.84414 | 17.968571 | — |
| LHS | 0.69068 | 0.885714 | 13.75132 | 17.714286 | — |
| Weighted-MOBO | 0.77104 | 0.965714 | 14.80570 | 18.662857 | 36.0 |
| NSGA-II | 0.76360 | 0.954286 | 14.48248 | 18.497143 | 38.5 |
| ParEGO | 0.71896 | 0.920000 | 14.04812 | 18.091429 | 43.7 |
| EHVI | 0.71892 | 0.937143 | 13.96424 | 18.031429 | 45.0 |
| Our proposed PSL–AL | 0.81180 | 1.000000 | 15.43780 | 19.600000 | 33.0 |


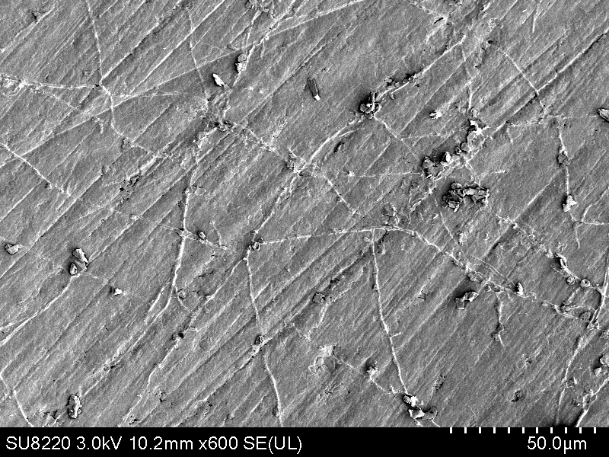


**Figure S30** **SEM image of the MGCs.** The surface exhibits a relatively uniform morphology with continuous fungal fibrils distributed across the observed region.





**Figure S31 TEM image of MXene nanosheet.**


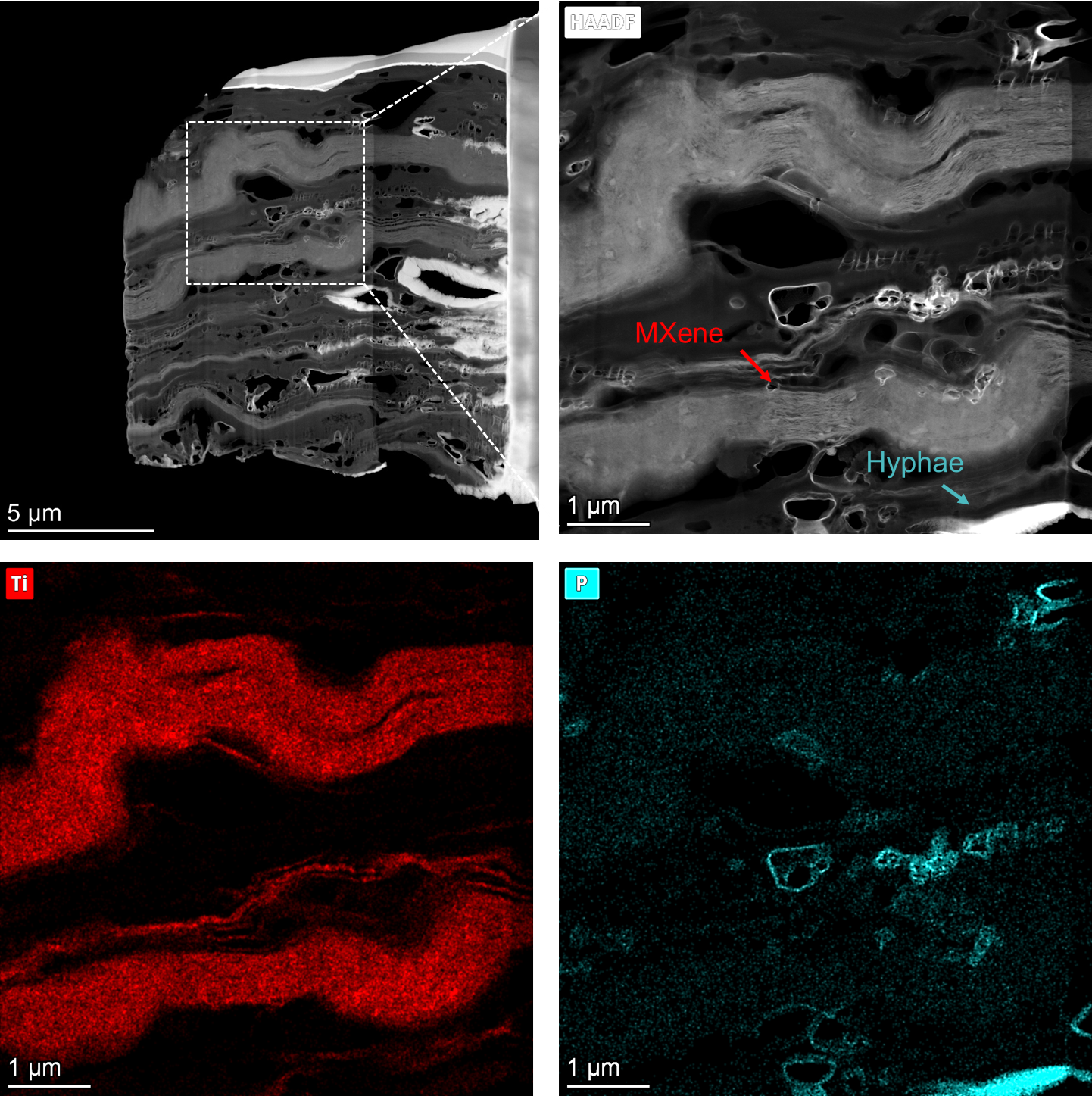


**Figure S32 Hierarchical interfacial architecture of MXene–mycelium composites (MMCs).** HAADF-STEM images (top left and right) reveal a lamellar architecture where MXene nanosheets (red arrows) are embedded within the mycelial matrix. The fungal hyphae (cyan arrows) intimately entangle with MXene, forming pinned-and-bridged domains. Elemental mapping (bottom panels) confirms the spatial distribution of Ti (red), a characteristic element of MXene, and P (cyan), which is enriched in fungal biomass, highlighting the co-localization of inorganic nanosheets and organic hyphae. This spatial overlap evidences the construction of bio–inorganic interfacial networks.

**References**

1. B. Yan, S. Liu, Y. Yuan, X. Hou, M. Zhou, Y. Yu, Q. Wang, C. He, P. Wang. Polymer-regulating MXene@dopamine electroactive gel-inks for textile-based multi-protective wearables. *Adv. Funct. Mater.* **34**, 2401097 (2024).
2. Q. Zhang, H. Li. MOEA/D: A multiobjective evolutionary algorithm based on decomposition. *IEEE Trans. Evol. Comput.* **11**, 712–731 (2007).
3. B. Shahriari, K. Swersky, Z. Wang, R. P. Adams, N. de Freitas. Taking the human out of the loop: A review of Bayesian optimization. *Proc. IEEE* **104**, 148–175 (2016).
4. F. Di Fiore, M. Nardelli, L. Mainini. Active learning and Bayesian optimization: A unified perspective to learn with a goal. *Arch. Comput. Methods Eng.* (2024).
5. D. Sharma, H. Le Ferrand. 3D printed gyroid scaffolds enabling strong and thermally insulating mycelium-bound composites for greener infrastructures. *Nat. Commun.* **16**, 5775 (2025).
6. S. Gantenbein, E. Colucci, J. Käch, et al. Three-dimensional printing of mycelium hydrogels into living complex materials. *Nat. Mater.* **22**, 128–134 (2023).
7. E. Camilleri, S. Narayan, D. Lingam, R. Blundell. Mycelium-based composites: An updated comprehensive overview. *Biotechnol. Adv.* **79**, 108517 (2025).
8. M. Jones, A. Mautner, S. Luenco, A. Bismarck, S. John. Engineered mycelium composite construction materials from fungal biorefineries: A critical review. *Mater. Des.* **187**, 108397 (2020).
9. E. Elsacker, M. Zhang, M. Dade-Robertson. Fungal engineered living materials: The viability of pure mycelium materials with self-healing functionalities. *Adv. Funct. Mater.* **33**, 2301875 (2023).
10. E. Roumeli, R. Hendrickx, L. Bonanomi, A. Vashisth, K. Rinaldi, C. Daraio. Biological matrix composites from cultured plant cells. *Proc. Natl. Acad. Sci. U.S.A.* **119**, e2119523119 (2022).
11. Y. J. Qiu, et al. Coassembly of hybrid microscale biomatter for robust, water-processable, and sustainable bioplastics. *Sci. Adv.* **11**, eadr1596 (2025).
12. J. Su, K. Zhao, Y. Ren, L. Zhao, B. Wei, B. Liu, Y. Zhang, F. Wang, J. Li, Y. Liu, K. Liu, H. Zhang. Biosynthetic structural proteins with super plasticity, extraordinary mechanical performance, biodegradability, biocompatibility and information storage ability. *Angew. Chem. Int. Ed.* **61**, e202117538 (2022).
13. H. Lyer, P. Grandgeorge, A. M. Jimenez, I. R. Campbell, M. Parker, M. Holden, M. Venkatesh, M. Nelsen, B. Nguyen, E. Roumeli. Fabricating strong and stiff bioplastics from whole spirulina cells. *Adv. Funct. Mater.* **33**, 2302067 (2023).
14. P. Xie, Y. Ge, Y. Wang, J. Zhou, Y. Miao, Z. Liu. Mechanically enhanced nanocrystalline cellulose/reduced graphene oxide/polyethylene glycol electrically conductive composite film. *Nanomaterials* **12**, 4371 (2022).
15. S. Farah, D. G. Anderson, R. Langer et al. Physical and mechanical properties of PLA and their functions in widespread applications: a comprehensive review. *Adv. Drug Deliv. Rev.* **107**, 367–392 (2016).
16. M. Chougan, S. H. Ghaffar, M. J. Al-Kheetan. Graphene-based nano-functional materials for surface modification of wheat straw to enhance the performance of bio-based polylactic acid composites. *Mater. Today Sustainability* **21**, 100308 (2023).
17. S. Ye, B. Chen, J. Feng. Fracture mechanism and toughness optimization of macroscopic thick graphene oxide film. *Sci. Rep.* **5**, 13102 (2015).
18. S. Yang, Y. Zhang, Y. L. Liu, T. Gu, F. Liu. Flexible cellulose nanofiber–Fe₃O₄/liquid-metal/graphene composite films with hierarchical gradient structure for efficient electromagnetic interference shielding and thermal management. *Compos. Part B Eng.* **287**, 111844 (2024).
19. S. Guo, J. Chen, Y. Zhang et al. Graphene-based films: fabrication, interfacial modification and applications. *Nanomaterials* **11**, 10 (2021).
20. X. F. Chen, et al. Diamagnetic composites for high-Q levitating resonators. *Adv. Sci.* **9**, 2203619 (2022).
21. X. F. Chen, et al. Rigid body dynamics of diamagnetically levitating graphite resonators. *Appl. Phys. Lett.* **116**, 244102 (2020).
22. L. Li, A. Talwalkar. Random search and reproducibility for neural architecture search. *Proc. 3rd AutoML Workshop at ICML, PMLR* **115**, 201–212 (2020).
23. L. Chen, C. Chen, L. Wang, W. Zeng, Z. Li. Uncertainty quantification of once-through steam generator for nuclear steam supply system using Latin hypercube sampling method. *Nucl. Eng. Technol.* **55**, 2395–2406 (2023).
24. S. Belakaria, A. Deshwal, J. R. Doppa. Max-value entropy search for multi-objective Bayesian optimization. *Adv. Neural Inf. Process. Syst.* 32 (2019).
25. S. Daulton, M. Balandat, E. Bakshy. Differentiable expected hypervolume improvement for parallel multi-objective Bayesian optimization. *Adv. Neural Inf. Process. Syst.* 33 (2020).
26. S. Belakaria, A. Deshwal, N. K. Jayakodi, J. R. Doppa. Uncertainty-aware search framework for multi-objective Bayesian optimization. *Proc. AAAI Conf. Artif. Intell.* **34**, 10044–10052 (2020).
27. M.T.M. Emmerich, A.H. Deutz. A tutorial on multiobjective optimization: fundamentals and evolutionary methods. *Nat. Comput.* **17**, 585–609 (2018).
28. Paulson, J. A. Bayesian optimization as a flexible and efficient design strategy for next-generation process systems. arXiv: 2401.16373.

**Video S1. Diamagnetic levitation behavior.**

Demonstrating that MGCs diamagnetic behavior in circular magnets composed of N-pole and S pole N52 neodymium permanent magnets, as well as in square magnets, at room temperature. The circular magnet consists of a ring magnet (outer radius of 10 mm, inner radius of 5 mm and thickness of 6 mm). The square magnets consists of an array of magnets with size of 3 x 3 x 3 mm^3^.

**Video S2. Simulation of diamagnetic levitation behaviors.**

Demonstrating the process of simulating MGCs square plate being levitated on a square magnet array. And the MGCs square plate being levitated on a circular magnet array. The array consists of a ring magnet (outer radius of 10 mm, inner radius of 3 mm and thickness of 6 mm) and a cylinder magnet (radius of 3 mm and thickness of 6 mm).

**Video S3. Three dimension CT scan results of MGCs.**

Displaying the main material of MGCs in a three-dimensional layered orderly distribution.

**Video S4. FEM simulation of laser drive diamagnetic levitation micro robot moving.**

Illustrating that MGCs exhibit movement behavior when locally heated by a laser. Demonstrating the simulation of controlling the movement trajectory of the micro robot by adjusting the magnet gap through regulating the magnetic field distribution.

**Video S5. Diamagnetic levitation micro robot.**

Demonstrating the levitated movement behavior of MGCs when locally heated by laser. The square magnets consists of an array of magnets with size of 3 x 3 x 3 mm^3^. Illustrating that control the movement trajectory of a levitated micro robot by programming the magnetic field distribution. The square magnets consists of an array of magnets with size of 3 x 3 x 3 mm^3^. Demonstrating the adjustment of the magnets into a circle, followed by programming the magnetic field, displaying the levitated rotation driven by the laser. The array consists of a ring magnet (outer radius of 10 mm, inner radius of 3 mm and thickness of 6 mm) and a cylinder magnet (radius of 3 mm and thickness of 6 mm).
